# Isosorbide Mononitrate and Cilostazol Treatment in Patients With Symptomatic Cerebral Small Vessel Disease: The Lacunar Intervention Trial-2 (LACI-2) Randomized Clinical Trial

**DOI:** 10.1001/jamaneurol.2023.1526

**Published:** 2023-05-24

**Authors:** Joanna M. Wardlaw, Lisa J. Woodhouse, Iris I. Mhlanga, Katherine Oatey, Anna K. Heye, John Bamford, Vera Cvoro, Fergus N. Doubal, Timothy England, Ahamad Hassan, Alan Montgomery, John T. O’Brien, Christine Roffe, Nikola Sprigg, David J. Werring, Philip M. Bath

**Affiliations:** 1 Centre for Clinical Brain Sciences, UK Dementia Research Institute, University of Edinburgh, Edinburgh, United Kingdom; 2 Stroke Trials Unit, Mental Health and Clinical Neuroscience, University of Nottingham, Nottingham, United Kingdom; 3 Edinburgh Clinical Trials Unit, Usher Institute, University of Edinburgh, Edinburgh, United Kingdom; 4 Department of Neurology, Leeds General Infirmary, Leeds, United Kingdom; 5 Victoria Hospital, National Health Service Fife, Kirkcaldy, United Kingdom; 6 Nottingham Clinical Trials Unit, University of Nottingham, Nottingham, United Kingdom; 7 Department of Psychiatry, University of Cambridge School of Clinical Medicine, Cambridge, United Kingdom; 8 Stroke Research, Keele University, Stoke-on-Trent, United Kingdom; 9 Stroke Research Centre, University College London Queen Square Institute of Neurology, Russell Square House, London, United Kingdom

## Abstract

**Importance:**

Cerebral small vessel disease (cSVD) is a common cause of stroke (lacunar stroke), is the most common cause of vascular cognitive impairment, and impairs mobility and mood but has no specific treatment.

**Objective:**

To test the feasibility, drug tolerability, safety, and effects of 1-year isosorbide mononitrate (ISMN) and cilostazol treatment on vascular, functional, and cognitive outcomes in patients with lacunar stroke.

**Design, Setting, and Participants:**

The Lacunar Intervention Trial-2 (LACI-2) was an investigator-initiated, open-label, blinded end-point, randomized clinical trial with a 2 × 2 factorial design. The trial aimed to recruit 400 participants from 26 UK hospital stroke centers between February 5, 2018, and May 31, 2021, with 12-month follow-up. Included participants had clinical lacunar ischemic stroke, were independent, were aged older than 30 years, had compatible brain imaging findings, had capacity to consent, and had no contraindications to (or indications for) the study drugs. Data analysis was performed on August 12, 2022.

**Interventions:**

All patients received guideline stroke prevention treatment and were randomized to ISMN (40-60 mg/d), cilostazol (200 mg/d), ISMN-cilostazol (40-60 and 200 mg/d, respectively), or no study drug.

**Main Outcomes:**

The primary outcome was recruitment feasibility, including retention at 12 months. Secondary outcomes were safety (death), efficacy (composite of vascular events, dependence, cognition, and death), drug adherence, tolerability, recurrent stroke, dependence, cognitive impairment, quality of life (QOL), and hemorrhage.

**Results:**

Of the 400 participants planned for this trial, 363 (90.8%) were recruited. Their median age was 64 (IQR, 56.0-72.0) years; 251 (69.1%) were men. The median time between stroke and randomization was 79 (IQR, 27.0-244.0) days. A total of 358 patients (98.6%) were retained in the study at 12 months, with 257 of 272 (94.5%) taking 50% or more of the allocated drug. Compared with those participants not receiving that particular drug, neither ISMN (adjusted hazard ratio [aHR], 0.80 [95% CI, 0.59 to 1.09]; *P* = .16) nor cilostazol (aHR, 0.77 [95% CI, 0.57 to 1.05]; *P* = .10) alone reduced the composite outcome in 297 patients. Isosorbide mononitrate reduced recurrent stroke in 353 patients (adjusted odds ratio [aOR], 0.23 [95% CI, 0.07 to 0.74]; *P* = .01) and cognitive impairment in 308 patients (aOR, 0.55 [95% CI, 0.36 to 0.86]; *P* = .008). Cilostazol reduced dependence in 320 patients (aHR, 0.31 [95% CI, 0.14 to 0.72]; *P* = .006). Combination ISMN-cilostazol reduced the composite (aHR, 0.58 [95% CI, 0.36 to 0.92]; *P* = .02), dependence (aOR, 0.14 [95% CI, 0.03 to 0.59]; *P* = .008), and any cognitive impairment (aOR, 0.44 [95% CI, 0.23 to 0.85]; *P* = .02) and improved QOL (adjusted mean difference, 0.10 [95% CI, 0.03 to 0.17]; *P* = .005) in 153 patients. There were no safety concerns.

**Conclusions and Relevance:**

These results show that the LACI-2 trial was feasible and ISMN and cilostazol were well tolerated and safe. These agents may reduce recurrent stroke, dependence, and cognitive impairment after lacunar stroke, and they could prevent other adverse outcomes in cSVD. Therefore, both agents should be tested in large phase 3 trials.

**Trial Registration:**

ClinicalTrials.gov Identifier: NCT03451591

## Key Points

**Question:**

Can modulators of cerebrovascular endothelial function, including isosorbide mononitrate (ISMN; a nitric oxide donor) and cilostazol (a phosphodiesterase-3 inhibitor), improve long-term outcomes after lacunar ischemic stroke?

**Findings:**

In this randomized clinical trial of 363 participants treated with ISMN, cilostazol, ISMN-cilostazol, or no study drug in a 2 × 2 factorial design, 99% of patients were retained at 1 year with good study drug adherence and no safety concerns. Isosorbide mononitrate reduced recurrent stroke and cognitive impairment, cilostazol reduced dependence, and ISMN-cilostazol reduced the composite of adverse vascular, dependence, and cognitive outcomes.

**Meaning:**

These 2 inexpensive licensed medications (ISMN and cilostazol) may reduce adverse long-term outcomes of lacunar ischemic stroke, a form of cerebral small vessel disease, and definitive trials are needed to confirm these findings.

## Teaser

This randomized clinical trial examines treatment outcomes with isosorbide mononitrate and cilostazol in patients with symptomatic cerebral small vessel disease.

## Introduction

Cerebral small vessel disease (cSVD) causes lacunar ischemic stroke (25% of ischemic strokes), vascular dementia, and neuropsychiatric and mood disorders and impairs mobility.^1,2^ Despite this disease burden, there is no specific treatment for cSVD.^3,4^ Although patients with lacunar ischemic stroke typically receive guideline stroke secondary prevention, the only large phase 3 trial to date (Secondary Prevention of Small Subcortical Strokes [SPS3]) that focused on lacunar stroke found that long-term use of dual antiplatelet agents was hazardous^5^ and intensive blood pressure lowering did not reduce stroke or cognitive decline.^6^

Most lacunar ischemic strokes and cSVD are thought to result from an intrinsic perforating arteriolar disorder,^2^ with dysfunction of the small vessel endothelium affecting blood supply to subcortical tissues.^7^ Drugs that stabilize endothelial function might prevent the long-term clinical, cognitive, and functional consequences of cSVD.^8,9^

Isosorbide mononitrate (ISMN), a nitric oxide (NO) donor, augments the NO-cyclic guanosine monophosphate phosphodiesterase PDE5-inhibitor pathway.^8^ Cilostazol, a PDE3 inhibitor, augments the prostacyclin-cyclic adenosine monophosphate pathway.^8,10^ Endothelial function depends on both pathways; therefore, both ISMN and cilostazol could improve vascular endothelial function. These agents are licensed for the treatment of vascular diseases, have known safety profiles, and have no direct interactions of concern in routine use.^8^ Hence, both can be tested efficiently in a 2 × 2 factorial trial.

To inform the design of large phase 3 trials, the Lacunar Intervention Trial-2 (LACI-2) aimed to evaluate trial feasibility, retention, and adherence to ISMN and cilostazol as well as their safety, tolerability, and effects on common clinical outcomes in patients with lacunar ischemic stroke.

## Methods

The UK Health Research Authority granted ethics and research and development approvals for the LACI-2 randomized clinical trial. Written informed consent was obtained from all participants prior to enrollment. We followed good clinical practice guidelines and the Consolidated Standards of Reporting Trials (CONSORT) reporting guideline.

### Trial Design and Setting

This investigator-led, randomized, open-label, blinded end-point, 2 × 2 factorial trial was conducted at 26 stroke specialist hospitals in the UK. We randomized patients between February 5, 2018, and May 31, 2021. The trial was registered with ISRCTN (ISRCTN14911850), EudraCT (2016-002277-35), and ClinicalTrials.gov (NCT03451591). The LACI-2 protocol (Supplement 1),^9^ baseline data, and statistical analysis plan (Supplement 1)^11,12^ were published previously. Oversight details are provided in the eMethods in Supplement 2.^9^

### Participants

We included patients aged older than 30 years with clinical lacunar ischemic stroke syndrome (eg, pure motor hemiparesis, pure sensory stroke, ataxic hemiparesis, sensorimotor stroke, or dysarthria-clumsy hand syndrome) and brain computed tomography (CT) or magnetic resonance imaging (MRI) showing either a visible relevant small subcortical (ie, lacunar) infarct or no alternative finding to account for the symptoms (no cortical infarct, hemorrhage, or mimic). We set no time limit between stroke and recruitment (detailed in Wardlaw^9^), mainly because we aimed to avoid the period of use of dual antiplatelet agents in early secondary prevention; because lacunar ischemic stroke indicates cSVD, which is a chronic disorder; because recurrent stroke occurs late^13^; and because we aimed to focus on long-term outcomes. Exclusion criteria were other active brain disease, kidney or hepatic impairment, dependence, and lack of capacity.^9^ Central blinded reading of diagnostic CT and MRI brain imaging was performed to assess for the index stroke lesion, white matter hyperintensities, lacunes, old infarcts or old hemorrhages, brain atrophy, and incidental pathology (all on CT and MRI)^14^ as well as microbleeds and perivascular spaces (on MRI).^15^

### Randomization and Masking

We used a 2 × 2 factorial design since both ISMN and cilostazol have relevant, potentially complementary, modes of action and no known adverse interactions, and there is no specific treatment for lacunar stroke^3^ to enable other trial designs (such as noninferiority).

We randomized participants to treatment with vs without ISMN and with vs without cilostazol (each in a 1:1 ratio), using a secure internet-based system (University of Nottingham Stroke Trials Unit). Participants with indications for, or contraindications to, one study drug could be randomized to the other drug. To ensure well-balanced treatment groups for important prognostic factors (details in Wardlaw^9^), we minimized on age, sex, stroke impairment (National Institutes of Health Stroke Scale [NIHSS] score), dependence (modified Rankin Scale [mRS] score), systolic blood pressure (≤ or >140 mm Hg), smoking status, time after stroke, and years of education. The study drugs were open label. Neither participants nor clinicians at sites were masked. However, central follow-up staff, central image assessors, statisticians, and the LACI-2 Trial Steering Committee were all masked.

### Interventions

Participants were allocated to ISMN (40-60 mg/d; typical UK hospital formulations), cilostazol (200 mg/d), ISMN-cilostazol (40-60 and 200 mg/d, respectively), or no study drug, dispensed by site hospital pharmacies. Participants started their allocated drug(s) the day after randomization at a low dose, escalating to the full or maximum tolerated dose by 4 weeks, as developed in the LACI-1 trial.^9,16^ All participants continued their usual prescribed medications, including guideline-based stroke prevention (antiplatelet [usually clopidogrel], antihypertensive, and lipid-lowering agents) and lifestyle advice (smoking cessation, diet, exercise, and sleep).

### Outcomes

The primary outcome was feasibility of recruitment and retention (defined as >95% of randomized patients retained at 1 year). Secondary outcomes included safety (primarily death^11^), efficacy (primarily the composite outcome of recurrent stroke or transient ischemic attack [TIA], myocardial infarction [MI], any cognitive impairment, dependence, and death^9^), adherence or tolerability to trial treatment (defined as 75% of patients taking ≥50% of the trial dose up to 1 year), and data on symptoms, vascular events, cognitive impairment, dependence (mRS score >2), death, mood, quality of life (QOL), bleeding, and falls for hypothesis generation and planning for future trials.

At 12 months, central masked staff assessed the following: recurrent vascular events, mRS score, and cognitive impairment, using the telephone Montreal Cognitive Assessment (tMoCA) and the Telephone Interview of Cognitive Status (TICS); mood, using the Zung score; QOL, using the 5-level EuroQol-5D visual analog score; and the Stroke Impact Scale (SIS) to assess multiple domains of participant-reported physical or cognitive function, dependence, and QOL.^9^ We calculated a 7-level ordinal cognitive outcome status by operationalizing the *Diagnostic and Statistical Manual of Mental Disorders, Fifth Edition*,^17^ using tMoCA and TICS subscores.^9^

Site staff obtained brain MRI, blood pressure, and Trail Making Test Part B results at 12 months. Central masked staff assessed MRI for new infarcts, cSVD lesions, and brain atrophy.

### Sample Size

The sample size was set based on the safety outcome (death).^9^ Assuming that all-cause death would be 2% per year, the upper 95% CI of 2% would be 4% in 400 patients. Hence, the trial would stop if all-cause deaths, including fatal hemorrhage, exceeded 4% with the study drugs vs without them.^9^

### Statistical Analysis

We performed statistical analyses according to the published statistical analysis plan (Supplement 1).^11,12^ All analyses were intention to treat per randomization allocation. We did not impute missing data. We compared treatment with vs without ISMN, with vs without cilostazol, and with ISMN-cilostazol vs no study drug. Participants who died had a score worse than any living participant score assigned to maintain power and prevent missing any “kill or cure” effect.^18^ To assess for dropout bias, we compared participants with vs without mRS or cognition (tMoCA) data at 1 year. We did not adjust for multiple testing, since safety or efficacy outcomes were hypothesis generating.

Data are reported as the number (percentage), median (IQR), or mean (SD). Analyses used binary logistic regression (presented as adjusted odds ratios [aORs]), Cox proportional hazards regression (adjusted hazard ratios [aHRs]), ordinal logistic regression (aORs), or multiple linear regression (adjusted mean difference [aMDs]) and are presented with 95% CIs. Analyses were adjusted for minimization variables (baseline age, sex, NIHSS, mRS, systolic blood pressure, smoking status, time after stroke, and years of education); cognitive outcomes were additionally adjusted for baseline MoCA scores.

The primary outcome (feasibility) was depicted graphically and numerically. The safety outcome (death) at 12 months was analyzed using binary logistic regression. The efficacy outcome (composite of stroke or TIA, MI, death, any vs no cognitive impairment on 7-level score, dependence, and death) at 12 months was analyzed using Cox proportional hazards regression. The composite required patients with data for all included variables. We assessed individual recurrent stroke and functional and cognitive outcomes using all available data.

We used the Wei-Lachin test^19^ to assess the following: (1) global clinical outcomes, using recurrent ordinal stroke, ordinal MI, ordinal 7-level cognition, ordinal mRS score, QOL (full health status utility value of the EuroQol-5D), Zung full-scale depression score, and binary status of alive or dead; and (2) global SIS, using individual SIS domain scores. Both are reported as the Mann-Whitney difference (MWD). Ordinal stroke or MI was the occurrence of stroke or MI modified using the mRS to determine the severity of the event.^19^

Sensitivity analyses assessed all participants, including those with missing data for required variables (composite) and prespecified subgroups (minimization variables, hypertension, diabetes, prior stroke or TIA, index infarct on imaging, white matter hyperintensities, or cSVD score) adjusted for cilostazol in ISMN models and for ISMN in cilostazol models.

Throughout the analysis, the threshold for statistical significance was a 2-tailed *P* value of less than 5%. Data were analyzed using SAS, version 9.4 (SAS Institute Inc). Data analysis was performed on August 12, 2022.

## Results

### Study Participants

**Figure 1.**
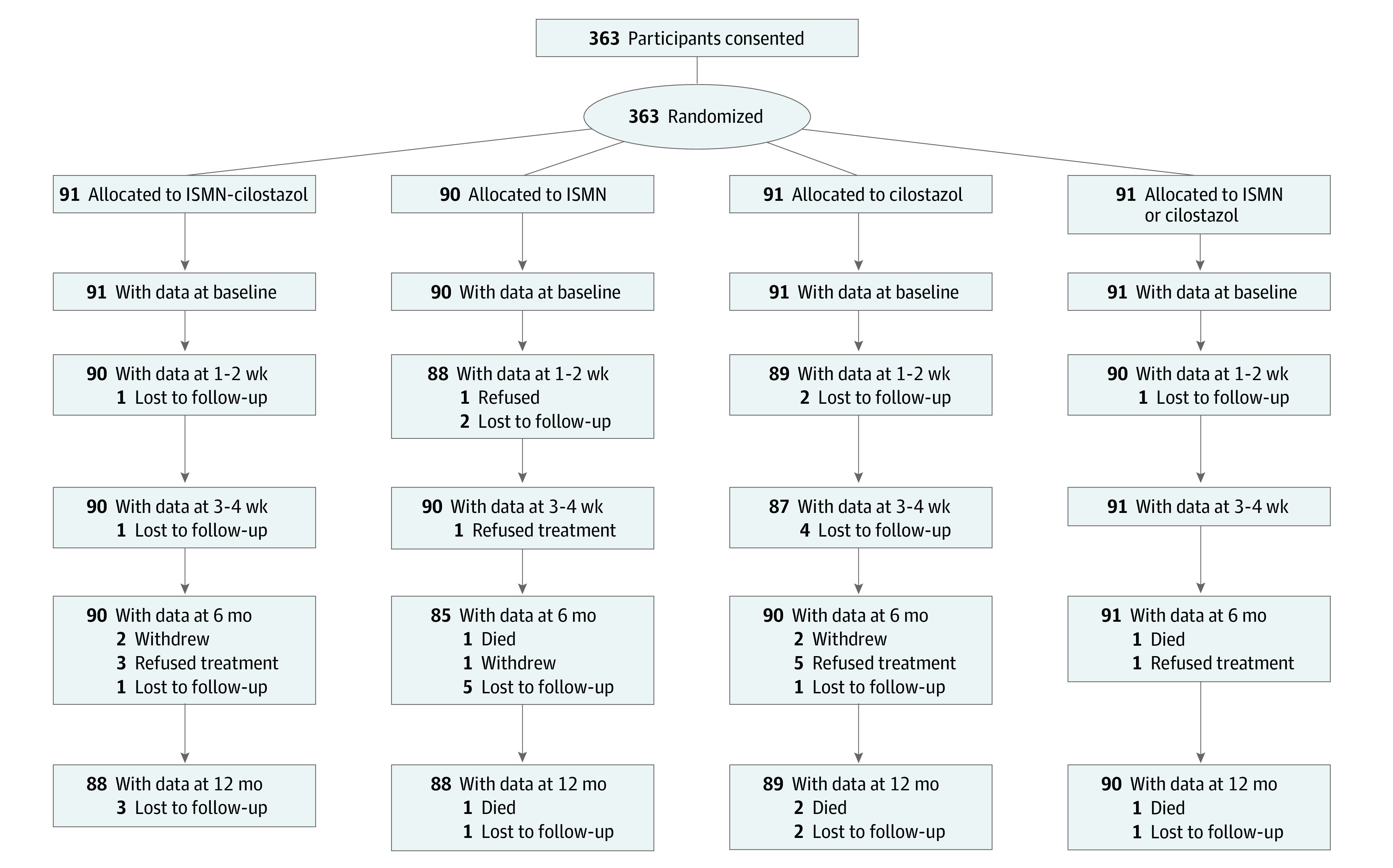
Study Flow Diagram ISMN indicates isosorbide mononitrate.

Of the 400 participants planned for this randomized clinical trial, 363 (90.8%) were recruited in 24 active months (eFigure 1 in Supplement 2). Patient characteristics were well balanced at baseline and were typical of lacunar stroke (Table 1).^11,12^ Patients had a median age of 64 (IQR, 56.0-72.0) years and a median NIHSS score of 0 (IQR, 0-2.0). There were 251 men (69.1%) and 112 women (30.9%). A total of 267 patients (73.6%) had hypertension; few had large artery atheroma (carotid stenosis ≥50%: 9 [2.9%]) or embolic sources (atrial fibrillation: 5 [1.4%]).^13,20^ The median time between stroke and randomization was 79 (IQR, 27.0-244.0) days. In terms of baseline brain imaging among the 363 patients, 100 (27.5%) had CT data and 263 (72.5%) had MRI data. A total of 320 patients (88.2%) had a visible index lacunar infarct, 42 (11.6%) had no index infarct but had other cSVD lesions, and 1 (0.3%) had a normal scan.^12^ White matter hyperintensities were moderate in 143 patients (39.4%) and severe in 75 (20.7%). Isosorbide mononitrate was contraindicated in 23 patients (6.3%) and cilostazol in 45 (12.4%). Randomization allocated patients to treatment with vs without ISMN (n = 181 vs 182), with vs without cilostazol (n = 182 vs 181), with ISMN alone (n = 90), with cilostazol alone (n = 91) with ISMN-cilostazol (n = 91), and with no study drug (n = 91) (Figure 1 and Table 1). We obtained 1-year follow-up data for 358 participants (98.6%), thereby exceeding the primary feasibility target of 95%.

**Table 1.** Baseline Characteristics by Treatment Allocation to Isosorbide Mononitrate (ISMN), Cilostazol, ISMN-Cilostazol, or No Study Drug

Characteristic	All patients (N = 363), No. (%)	ISMN		Cilostazol		All groups			
		With (n = 181)	Without (n = 182)	With (n = 182)	Without (n = 181)	ISMN-cilostazol (n = 91)	ISMN (n = 90)	Cilostazol (n = 91)	None (n = 91)
Age, y, median (IQR)^a^	64.0 (56.0-72.0)	64.0 (56.0-72.0)	64.0 (57.0-73.0)	63.5 (56.0-72.0)	65.0 (57.0-72.0)	63.0 (56.0-72.0)	65.0 (56.0-72.0)	64.0 (57.0-73.0)	64.0 (57.0-74.0)
Sex, No. (%)									
Women	112 (30.9)	54 (29.8)	58 (31.9)	55 (30.2)	57 (31.5)	27 (29.7)	27 (30.0)	28 (30.8)	30 (33.0)
Men	251 (69.1)	127 (70.2)	124 (68.1)	127 (69.8)	124 (68.5)	64 (70.3)	63 (70.0)	63 (69.2)	61 (67.0)
mRS score >1, mean (SD)	85 (23.4)	40 (22.1)	45 (24.7)	43 (23.6)	42 (23.2)	21 (23.1)	19 (21.1)	22 (24.2)	23 (25.3)
Onset to randomization, d, median (IQR)^a^	79.0 (27.0-244.0)	83.0 (34.0-251.0)	76.0 (23.0-244.0)	83.0 (37.0-238.0)	75.0 (20.0-251.0)	100.0 (41.0-252.0)	74.5 (21.0-251.0)	75.0 (28.0-238.0)	77.0 (17.0-256.0)
Completed education, y, median (IQR)	16.0 (15.0-18.0)	16.0 (15.0-18.0)	16.0 (15.0-18.0)	16.0 (15.0-18.0)	16.0 (15.0-18.0)	16.0 (15.0-18.0)	16.0 (15.0-17.0)	16.0 (15.0-18.0)	16.0 (15.0-18.0)
Current smoker, No. (%)	67 (18.5)	34 (18.8)	33 (18.1)	34 (18.7)	33 (18.2)	18 (19.8)	16 (17.8)	16 (17.6)	17 (18.7)
Comorbidity, No. (%)									
Hypertension	267 (73.6)	135 (74.6)	132 (72.5)	134 (73.6)	133 (73.5)	67 (73.6)	68 (75.6)	67 (73.6)	65 (71.4)
Hyperlipidemia	281 (77.4)	132 (72.9)	149 (81.9)	147 (80.8)	134 (74.0)	67 (73.6)	65 (72.2)	80 (87.9)	69 (75.8)
Diabetes	80 (22.0)	38 (21.0)	42 (23.1)	40 (22.0)	40 (22.1)	18 (19.8)	20 (22.2)	22 (24.2)	20 (22.0)
Atrial fibrillation	5 (1.4)	3 (1.7)	2 (1.1)	1 (0.5)	4 (2.2)	1 (1.1)	2 (2.2)	0	2 (2.2)
Carotid stenosis >50% (n = 315)	9 (2.9)	6 (2.8)	3 (1.9)	3 (2.0)	6 (3.7)	2 (2.5)	4 (5.0)	1 (1.4)	2 (2.5)
Previous stroke	25 (6.9)	9 (5.0)	16 (8.8)	14 (7.7)	11 (6.1)	5 (5.5)	4 (4.4)	9 (9.9)	7 (7.7)
Prior transient ischemic attack	29 (8.0)	10 (5.5)	19 (10.4)	12 (6.6)	17 (9.4)	5 (5.5)	5 (5.6)	7 (7.7)	12 (13.2)
Medication use, No. (%)									
Antiplatelets	352 (97.0)	176 (97.2)	176 (96.7)	177 (97.3)	175 (96.7)	88 (96.7)	88 (97.8)	89 (97.8)	87 (95.6)
Antihypertensives	277 (76.3)	144 (79.6)	133 (73.1)	135 (74.2)	142 (78.5)	70 (76.9)	74 (82.2)	65 (71.4)	68 (74.7)
Statins	338 (93.1)	166 (91.7)	172 (94.5)	173 (95.1)	165 (91.2)	85 (93.4)	81 (90.0)	88 (96.7)	84 (92.3)
Blood pressure, mm Hg, median (IQR)^a^									
Systolic	143.0 (130-157)	143.0 (132-157)	142.0 (128-158)	142.0 (131-157)	144.0 (130-158)	143.0 (133-156)	144.0 (131-159)	142.0 (130-159)	143.0 (126-156)
Diastolic	83.0 (75.0-90.0)	84.0 (76.0-91.0)	82.0 (75.0-89.0)	83.0 (76.0-92.0)	82.0 (74.0-89.0)	84.0 (76.0-92.0)	83.0 (76.0-90.0)	83.0 (76.0-92.0)	81.0 (72.0-89.0)
Assessment, median (IQR)									
NIHSS score out of 42	0 (0-2.0)	0 (0-2.0)	0 (0-2.0)	0 (0-1.0)	1.0 (0-2.0)	0 (0-1.0)	1.0 (0-2.0)	0 (0-1.0)	0 (0-2.0)
Cognition MoCA score out of a total of 30 (n = 362)	26.0 (23.0-28.0)	26.0 (23.0-28.0)	26.0 (23.0-28.0)	26.0 (24.0-28.0)	26.0 (23.0-28.0)	26.0 (24.0-28.0)	26.0 (23.0-28.0)	26.0 (23.0-28.0)	27.0 (23.0-28.0)
Trail Making Test Part B score (n = 359)									
Time, s	110.0 (75.0-170)	109.0 (74.0-161.0)	110.5 (78.0-173.0)	110.0 (74.0-162.5)	110.0 (77.0-177.0)	100.0 (71.0-156.0)	110.0 (75.0-171.0)	116.5 (77.0-170.0)	108.5 (79.0-179.0)
Points	25.0 (23.0-25.0)	25.0 (23.0-25.0)	25.0 (23.0-25.0)	25.0 (23.0-25.0)	25.0 (23.0-25.0)	25.0 (23.0-25.0)	24.0 (23.0-25.0)	25.0 (22.0-25.0)	25.0 (23.0-25.0)
Imaging results, No. (%)									
Index infarct present	320 (88.2)	160 (88.4)	160 (87.9)	163 (89.6)	157 (86.7)	82 (90.1)	78 (86.7)	81 (89.0)	79 (86.8)
Fazekas score of white matter hyperintensity									
0-2	124 (34.2)	64 (35.4)	60 (33.0)	60 (33.0)	64 (35.4)	30 (33.0)	34 (37.8)	30 (33.0)	30 (33.0)
3-4	143 (39.4)	73 (40.3)	70 (38.5)	76 (41.8)	67 (37.0)	41 (45.1)	32 (35.6)	35 (38.5)	35 (38.5)
5-6	75 (20.7)	32 (17.7)	43 (23.6)	38 (20.9)	37 (20.4)	17 (18.7)	15 (16.7)	21 (23.1)	22 (24.2)
Contraindication, No. (%)									
ISMN	23 (6.3)	0	23 (12.6)	8 (4.4)	15 (8.3)	0	0	8 (8.8)	15 (16.5)
Cilostazol	45 (12.4)	25 (13.8)	20 (11.0)	1 (0.5)^b^	44 (24.3)	1 (1.1)^b^	24 (26.7)	0 (0.0)	20 (22.0)

Abbreviations: ISMN, isosorbide mononitrate; MoCA, Montreal Cognitive Assessment; mRS, modified Rankin Scale; NIHSS, National Institutes of Health Stroke Scale.

^a^ Minimization variables included age (≤ vs >70 years), onset to randomization (≤ vs >100 days), highest level of education attained, systolic blood pressure, smoker status, stroke severity (NIHSS), and dependence from the stroke (mRS).

^b^ One patient initially randomized to ISMN-cilostazol was noted to have an electrocardiogram contraindication to cilostazol (before receiving any drug) and thus only received a prescription for ISMN; this patient was retained in the cilostazol/ISMN-cilostazol groups for intention-to-treat analysis.

### Drug Tolerance, Safety, and Symptoms

The number of patients taking at least half of the study drug was 257 of 272 (94.5%) overall. eTable 1 in Supplement 2 presents the proportions of patients taking the individual study drugs at 12 months.

There were few serious adverse events (eTables 2 and 3 in Supplement 2). Only 2 were likely attributable to the study drugs (1 each to ISMN and cilostazol).

Nine symptoms were assessed, including headache, palpitations, dizziness, loose stools, nausea, bleeding, dyspepsia, bruising, and falls (eTable 4 in Supplement 2). Headache increased with ISMN (aOR, 1.89 [95% CI, 1.23 to 2.92]; *P* = .004) and loose stools increased with cilostazol (aOR, 2.48 [95% CI, 1.63 to 3.79]; *P* < .001). Both headache (aOR, 2.03 [95% CI, 1.10 to 3.74]; *P* = .02) and loose stools (aOR, 2.13 [95% CI, 1.18 to 3.86]; *P* = .01) increased with ISMN-cilostazol but did not affect daily activities. Other symptoms were not increased.

### Clinical Outcomes

At 12 months, adverse events included death (4 of 358 [1.1%]; so below the safety limit), hemorrhage (all systemic; 3 [1.7%] with ISMN vs 1 [0.6%] without ISMN; 3 [1.7%] with cilostazol vs 1 [0.6%] without cilostazol; and 2 [2.2%] with ISMN-cilostazol vs 0 [0%] with no drug), recurrent stroke or TIA (19 [5.3%]), and MI (4 [1.1%]). Functional status was available for 323 participants (90.2%), 47 of whom (14.6%) were dependent. The composite outcome (stroke, TIA, MI, dependence, any cognitive impairment, and death) occurred in 183 of 297 participants (61.6%) with complete data. Cognitive status was available for 307 (tMoCA), 313 (TICS-M), and 319 (verbal fluency) participants, providing a 7-level ordinal cognitive outcome for 308 participants. Of these 308 participants, 184 (59.7%) had mild or worse cognitive impairment. There was no difference in 12-month blood pressure between groups (Table 2).

**Table 2.** Clinical, Functional, Quality of Life, and Global Outcomes at 12 Months^a^

Outcome	All patients (n = 358), No. (%)	ISMN				Cilostazol				ISMN-cilostazol			
		With (n = 178)	Without (n = 180)	aOR, aHR, aMD, or MWD (95% CI)^b^	*P* value	With (n = 178)	Without (n = 180)	aOR, aHR, aMD, or MWD (95% CI)	*P* value	With (n = 89)	Without (n = 91)	aOR, aHR, aMD, or MWD (95% CI)	*P* value
Composite, No. (%) (n = 297)^c^	183 (61.6)	80 (55.2)	103 (67.8)	0.80 (0.59 to 1.09)	.16	84 (57.1)	99 (66.0)	0.77 (0.57 to 1.05)	.10	36 (48.6)	55 (69.6)	0.58 (0.36 to 0.92)	.02
Stroke or transient ischemic attack^d^	11 (3.7)	1 (0.7)	10 (6.6)	0.08 (0.01 to 0.67)	.02	7 (4.8)	4 (2.7)	2.02 (0.51 to 7.90)	.31	0	3 (3.8)	NA	NA
Myocardial infarction^d^	3 (1.0)	2 (1.4)	1 (0.7)	1.04 (0.04 to 26.22)	.98	2 (1.4)	1 (0.7)	0.88 (0.04 to 19.34)	.94	1 (1.4)	0	NA	NA
Cognitive impairment^d^	175 (58.9)	78 (53.8)	97 (63.8)	0.66 (0.39 to 1.14)	.13	81 (55.1)	94 (62.7)	0.71 (0.41 to 1.21)	.21	35 (47.3)	51 (64.6)	0.44 (0.19 to 0.99)	.046
Dependence, mRS >2^d^	39 (13.1)	16 (11.0)	23 (15.1)	0.74 (0.34 to 1.63)	.46	13 (8.8)	26 (17.3)	0.31 (0.14 to 0.72)	.006	4 (5.4)	14 (17.7)	0.14 (0.03 to 0.59)	.008
Death	4 (1.3)	1 (0.7)	3 (2.0)	0.32 (0.02 to 5.61)	.44	2 (1.4)	2 (1.3)	0.90 (0.08 to 10.26)	.93	0	1 (1.3)	NA	NA
Sensitivity: composite, No. (%) (n = 317)^c^	203 (64.0)	88 (57.5)	115 (70.1)	0.80 (0.60 to 1.08)	.14	94 (59.9)	109 (68.1)	0.80 (0.60 to 1.07)	.14	40 (51.3)	61 (71.8)	0.61 (0.39 to 0.96)	.03
Clinical parameter													
Blood pressure, mm Hg, mean (SD) (n = 164)^e^													
Systolic	142.3 (18.8)	143.0 (19.4)	141.5 (18.2)	1.83 (−3.67 to 7.32)	.51	143.5 (17.8)	141.0 (19.7)	1.14 (−4.22 to 6.51)	.68	142.5 (18.1)	138.5 (18.5)	2.56 (−5.35 to 10.46)	.53
Diastolic	82.4 (10.3)	82.4 (10.5)	82.5 (10.2)	0.45 (−2.61 to 3.52)	.77	82.7 (9.0)	82.2 (11.6)	0.37 (−2.63 to 3.36)	.81	82.0 (9.6)	81.6 (11.9)	0.38 (−4.18 to 4.94)	.87
Recurrent stroke or TIA, mean (SD)^d^	19 (5.3)	4 (2.2)	15 (8.3)	0.23 (0.07 to 0.74)	.01	11 (6.2)	8 (4.4)	1.35 (0.51 to 3.57)	.55	2 (2.2)	6 (6.6)	0.21 (0.03 to 1.39)	.11
mRS score, median (IQR) (n = 323)^f^	1.0 (1.0- 2.0)	1.0 (0- 2.0)	1.0 (1.0-2.0)	0.67 (0.45 to 1.02)	.06	1.0 (1.0-2.0)	1.0 (0- 2.0)	0.85 (0.56 to 1.28)	.44	1.0 (1.0-2.0)	1.0 (1.0-2.0)	0.51 (0.28 to 0.93)	.03
Dependence mRS score >2, No. (%) (n = 3358)^d^	47 (14.6)	19 (12.0)	28 (17.0)	0.65 (0.32 to 1.33)	.24	18 (11.5)	29 (17.5)	0.46 (0.22 to 0.95)	.04	7 (8.9)	17 (19.5)	0.27 (0.09 to 0.82)	.02
QOL, mean (SD) (n = 320)													
EuroQol-5D 5-level HSUV (of 1)^e^	0.8 (0.3)	0.8 (0.2)	0.7 (0.3)	0.06 (0.01 to 0.11)	.03	0.8 (0.2)	0.8 (0.3)	0.04 (−0.01 to 0.09)	.15	0.8 (0.2)	0.7 (0.3)	0.10 (0.03 to 0.17)	.005
EuroQol VAS (of 100)^e^	76.2 (22.1)	78.8 (19.8)	73.7 (23.8)	4.66 (0.22 to 9.11)	.04	77.8 (21.3)	74.6 (22.7)	3.82 (−0.64 to 8.28)	.09	79.7 (18.8)	71.8 (24.1)	9.00 (3.05 to 14.95)	.003
Global analysis, median (IQR)													
Clinical outcomes (n = 308)^g^^,^^h^	NA	NA	NA	−0.08 (−0.15 to −0.02)	.005	NA	NA	−0.03 (−0.09 to 0.02)	.25	NA	NA	−0.12 (−0.20 to −0.04)	.004
Stroke Impact Scale outcomes only (n = 286)^h^	NA	NA	NA	−0.14 (−0.24 to −0.04)	.005	NA	NA	−0.03 (−0.13 to 0.07)	.58	NA	NA	−0.17 (−0.31 to −0.03)	.02

Abbreviations: aHR, adjusted hazard ratio; aMD, adjusted mean difference; aOR, adjusted odds ratio; HSUV, health status utility value; ISMN, isosorbide mononitrate; MLR, multiple linear regression; mRS, modified Rankin Scale; MWD, Mann-Whitney difference; NA, not applicable; NIHSS, National Institutes of Health Stroke Scale; OLR, ordinal logistic regression; VAS, visual analog scale.

^a^ Analyses were performed using binary logistic regression, OLR, or MLR with adjustment for age, sex, time from stroke onset to randomization, years of education, smoking status, and baseline mRS score (dependence), stroke severity (NIHSS), and systolic blood pressure. For ordinal scales with more than 7 levels (central limit theorem/large sample), the mean (SD) and MLR were used instead of the median (IQR) and OLR. Composite includes only patients with complete data for the required outcomes; sensitivity includes all patients, including those with missing data.

^b^ The MWD is calculated as the probability of having a good outcome on control minus the probability of having a good outcome on treatment. If the probability of having a good outcome on treatment is higher than the control probability, then the result will be below 0.

^c^ Cox proportional hazard time to event (aHR).

^d^ Binary logistic regression (aOR).

^e^ Multiple linear regression (aMD).

^f^ Ordinal logistic regression (aOR).

^g^ Includes recurrent ordinal stroke, ordinal myocardial infarction, 7-level cognition, ordinal dependence (mRS score), quality of life using the full Health Status Utility Score of the EuroQol-5D, the Zung full-scale depression score, and binary status of alive or dead. Analyzed using the Wei-Lachin test, not adjusted.

^h^ Wei-Lachin test (MWD).

### Missing Data

The main reasons for missing functional or cognitive data were patient withdrawal (12 [10.0%]) or loss to follow-up (13 [10.9%]), with no evidence of more missing data by allocated drug. Patients without vs with 12-month cognitive data were more often male, were more dependent, and had lower tMoCA scores at baseline, but without differences by allocated drug (eTables 5B and 6A-C in Supplement 2).

### ISMN Treatment

**Figure 2.**
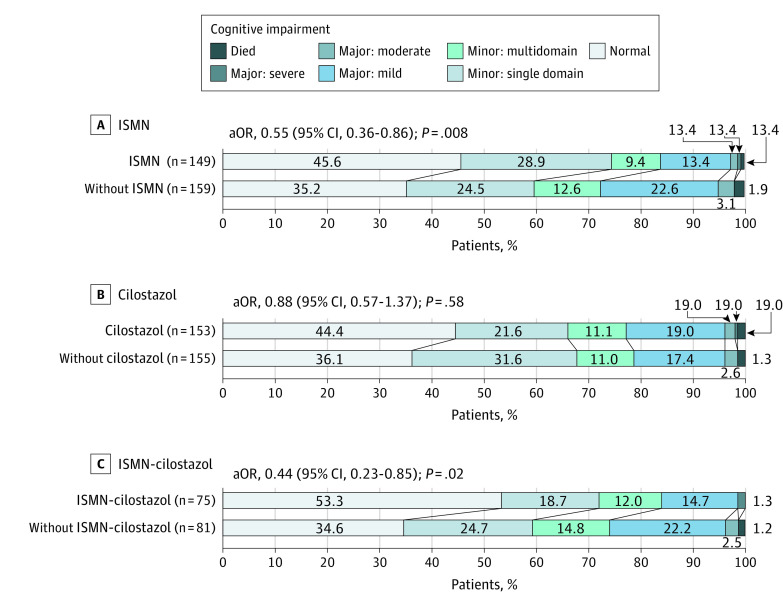
Cognition at 12 Months Assessed Using 7-Level Ordinal Adjusted Analysis aOR indicates adjusted odds ratio; ISMN, isosorbide mononitrate.

Compared with no ISMN treatment, ISMN did not reduce the composite outcome (80 of 145 [55.2%] with ISMN vs 103 of 152 [67.8%] without; aHR, 0.80 [95% CI, 0.59 to 1.09]; *P* = .16). However, ISMN reduced recurrent stroke or TIA (4 of 178 [2.2%] with ISMN vs 15 of 180 [8.3%] without; aOR, 0.23 [95% CI, 0.07 to 0.74]; *P* = .01), improved QOL (aMD, 0.06 [95% CI, 0.01 to 0.11]; *P* = .03), reduced global SIS (MWD, −0.14 [95% CI, −0.15 to −0.02]; *P* = .005), reduced global clinical outcomes (MWD, −0.09 [−0.50 to −0.03]; *P* = .004), tended to reduce dependence (Table 2 and eFigure 2 in Supplement 2), and reduced cognitive impairment (7-level ordinal aOR, 0.55 [95% CI, 0.36 to 0.86]; *P* = .008; Figure 2 and Table 3). The absolute reduction in participants with any cognitive impairment was 10.4% (54.4% with ISMN and 64.8% without ISMN).

**Table 3.** Cognitive and Mood Outcomes at 12 Months

Outcome	All patients (n = 358), No. (%)	ISMN				Cilostazol				ISMN-cilostazol			
		With (n = 178)	Without (n = 180)	aOR, aHR, or aMD (95% CI)	*P* value	With (n = 178)	Without (n = 180)	aOR, aHR, aMD (95% CI)	*P* value	With (n = 89)	Without (n = 91)	aOR, aHR, or aMD (95% CI)	*P* value
Ordinal 7-level cognition, No. (%) (n = 308)^a^													
Normal	124 (40.3)	68 (45.6)	56 (35.2)	0.55 (0.36 to 0.86)	0.008	68 (44.4)	56 (36.1)	0.88 (0.57 to 1.37)	0.58	40 (53.3)	28 (34.6)	0.44 (0.23 to 0.85)	0.02
Minor													
Single domain	82 (26.6)	43 (28.9)	39 (24.5)			33 (21.6)	49 (31.6)			14 (18.7)	20 (24.7)		
Multidomain	34 (11.0)	14 (9.4)	20 (12.6)			17 (11.1)	17 (11.0)			9 (12.0)	12 (14.8)		
Major													
Mild	56 (18.2)	20 (13.4)	36 (22.6)			29 (19.0)	27 (17.4)			11 (14.7)	18 (22.2)		
Moderate	7 (2.3)	2 (1.3)	5 (3.1)			3 (2.0)	4 (2.6)			0	2 (2.5)		
Severe	1 (0.3)	1 (0.7)	0			1 (0.7)	0			1 (1.3)	0		
Died	4 (1.3)	1 (0.7)	3 (1.9)			2 (1.3)	2 (1.3)			0	1 (1.2)		
Individual cognition, mean (SD)													
tMoCA score out of 22 (n = 307)^b^	18.4 (3.5)	18.8 (3.1)	18.0 (3.9)	0.62 (−0.12 to 1.36)	0.10	18.6 (3.6)	18.1 (3.5)	0.37 (−0.37 to 1.11)	0.33	19.3 (2.6)	18.0 (3.6)	1.14 (0.24 to 2.04)	0.01
Verbal fluency (animal naming) (n = 319)^b^	19.5 (7.3)	20.0 (7.4)	19.0 (7.2)	0.75 (−0.74 to 2.25)	0.32	19.8 (7.7)	19.2 (6.9)	0.56 (−0.93 to 2.05)	0.46	19.8 (7.3)	18.2 (6.1)	1.21 (−0.65 to 3.06)	0.20
Trail Making Test Part B score (n = 156)													
Time, s^b^	123.0 (64.9)	114.9 (57.2)	131.2 (71.3)	−13.39 (−32.00 to 5.21)	0.16	120.5 (57.2)	125.5 (72.2)	−2.98 (−21.45 to 15.50)	0.75	112.1 (48.0)	134.0 (78.8)	−22.36 (−47.56 to 2.84)	0.08
Points^b^	22.8 (5.3)	22.4 (5.7)	23.2 (5.0)	−1.00 (−2.65 to 0.65)	0.23	22.3 (6.5)	23.3 (3.8)	−0.86 (−2.50 to 0.78)	0.30	21.9 (6.6)	23.9 (2.6)	−1.62 (−3.75 to 0.51)	0.14
Mood, mean (SD)													
Zung depression scale score out of 102.5 (n = 317)^b^	50.0 (17.3)	48.5 (16.3)	51.3 (18.1)	−2.23 (−5.70 to 1.24)	0.21	48.4 (16.8)	51.4 (17.7)	−3.34 (−6.81 to 0.14)	0.06	47.6 (15.9)	53.2 (18.4)	−5.98 (−10.77 to −1.20)	0.01

Abbreviations: aHR, adjusted hazard ratio; aMD, adjusted mean difference; aOR, adjusted odds ratio; MLR, multiple linear regression; mRS, modified Rankin Scale; NIHSS, National Institutes of Health Stroke Scale; OLR, ordinal logistic regression; tMoCA, telephone Montreal Cognitive Assessment.

^a^ Analyses were performed using OLR with output as aMD; all adjusted for age, sex, time from stroke onset to randomization, years of education, smoking status, baseline mRS (dependence), stroke severity (NIHSS), systolic blood pressure, and baseline tMoCA.

^b^ Analyses were performed using MLR with output as aMD; all were adjusted as for OLR. For ordinal scales with more than 7 levels (central limit theorem/large sample), the mean (SD) and MLR were used instead of the median (IQR) and OLR.

### Cilostazol Treatment

Cilostazol did not reduce the composite outcome, recurrent stroke or TIA, or improve QOL, global SIS, or global clinical outcome, but it reduced dependence (mRS score of 3-6: 13 of 147 [8.8%] with cilostazol vs 26 of 150 [17.3%] without; aOR, 0.31 [95% CI, 0.14 to 0.72]; *P* = .006; Table 2). Cilostazol did not reduce cognitive impairment but tended to improve mood (Zung depression scale score: aMD, −3.34 [95% CI, −6.81 to 0.14]; *P* = .06; Table 3).

### ISMN-Cilostazol Treatment

Combination ISMN-cilostazol reduced the composite outcome (aHR, 0.58 [95% CI, 0.36 to 0.92]; *P* = .02), dependence (ordinal shift analysis: aOR, 0.51 [95% CI, 0.28 to 0.93]; *P* = .03; eFigure 2 in Supplement 2; and mRS score >2: 4 of 74 [2.7%] vs 14 of 79 [17.7%]; aOR, 0.14 [95% CI, 0.03 to 0.59]; *P* = .008), global clinical outcomes (MWD, −0.12 [95% CI, −0.20 to −0.04]; *P* = .004), improved QOL (aMD, 0.10 [95% CI, 0.03 to 0.17]; *P* = .005), and global SIS (MWD, −0.17 [95% CI, −0.31 to −0.03]; *P* = .02), but not recurrent stroke (Table 2). Combination ISMN-cilostazol reduced cognitive impairment (7-level ordinal aOR, 0.44 [95% CI, 0.23 to 0.85]; *P* = .02), improved tMoCA scores (aMD, 1.14 [95% CI, 0.24 to 2.04]; *P* = .01), tended to better Trail Making Test Part B scores, and reduced low mood on the Zung depression scale (aMD, −5.98 [95% CI, −10.77 to −1.20]; *P* = .01); the absolute reduction in participants with any cognitive impairment was 18.7% (46.7% with ISMN-cilostazol and 65.4% with neither drug; Table 3).

### Sensitivity

There were no subgroup interactions (composite outcome; eFigure 3 in Supplement 2). Including interaction terms for cilostazol in ISMN models and vice versa did not alter the findings. Including patients with missing data did not change composite outcomes (n = 318; Table 2).

## Discussion

The LACI-2 randomized clinical trial confirmed the feasibility of testing drugs that stimulate the NO and prostacyclin pathways in patients with lacunar ischemic stroke.^8^ In this study, ISMN and cilostazol were well tolerated and safe, with few adverse symptoms when added to guideline stroke prevention, and may improve vascular, functional, and cognitive outcomes. If confirmed in a larger trial, these findings would be clinically meaningful. Both drugs are widely available and inexpensive.

Participants in the LACI-2 trial had characteristics typical of lacunar ischemic stroke,^13^ including being younger compared with all patients with stroke,^6^ more were men,^20^ few strokes had embolic sources,^13^ and patients had low rates of dependence and death (1.3%) but high rates of cognitive impairment (58.9%).^21,22^ These LACI-2 results suggest that vascular endothelial-stabilizing drugs might prevent cognitive impairment and dependence in cSVD, consistent with endothelial dysfunction causing cSVD.^2^

### Limitations and Strengths

This study has some limitations. Placebo was not available, although the follow-up coordinators were carefully masked to the allocated drug. During the trial, 8 follow-up coordinators worked from 2 separate centers, decreasing the likelihood of unmasking. The COVID-19 pandemic affected recruitment (4-month suspension in 2020 and a slow restart) and in-person follow-up (Trail Making Test Part B, blood pressure, and MRI results), and it may have contributed to the 10.9% of patients missing central follow-up. The comparison of ISMN-cilostazol vs no drugs was underpowered. Owing to safety concerns, we excluded patients who were dependent (mRS score >2) or lacked capacity to consent, but future trials could include these patients. While lacunar ischemic stroke affects men more than women (2:1 ratio),^20^ future trials should attempt to increase recruitment of women. To streamline data collection, we did not collect data on race and ethnicity; future trials should record these data and include different racial and ethnic populations.

The LACI-2 trial also had several strengths. The web-based randomization minimization ensured that key prognostic variables and identifying information were secure and balanced at baseline. The distribution of trial work between 2 coordinating sites helped reduce the risk of systematic bias or unmasking. The trial benefitted from the UK National Institute for Health Research Clinical Research Network infrastructure. The pragmatic approach to patient inclusion reflected clinical practice and reduced barriers to recruitment and cost (eg, there were very few nonlacunar ischemic strokes and no stroke mimics), despite not mandating MRI. This improved the generalizability of LACI-2, since MRI is not universally accessible or tolerated. We included patients with clinically definite lacunar stroke but no relevant imaging-visible infarct, since they have similar recurrent stroke and dependence rates as those with visible infarction.^23^ In fact, 88.2% of participants in LACI-2 had a relevant visible infarct with no evidence that outcomes differed by infarct presence or absence. The factorial design allowed testing of 2 drugs both individually and combined compared with guideline-based stroke prevention; the comparisons are appropriate to this design and demonstrate superiority over guideline stroke prevention in lacunar stroke.

The composite outcome (recurrent stroke, cognitive impairment, functional impairment, and death) reflected the main outcomes^24^ and concerns of patients with lacunar stroke and cSVD.^21^ Both study drugs reduced some individual outcomes but not the composite, although ISMN-cilostazol did reduce composite outcomes. These findings are consistent with the global clinical outcome and global SIS, when assessing mRS scores and cognition in different ways and across subgroups including age, white matter hyper intensity score, cSVD score, and blood pressure, although LACI-2 was not powered to identify subgroup interactions.

Both ISMN and cilostazol have been widely used for ischemic heart disease, peripheral vascular disease, or secondary prevention of atherothromboembolic stroke for years, with known, acceptable safety profiles. Despite concerns that adverse symptoms attributable to these drugs would discourage compliance, most patients remained in the trial (taking ≥50% of the allocated drug) for 1 year. The study drugs were administered in addition to guideline-based stroke secondary prevention (mostly clopidogrel in the UK) with no increased bleeding with cilostazol, consistent with findings reported in systematic reviews.^10,25^

The lack of effect of cilostazol on recurrent stroke observed here differs from meta-analyses of secondary ischemic stroke prevention,^10,25^ possibly reflecting longer treatment duration in prior trials, albeit in subgroup analyses.^10^ Data for cilostazol on cognitive impairment or functional outcomes are sparse.

To our knowledge, NO donors have not been studied previously long term in patients with lacunar stroke or cSVD. Very short-term glyceryl trinitrate did not improve outcomes in acute stroke trials (n >5500),^26^ including in lacunar stroke.^27^ Vascular NO levels are low in acute and chronic stroke,^28^ so long-term ISMN might replace inadequate NO.

In the LACI-2 trial, effects on outcomes were achieved without changes in blood pressure. Intensive vs guideline blood pressure reduction had limited effects on cognition, recurrent stroke, or cSVD lesion progression in lacunar stroke.^4,6^ Intensive blood pressure reduction reduced mild cognitive impairment in the SPRINT-MIND trial^29^ and white matter hyperintensity progression in a SPRINT-MIND substudy,^30^ but it required intensive monitoring and a treatment duration of more than 3 years, underscoring the need for other approaches in cSVD.

Results from LACI-2 demonstrate the strengths of composite and global analyses, compensating for low individual recurrent vascular events (5.3% for stroke and 1.1% for MI), and dependence rates (14.6%) that make it difficult to power cSVD trials using traditional stroke outcomes. This trial also shows the value of clinical end points, including cognition, in cSVD trials.

## Conclusions

The LACI-2 trial design was feasible in patients with lacunar stroke, and ISMN and cilostazol were tolerated and may be taken together safely, in addition to guideline stroke prevention, and may improve clinical outcomes including function, cognition, mood, and QOL. These results need confirmation in larger trials in lacunar stroke; the LACI-3 trial is in preparation. Endothelial-stabilizing drugs like ISMN and cilostazol could be tested in covert cSVD to delay cognitive decline in patients at risk of or with early-stage vascular cognitive impairment, potentially in cSVD-related intracerebral hemorrhage, and in non-cSVD stroke where cSVD features are common.

## Notes

Supplement 1.
Trial Protocol and Statistical Analysis Plan

Supplement 2.
eMethods
**eTable 1.** Adherence to Medication at Half Dose or More by Allocated Group: Isosorbide Mononitrate (ISMN), Cilostazol (Cil), and Both Drugs Together **eTable 2.** Serious Adverse Events (SAEs) by Timing and Relatedness to Allocated Treatment **eTable 3.** Serious Adverse Events (SAEs) by System **eTable 4.** Symptoms Related to Either Drug Occurring at Any Time **eTable 5.** Missing Data at 12 Months: Reasons and Distributions **eTable 6.** Comparison of Participants Without vs With Data on the Telephone Montreal Cognitive Assessment at 12 Months **eFigure 1.** Recruitment Graph **eFigure 2.** Modified Rankin Scale (mRS) at 12 Months, Ordinal Shift Distributions **eFigure 3.** Composite Outcome (Recurrent Stroke or TIA or MI, Any Cognitive Impairment, Dependency mRS >2, Death) by Subgroups **eAppendix.** Lacunar Intervention Trial-2 (LACI-2) Investigator Group
eReferences

Supplement 3.
Nonauthor Contributors

Supplement 4.
Data Sharing Statement
