# Supplementary material for: Isosorbide Mononitrate and Cilostazol Treatment in Patients With Symptomatic Cerebral Small Vessel Disease: The Lacunar Intervention Trial-2 (LACI-2) Randomized Clinical Trial
Source: JAMA Neurol. 2023 May 24;80(7):682–92. doi: 10.1001/jamaneurol.2023.1526 (PMC10209826; doi:10.1001/jamaneurol.2023.1526)
Supplement: Supplement 1. — Trial Protocol and Statistical Analysis Plan [file jamaneurol-e231526-s001.pdf]

Study Protocol

LACunar Intervention (LACI-2) Trial-2: Assessment of safety and efficacy of cilostazol and isosorbide mononitrate to prevent recurrent lacunar stroke and progression of cerebral small vessel disease.

Co-sponsors	University of Edinburgh & NHS Lothian<br>ACCORD<br>The Queen's Medical Research Institute<br>47 Little France Crescent<br>Edinburgh<br>EH16 4TJ
Funder	British Heart Foundation
Funding Reference Number	CS/15/5/31475
Chief Investigator	Professor Joanna M Wardlaw
EudraCT Number	2016-002277-35
REC Number	17/EM/0077
ISRCTN Number	14911850
Version Number and Date	Version 7.0 14Oct 2020

COORDINATING CENTRE

**Chief Investigator**<br><br>Professor Joanna M Wardlaw<br>Neuroimaging Sciences<br>University of Edinburgh<br>Centre for Clinical Brain Sciences (CCBS)<br>Chancellor's Building<br>49 Little France Crescent<br>Edinburgh EH16 4SB<br><br>Tel: +44 (0)131 465 9599<br>Fax: Not available<br>Email: Joanna.Wardlaw@ed.ac.uk	**Co-sponsor Representative**<br><br>Dr Fiach O'Mahony<br>Clinical Research Facilitator<br>Research Governance & QA Office<br>University of Edinburgh<br>The Queen's Medical Research Institute<br>47 Little France Crescent<br>Edinburgh, EH16 4TJ<br><br>Tel: +44 (0)131 242 9418<br>Fax: 0131 242 9447<br>Email: fiach.o'mahony@ed.ac.uk
**Co-sponsor Representative**<br><br>Heather Charles<br>Office E1.13<br>Research & Development Management Suite<br>The Queen's Medical Research Institute<br>47 Little France Crescent<br>Edinburgh EH16 4TJ<br><br>Tel: 0131 242 3359	**Principal Investigator**<br><br>Dr Fergus Doubal<br>Neuroimaging Sciences<br>University of Edinburgh<br>Centre for Clinical Brain Sciences (CCBS)<br>Chancellor's Building<br>49 Little France Crescent<br>Edinburgh EH16 4SB<br><br>Tel: +44 (0)131 465 9599

Fax: 0131 242 3343<br>Email:<br>heather.charles@nhslothian.scot.nhs.uk	Fax: Not available<br>Email: Fergus.Doubal@ed.ac.uk
**Trial Manager**<br><br>Anna Heye<br>Edinburgh Clinical Trials Unit<br>University of Edinburgh<br>Western General Hospital<br>Crewe Road<br>Edinburgh EH4 2XU<br><br>Tel: 0131 651 9917<br>Fax: Not available<br>Email: anna.heye@ed.ac.uk	**Trial Statistician**<br><br>Lisa Woodhouse (a member of Prof A. Montgomery's team)<br>Room B48 Clinical Sciences Building<br>Nottingham City Hospital<br>Hucknall Road<br>Nottingham<br>NG5 1PB<br><br>Tel: 0115 82 31670<br>Fax: Not available<br><br>Email: l.woodhouse@nottingham.ac.uk

PARTICIPATING SITES

The up to date list of participating sites is maintained on the LACI-2 trial website

CONTENTS

PROTOCOL APPROVAL 6

1 INTRODUCTION 10

1.1 BACKGROUND 10

1.2 RATIONALE FOR STUDY 12

2 STUDY OBJECTIVES 14

2.1 OBJECTIVES 14

2.1.1 Primary Objective 14

2.1.2 Secondary Objectives 14

2.2 ENDPOINTS 14

2.2.1 Primary Endpoint 14

2.2.2 Secondary Endpoints 14

3 STUDY DESIGN 15

4 STUDY POPULATION 17

4.1 NUMBER OF PARTICIPANTS 17

4.2 INCLUSION CRITERIA 17

4.3 EXCLUSION CRITERIA 17

4.3.1 General exclusion criteria 18

4.3.2 Cilostazol exclusion criteria (still allows randomisation to ISMN) 18

4.3.3 ISMN exclusion criteria (still allows randomisation to Cilostazol) 18

4.4 CO-ENROLMENT 19

5 PARTICIPANT SELECTION AND ENROLMENT 19

5.1 IDENTIFYING PARTICIPANTS 19

5.2 CONSENTING PARTICIPANTS 19

5.3 SCREENING FOR ELIGIBILITY 20

5.4 INELIGIBLE AND NON-RECRUITED PARTICIPANTS 21

5.5 RANDOMISATION 21

5.5.1 Randomisation Procedures 21

5.5.2 Emergency Unblinding Procedures 23

5.5.3 Withdrawal of Study Participants 23

6 INVESTIGATIONAL MEDICINAL PRODUCTS 23

6.1 STUDY DRUGS 23

6.1.1 Study Drug Identification 23

6.1.2 Study Drug Manufacturer 24

6.1.3 Marketing Authorisation Holder 24

6.1.4 Labelling and Packaging 24

6.1.5 Storage 25

6.2 DOSING REGIME 25

6.3 DOSE CHANGES 27

6.4 PARTICIPANT COMPLIANCE 27

6.5 OVERDOSE 27

6.6 OTHER MEDICATIONS 27

6.6.1 Non-Investigational Medicinal Products 27

6.6.2 Permitted Medications 27

6.6.3 Prohibited Medications 27

7 STUDY ASSESSMENTS 28
7.1 SAFETY ASSESSMENTS 28
7.2 STUDY ASSESSMENTS 28
8 DATA COLLECTION 30
9 STATISTICS AND DATA ANALYSIS 31
9.1 SAMPLE SIZE CALCULATION 31
9.2 PROPOSED ANALYSES 32
10 ADVERSE EVENTS 32
10.1 DEFINITIONS 32
10.2 IDENTIFYING AEs AND SAEs 33
10.3 RECORDING AEs AND SAEs 33
10.3.1 Pre-existing Medical Conditions 33
10.3.2 Outcome Events 33
10.3.3 Events That Are Common in the Trial Population 34
10.3.4 All Other Events Not Covered Above 35
10.4 ASSESSMENT OF AEs AND SAEs 38
10.4.1 Assessment of Seriousness 38
10.4.2 Assessment of Causality 38
10.4.3 Assessment of Expectedness 38
10.4.4 Assessment of Severity 38
10.5 REPORTING OF AEs TO THE SPONSOR 39
10.6 REPORTING OF SAEs/SARs/SUSARs 39
10.7 REGULATORY REPORTING REQUIREMENTS 39
10.8 CENTRAL BLINDED REVIEW OF SAEs 39
10.9 FOLLOW UP PROCEDURES 40
11 PREGNANCY 40
12 TRIAL MANAGEMENT AND OVERSIGHT ARRANGEMENTS 40
12.1 TRIAL MANAGEMENT GROUP 40
12.2 TRIAL STEERING COMMITTEE 40
12.3 DATA MONITORING COMMITTEE 41
12.4 INSPECTION OF RECORDS 41
12.5 RISK ASSESSMENT 41
12.6 STUDY MONITORING AND AUDIT 41
13 GOOD CLINICAL PRACTICE 41
13.1 ETHICAL CONDUCT 41
13.2 REGULATORY COMPLIANCE 41
13.3 INVESTIGATOR RESPONSIBILITIES 42
13.3.1 Informed Consent 42
13.3.2 Study Site Staff 42
13.3.3 Data Recording 42
13.3.4 Investigator Documentation 42
13.3.5 GCP Training 42
13.3.6 Confidentiality 43
13.3.7 Data Protection 43
14 STUDY CONDUCT RESPONSIBILITIES 43

14.1 PROTOCOL AMENDMENTS 43
14.2 PROTOCOL VIOLATIONS AND DEVIATIONS 43
14.3 SERIOUS BREACH REQUIREMENTS 44
14.4 STUDY RECORD RETENTION 44
14.5 END OF STUDY 44
14.6 CONTINUATION OF DRUG FOLLOWING THE END OF STUDY 44
14.7 INSURANCE AND INDEMNITY 44
15 REPORTING, PUBLICATIONS AND NOTIFICATION OF RESULTS 45
15.1 AUTHORSHIP POLICY 45
15.2 PUBLICATION 45
15.3 PEER REVIEW 45
16 REFERENCES 46

PROTOCOL APPROVAL

LACunar Intervention (LACI-2) Trial 2: Assessment of safety and efficacy of cilostazol and isosorbide mononitrate to prevent recurrent lacunar stroke and progression of cerebral small vessel disease.

EudraCT number: 2016-002277-35

Professor Joanna Wardlaw<br>Chief Investigator	____________________<br>Signature	____________<br>Date
Lisa Woodhouse<br>Trial Statistician	____________________<br>Signature	____________<br>Date
Dr Fiach O'Mahony<br>Sponsor(s) Representative	____________________<br>Signature	____________<br>Date
Anna Heye<br>Trial Manager	____________________<br>Signature	____________<br>Date

TO BE COMPLETED

LIST OF ABBREVIATIONS

ACCORD	Academic and Clinical Central Office for Research & Development
AE	Adverse Event
AMP	Adenosine Monophosphate
AR	Adverse Reactions
BBB	Blood-Brain Barrier
BP	Blood Pressure
BHF	British Heart Foundation
CA	Competent Authority
CI	Chief Investigator
CO2	Carbon Dioxide
CRF	Case Report Form
CSPS trial	Clinical Stroke Prevention Study
CSPS2 trial	Clinical Stroke Prevention Study 2
CSPS3 trial	Clinical Stroke Prevention Study 3
CT	Computed Tomography
CVR	Cerebrovascular Reactivity
eCRF	Electronic Case Report Form
ENOS	Efficacy of Nitric Oxide in Stroke
GMP	Guanosine Monophosphate
GTN	Glyceryl Trinitrate
ICF	Informed Consent Form
ICH GCP	International Conference on Harmonisation Tripartite Guideline for Good Clinical Practice
ISF	Investigator Site File
ISMN	Isosorbide Mononitrate
IQCODE	Informant questionnaire on cognitive decline in the elderly
LACI-2	LACunar Intervention Trial 2
MMSE	Mini-Mental State Examination
MOCA	Montreal Cognitive Assessment
MRI	Magnetic Resonance Imaging
NO	Nitric Oxide
O2	Oxygen
PDE3	Phosphodiesterase 3
PGI2	Prostaglandin Inhibitor 2
PI	Principal Investigator
PIS	Patient Information Sheet

REC	Research Ethics Committee
RCT	Randomised Controlled Trial
SAE	Serious Adverse Event
SAR	Serious Adverse Reaction
SPC	Summary of Product Characteristics
SPS3	Secondary Prevention of Small Subcortical Strokes Trial
SUSAR	Suspected Unexpected Serious Adverse Reaction
SVD	Small Vessel Disease
TICS	Telephone Interview for Cognitive Status
TMOCA	Telephone Montreal Cognitive Assessment
UK	United Kingdom
USA	United States of America
WMH	White Matter Hyperintensities

SUMMARY

Professional Summary

A quarter of all ischaemic strokes (about 35000 per annum in the UK) are lacunar (small vessel) in type, mainly caused by an intrinsic, non-atheromatous, non-cardioembolic disease of the small deep perforating cerebral arterioles. More diffuse cerebral small vessel disease also causes up to 45% of dementias (350,000+ patients estimated currently in the UK), either alone or in association with Alzheimer’s disease. There is no proven treatment for cerebral small vessel disease: conventional antiplatelet drugs may be ineffective or even hazardous, whilst antihypertensive treatment and statins may not have an effect. The disease mechanism is poorly understood but endothelial dysfunction, blood-brain barrier failure and vessel stiffness appear to contribute to the pathogenesis. Promising data available for licensed drugs with relevant modes of action, cilostazol (>6000 stroke patients in the Asia Pacific Region) and isosorbide mononitrate (ISMN, widely used in cardiac disease) support their testing in cerebral small vessel disease. This trial will be an Phase IIb preparatory to Phase III, randomised, partial factorial, open label, blinded end-point trial, testing cilostazol, ISMN, both, or neither, to assess the feasibility of recruitment, drug tolerability, trial procedures, safety and event rates in 400 patients recruited in UK stroke centres and followed-up to one year (primary endpoint). This trial is preparatory to a large, definitive, Phase III randomised controlled trial to prevent recurrent lacunar stroke and progressive small vessel disease-related physical and cognitive impairments after lacunar stroke.

Lay Summary

About 35,000 people each year in the UK have a type of stroke, called ‘lacunar’ or ‘small vessel’ stroke, which is different to other common types of stroke and for which there is no proven treatment. We think that small vessel stroke is caused by damage to the lining of the tiny blood vessels deep inside the brain that stops them functioning normally. This not only causes stroke but, perhaps more importantly, causes problems with thinking and walking, possibly causing up to 45% of all dementias either on its own, or mixed with Alzheimer’s disease (about 350,000 patients in the UK). Some drugs that are commonly used in other blood vessel diseases may help improve small vessel function and prevent worsening of brain damage. One drug (cilostazol) has been tested in patients with stroke in the Asia Pacific countries but not on dementia; the other drug (isosorbide mononitrate) is widely used in the UK for heart disease but not stroke. We want to set up a clinical trial to test if the study methods are practical so that patients and trial centres can follow the procedures, and to confirm how many patients have more stroke-like symptoms or experience worsening of their thinking skills. This information is needed to be sure that a very large clinical trial to find out if these drugs can prevent worsening of small vessel disease will be possible.

1 INTRODUCTION

1.1 BACKGROUND

Burden of disease: Stroke and dementia rank among the most pressing health issues, show substantial co-morbidity and share many risk factors. Cerebral small vessel disease (SVD) has emerged as a central link between the two disease states.[1, 2] SVD accounts for up to 45% of all dementias, either alone or combined with Alzheimer's disease.[1, 3] SVD also causes 20-25% of clinical strokes (lacunar stroke)[4], about 35,000 per year in the UK, and leaves at least a third of these patients with cognitive impairment or dementia after the stroke.[5, 6] Patients with lacunar stroke are often younger than for other stroke subtypes[7] and cognitive impairment restricts their return to work not just independence. In addition, about 50% of 65 year olds and almost all 90 year olds, have imaging manifestations of SVD,[8] which build up insidiously and diffusely in the brain until of sufficient severity to cause symptoms. Both stroke and vascular dementia are major Government targets, are increasing in prevalence,[9] and have enormous economic and societal costs.

SVD is easily detected on magnetic resonance brain imaging (MRI) as white matter hyperintensities (WMH), lacunes, microbleeds, prominence of perivascular spaces and brain atrophy.[10] Individually, and when mild, these imaging features are often clinically silent. However, when more severe, they cause cognitive impairment, physical disability and depression. They are associated with worse outcomes after acute stroke,[11] increase the risk of developing dementia (2-fold) and of having a stroke (3-fold).[12, 13] A simple visual sum score may capture the overall burden of total SVD brain damage better than the individual separate features, and highlights that the importance of some risk factors for SVD, like smoking, may have been under-recognised.[14, 15]

Pathology of lacunar stroke: Despite this profound impact on human health, there are no treatments with proven efficacy that prevent progression of SVD and its clinical or imaging manifestations.[16] In part this reflects our limited understanding of the cause of SVD, and how it affects the brain. Less than 11% of clinically-evident lacunar ischaemic strokes are atherothromboembolic.[17] Lacunar stroke patients have less large artery atheroma (less ischaemic heart disease, carotid stenosis, peripheral vascular disease (PVD)), despite similar rates of hypertension and diabetes, to those with atheromatous stroke.[7] Risk factors for SVD in general include hypertension, smoking, diabetes,[4] but all common vascular risk factors combined explain less than 2% of variance in SVD features.[18]

Instead, most lacunar stroke is due to an intrinsic deep perforating arteriolar disease with arteriolar wall thickening, mural and perivascular inflammation, segmental arteriolar wall disintegration,[19] and perivascular brain damage.[2] The arteriolar damage is linked with cerebral endothelial dysfunction, impaired vasoreactivity,[20, 21] and increased blood-brain barrier (BBB) permeability,[2, 22, 23] all of which increase secondary ischaemic brain damage. Additionally, the damaged endothelium may precipitate local thrombosis, further worsening ischaemic damage.[2] Subtle BBB failure is a key component of the pathology,[3, 24-26] which could explain the thickening and stiffening of arteriole walls, and subsequent increase in WMH.[21, 27, 28] Systemic arteriolar stiffness may also account, at least partly, for the link between hypertension and WMH.[29] Stiff arterioles do not vasodilate well to increase blood supply in response to increased brain activity, thus potentially increasing secondary ischaemic damage and dementia.[30] Impaired cerebrovascular reactivity (CVR) is also found in Alzheimer's dementia[31] and amyloid angiopathy [32] making endothelial dysfunction an important potential SVD therapeutic target.

Prevention of lacunar stroke is suboptimal: Although many patients with lacunar stroke and other SVD features must have been included in many previous stroke prevention randomised clinical trials (RCTs), few reported their results by stroke subtype. Where subtyping was reported, it was often suboptimal, mixing lacunar and athero-thrombo-embolic or cardio-embolic strokes. There have been few RCTs specifically in lacunar stroke. The largest of these, the Secondary Prevention of Small Subcortical Stroke (SPS3) trial (3000+ lacunar stroke patients), tested aspirin+clopidogrel vs aspirin and target vs guideline BP reduction to prevent recurrent stroke and cognitive decline. The aspirin+clopidogrel vs aspirin arm stopped early as

aspirin+clopidogrel increased bleeding and death[33] without reducing recurrent stroke[33] or MI,[34] more evidence of the non-atheromatous nature of SVD. Target (vs guideline) BP lowering was consistent with a small reduction in recurrent stroke,[35] but neither it nor aspirin+clopidogrel vs aspirin reduced cognitive decline.[36] There are few data on statins[16] but statins did not prevent WMH progression in PROSPER.[37] However, statins are guideline therapy after stroke and their anti-inflammatory effects may benefit SVD.

Implications of current management: Current guideline-based secondary prevention of lacunar stroke with antiplatelet, antihypertensive drugs and statins has a limited evidence base, may be ineffective, or even hazardous. The burden of SVD, in particular its stroke and dementia consequences, is increasing with longevity. Brain haemorrhage also increases with age,[38] SVD features and antiplatelet drugs.[34] Other approaches are therefore needed to reduce the clinical impact of SVD.

Therapeutic targets: In light of the SVD mechanisms outlined above, we (and others[39]) reviewed all potential drugs tested in RCTs that included lacunar stroke and found many relevant drugs.[16] In a systematic review of experimental models of SVD,[40, 41] we also found promising data on several drugs available for human use. We particularly looked for available licensed drugs with relevant effects: to stimulate the nitric oxide (NO)-cyclic GMP or Prostacyclin (PGI2)-cyclic AMP systems thereby improving vasodilatation, reducing inflammation and smooth muscle hypertrophy (to reduce stiffness) and improving cerebral endothelial integrity (prevent extra-vascular leakage) and neuroprotective effects.[2] Two drugs, both licensed in Europe for treatment of vascular diseases, show promise.

Cilostazol is a phosphodiesterase 3-inhibitor (PDE3-inhibitor) that enhances the PGI2-cAMP system. It has weak antiplatelet effects (so low bleeding risk),[42] reduces infarct size[41] and reduces ageing-related decline in myelin repair[43] in experimental models, has a UK license for treatment of peripheral vascular disease, and has data from trials including more than 6400 patients with stroke (Figure 1).[44-47] Amongst those, the trials where >50% of participants had lacunar stroke (n=4780) found long-term cilostazol (vs placebo or aspirin) reduced recurrent stroke (OR 0.62, 95%CI 0.49-0.77) without increasing haemorrhage (OR 0.52, 95%CI 0.36-0.75), or death (OR 0.90 95%CI 0.53-1.52) over median 1 year treatment (Figure 1). However, there are no data on the effects of cilostazol on cognition, or on imaging features of SVD, and all data are from Asia-Pacific region countries where the epidemiology, range of risk factors, stroke subtype profiles, and other vascular characteristics differ from those commonly seen in the West.

Nakamura 47%	2	38	3	38	1.3%	0.67 [0.12, 3.77]
CAIST 55%	5	231	9	227	3.4%	0.55 [0.19, 1.60]
CSPS 2 65%	82	1337	119	1335	54.0%	0.69 [0.52, 0.90]
TOHOKU (Shimizu) 67%	5	251	6	256	2.9%	0.85 [0.26, 2.75]
CSPS 75%	34	533	64	534	25.0%	0.53 [0.36, 0.79]
Subtotal (95% CI)		2390		2390	86.6%	0.64 [0.51, 0.79]
Total events	128		201			

Heterogeneity: Tau² = 0.00; Chi² = 1.41, df = 4 (P = 0.84); I² = 0%
Test for overall effect: Z = 4.13 (P < 0.0001)

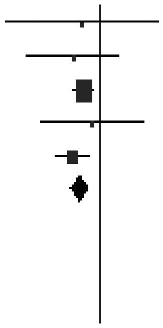

Figure 1. Meta-analysis of trials of cilostazol for stroke prevention. % indicates proportion of patients recruited with lacunar stroke.

Isosorbide mononitrate (ISMN), is an NO donating organic nitrate that enhances vasodilation, is widely used in ischaemic heart disease, and has no antiplatelet activity.[48] NO is an important regulator of cerebral haemodynamic function which is impaired in patients with lacunar stroke and WMH.[21] Replacing NO might improve vasoreactivity,[49] but drugs that increase NO availability are rarely used in stroke, particularly lacunar stroke (e.g. pre-stroke nitrates were used in <2% in patients randomised in the Efficacy of Nitric Oxide in Stroke (ENOS) trial including those randomised into the trial with lacunar syndromes[50] or in our lacunar stroke cohort studies[14]). This is surprising since ISMN is well established in the UK for cardiac disease with a known safety profile, endogenous NO is reduced in patients with SVD,[21] so replacing NO

might improve vessel function.[49] The related drug, transdermal GTN, another organic nitrate, administered short term after acute stroke, improved cerebral perfusion,[51] vascular reactivity and functional outcome, including cognition, at three months after stroke in ENOS, when started within three hours of acute stroke and given for up to seven days after stroke.[50] These data were sufficiently promising for the BHF to fund a new trial of GTN in hyperacute stroke, RIGHT-2 (ongoing).

Use of two drugs with complementary actions that affect different parts of the pathological pathway could increase benefit over one drug alone. The importance of vascular disease in neurodegeneration is now being recognised,[10, 52] together with the importance of SVD as a bridge between stroke and dementia.[1] The contributing mechanisms to target are clearer[2] and licensed drugs are available.[16, 39]

1.2 RATIONALE FOR STUDY

The need for a trial in lacunar stroke: There is no established treatment to prevent recurrent lacunar stroke or other features of SVD. The disparity between the apparent benefits of cilostazol in stroke prevention in the Asia-Pacific region and its lack of use in the UK is striking and supports evaluation. Cilostazol is available worldwide and generics are now available in the UK. Similarly, ISMN is widely used in cardiology, is available as a generic, but its potential benefits in stroke have received little attention despite relevant NO actions, an oversight that also needs to be addressed. As well as effective treatments, we need more data for designing trials in lacunar stroke: on recruitment and assessment methods; long term event rates; on safety and tolerance of promising drugs to undertake large Phase III trials.

Mechanisms by which Cilostazol and/or ISMN might work: Cerebral haemodynamic function is impaired in lacunar stroke and SVD.[21] Cilostazol is a PDE3-inhibitor that preserves cAMP and should reduce cerebral microvessel vasoconstriction, smooth muscle proliferation and inflammation, secondary brain damage, protect the endothelium,[53] and may enhance myelin repair.[43] Importantly, in SVD where there may be increased bleeding risk, cilostazol has low bleeding risk.[42] Cilostazol was trialled in Japan, China and Korea, with more trials ongoing in Japan (CSPS3, Toyoda, collaborator), China and Taiwan.[54] Promising data on reduction in recurrent stroke (Figure 1) and improving cerebral arterial stiffness in human[55] and experimental[53, 56] SVD, support testing in Europeans. Effects of Cilostazol on cognitive and physical function in SVD are unknown.

ISMN replaces NO, which is low in acute lacunar stroke,[57] so should also enhance cerebral endothelial function and vasodilatation.[49] ISMN has no antiplatelet effects. Endothelial dysfunction is well documented in SVD.[1, 2, 39] There is little experience with nitrates in patients with lacunar stroke,[14, 50] because conventional large artery atheromatous indications such as angina are infrequent.[7, 34]

Combined stimulation of the NO-cGMP and PGI2-cAMP systems potentially maximises the above benefits, if it does not increase adverse effects, so cilostazol and ISMN together may be more effective than either drug alone. Both may improve BP control, lower BP modestly, reduce BP variability, further reducing stroke and SVD risk. Combining ISMN (no antiplatelet effects) and cilostazol (weak antiplatelet) should avoid excess bleeding when given with guideline clopidogrel or aspirin (both moderate-strong antiplatelet agents).[33]

Rationale for the approach: The trial was designed as part of an NIHR SRN-Portfolio Development Expert Writing Group using experience from the USA NIH-funded SPS3 trial (3000+ lacunar stroke patients, CI Benavente; SRN Writing Group, LACI-2 International Advisor to TSC),[33, 35, 36] the CSPS and CSPS2 trials (3740 patients[44, 46]) and ongoing CSPS3 trial testing cilostazol to prevent stroke in Japan (PI Toyoda, International Advisor to TSC), the PRESERVE trial (CI Markus, SRN Writing Group), and in monogenic SVD (Chabriat, SRN Writing Group, International Advisor to TSC). UKSRN Prevention CSG strongly supported a trial testing cilostazol and ISMN in SVD prevention: many (>80) UK Stroke Research Network Centres expressed interest in joining the trial.

Relevant drugs are available: Although many experts are uncertain of what agents to test in lacunar stroke,[33, 35] we identified targets with available drugs.[10, 16, 41] Other trials are testing BP-lowering on cognition in patients with combined lacunar stroke and severe WMH (BHF PRESERVE, CI Markus, UK). However, other approaches are clearly needed since hypertension explains <1.5% of variance in WMH,[18] target BP reduction did not prevent stroke or cognitive decline in SPS3,[35] may increase stroke[58] and SVD worsening at older ages.[59] There is increasing uncertainty about antiplatelet drugs: in SPS3, they increased hazard without benefit.[33, 37] SVD is now recognised as a major cause of haemorrhagic stroke[60] especially at older ages.

Experience with cilostazol and ISMN are lacking in Europe: Despite the large amount of promising data from the Asia-Pacific region for cilostazol (Figure 1) and in European cardiology for ISMN, and both drugs being licensed in Europe for vascular indications with available generic forms, there is little experience with cilostazol for vascular disease in Europe or North America and little experience of long term administration of ISMN or related nitrate donors in any type of stroke. Therefore, information on patient tolerability (e.g. cilostazol and ISMN cause headache), safety (e.g. platelet function, postural hypotension, bleeding), markers of efficacy (reduction in stroke recurrence), or on whether both drugs can be taken together in full dose, is lacking. Information on safety is important given that a) cilostazol will need to be given in addition to guideline secondary antiplatelet stroke prevention which might increase bleeding (despite little evidence of antiplatelet effects of cilostazol combined with other antiplatelet drugs[42,33]), and b) ISMN may reduce BP leading to postural hypotension and falls or accelerate brain white matter damage in older patients, which may concern physicians. Thus information on these points is necessary in order to justify a large Phase III trial of cilostazol and/or ISMN to prevent stroke and dementia manifestations of SVD and justify further testing of cilostazol and/or ISMN.

LACI-1 Pilot trial: The LACI-2 trial design is based on experience gained by the LACI-2 investigators with cilostazol and ISMN in patients with lacunar ischaemic stroke in a pilot trial funded by the Alzheimer's Society. LACI-1 started recruitment March 2016, is recruiting 60 patients in two UK centres (Nottingham, Edinburgh), and testing short-term dose escalation protocols for cilostazol and ISMN, individually and together, to the dose planned for LACI-2, and records tolerability, safety and early markers of efficacy.

Many patients may benefit: If cilostazol and/or ISMN can be administered safely to patients with clinical evidence of SVD, and if large scale trials demonstrate that they are effective in preventing clinical progression of SVD, then large proportions of patients with stroke and dementia will benefit as SVD is such a common cause of these conditions. Up to 35,000 new patients per annum with stroke and several million patients with cognitive decline or incipient dementia in the UK will benefit each year. Both drugs are licensed in Europe and available in generic form, therefore both interventions will be inexpensive to the NHS. Both drugs are taken orally, once or twice daily, so are easy to administer. The long-term impact of successful treatment is difficult to quantify but potentially, a reduction of 10-20% in the combined stroke, cognitive or physical consequences of SVD would benefit several tens of thousands of patients per year in the UK.

The present trial aims to determine if either or both cilostazol and ISMN are tolerated at the target dose for at least a year, gather data on outcome event rates and provide safety and efficacy data. Both drugs cause headache, a marker of vasoreactivity[61] that could limit tolerance, but as with other side effects (palpitations, nausea), our international advisor indicates that these can minimised by starting at half dose, at night, escalating slowly to full dose over several weeks. LACI-2 will proceed seamlessly to a larger phase III trial, with additional funding, once there is sufficient confidence in trial procedures, event rates and recruitment. LACI-2 will also provide methods for balancing randomisation, streamlining of follow-up and reliable data on key event rates for lacunar stroke/SVD trials, promoting more personalised approaches to brain vascular disease required by the key differences between stroke subtypes and their mechanisms.

2 STUDY OBJECTIVES

2.1 OBJECTIVES

Our ultimate goal is to prevent progression of cerebral small vessel disease (SVD) and its clinical consequences of stroke, dementia and physical disability. We have identified that two drugs, licensed for other vascular disease indications, cilostazol and isosorbide mononitrate (ISMN), have relevant mechanisms and some promising data that supports testing in an early Phase III trial, prior to a large definitive Phase III trial.

The **LACunar Intervention** Trial-**2** (LACI-2) will assess feasibility of recruitment, drug tolerability, trial procedures, safety and event rates in at least 400 patients recruited in UK stroke centres and followed up for one year. Safety will be assessed in an MR follow-up study as well as with clinical outcomes in all patients. Funding to continue LACI-2 into a phase III trial will be sought once the initial phases of LACI-2 have provided confidence about estimated event rates and hence sample size, recruitment and trial procedures.

Cilostazol and ISMN will need to be administered in addition to guideline therapies (usually clopidogrel; antihypertensives; statins). Data on effects on safety outcomes, WMH progression and new infarcts on brain MRI, and on recurrent clinical vascular events, cognition, and on trial procedures, will help provide confidence that the drugs are safe and testable in this population in a large-scale RCT with clinical only endpoints and that such a trial is likely to achieve the sample size likely to be required to demonstrate definitively clinically-meaningful reductions in SVD progression.

2.1.1 Primary Objective

To determine whether a prospective, randomised trial of cilostazol and ISMN in lacunar stroke is feasible in the UK, thence proceeding seamlessly to a large phase III trial.

2.1.2 Secondary Objectives

To assess drug tolerability, safety, event and recruitment rates preparatory to a large Phase III partial factorial randomised controlled trial to prevent recurrent lacunar stroke, physical and cognitive impairment.

2.2 ENDPOINTS

2.2.1 Primary Endpoint

Feasibility of Phase III trial, i.e. that eligible patients can be identified correctly, in sufficient numbers, enrolled and >95% retained in follow-up at one year, to achieve feasibility target sample size recruitment and randomisation of 400 patients in 24 months in the UK.

2.2.2 Secondary Endpoints

<u>Tolerability</u> - 75% of patients will be able to tolerate trial medication, in at least half dose, up to one year after randomisation (i.e. less than 25% will stop trial medication completely through inability to tolerate the drugs).

<u>Safety</u> - symptoms of systemic or intracranial bleeding, recurrent cerebral and systemic vascular events, and vascular and non-vascular causes of death will be collected. The absolute risk of death, including fatal haemorrhage, will not differ significantly (ie fall outside the upper 95% CI) from 2% per year on trial drugs versus no trial drugs, when given in addition to guideline drugs; and will not increase bleeding or ischaemic SVD lesions significantly (at the p<0.01 level) on MRI.

<u>Efficacy</u> – individual event-rate data (stroke, TIA, myocardial ischaemia, cognitive impairment and dementia, will be collected. The *combined rate* of recurrent stroke, MI, death, cognitive impairment and dependency will be 40-50% at one year after enrolment in order to detect modest but clinically-important reductions in poor outcomes.

Health Economics – data to assess health economic impact will be gathered.

3 STUDY DESIGN

LACI-2 will be an investigator-led, Phase IIb preparatory to Phase III, prospective, randomised, 2x2 partial factorial, open label, blinded outcome trial performed in multiple hospital-based centres. The schematic in Figure 2 describes the study design. This Phase will test trial procedures and feasibility, tolerability of drug, provide safety and intermediary outcome endpoints and clinical and cognitive endpoints to power a main Phase III trial which will follow seamlessly from LACI-2 with additional funding. Brain imaging with MRI is to be used in preference to CT for diagnosis of the index stroke wherever possible, but CT is allowed.

Patients who have all of the following:
1) suffered a minor stroke with clinical features compatible with a lacunar stroke syndrome (designated as the 'index stroke');
2) on contemporaneous brain imaging have either

> a) on MR imaging, a recent, relevant (in time and location) acute lacunar infarct on diffusion MR imaging, or
> b) if no visible acute lacunar infarct on diffusion MR imaging then there may be a recent-appearing relevant (in time and location) acute lacunar infarct on FLAIR, T2, or T1, and
> c) there will be no competing pathology as a cause for stroke (e.g. no acute cortical infarct on diffusion MR imaging or recent-appearing cortical infarct on FLAIR, T2, T1), no intra-cerebral haemorrhage, no stroke mimic such as tumour, or subdural haematoma);
> d) where CT brain imaging is used soon after stroke instead of MR, a recent relevant (in time and location) small subcortical infarct (e.g. that was not present on CT prior to the stroke, or that becomes visible on a follow-up scan, if available) and no evidence of alternative causes of the stroke symptoms as in 2c above.

3) capacity to give consent; and
4) meet inclusion/exclusion criteria below (4.2),
will be randomised to start one of four treatments; isosorbide mononitrate only; cilostazol only; both isosorbide mononitrate and cilostazol; or neither isosorbide mononitrate nor cilostazol.

Patients with contraindications to cilostazol can be randomised to isosorbide mononitrate versus no isosorbide mononitrate arms only; patients with contraindications to isosorbide mononitrate can be randomised to cilostazol versus no cilostazol arms only. The partial factorial design allows testing of both drugs when given alone and together.

The doses will be escalated until participants are on their full dose by 1 month. If a patient encounters intolerable side effects at full dose, then they will be able to remain on the highest dose regime that they can tolerate and this dose will be recorded. The total duration of the trial drug administration will be 1 year.

Note that no maximum time since the index lacunar ischaemic stroke will be set – patients will be eligible for the trial for several years after their lacunar stroke at the discretion of the local medical staff, as long as the index stroke was compatible with lacunar ischaemic stroke and competing pathologies as a cause of the stroke were excluded using contemporaneous MR imaging, or CT, as above. That is, that the index stroke was not a non-lacunar stroke, that it was ischaemic and not haemorrhagic, and that it was not a non-stroke based on clinical and MR imaging, or CT, acquired at the time of the stroke.

Note also that no minimum cut off cognitive score is set. The Montreal Cognitive Assessment (MOCA) will be collected at baseline but will not be an inclusion/exclusion criterion. The important point is that the patient has capacity to consent, in the opinion of the randomising doctor.

Throughout the trial, patients will continue to take their normal prescribed medication which will include stroke secondary prevention prescribed since their event as per national guidelines/usual post-stroke care in participating centres.

Four hundred patients will be recruited from UK stroke research network centres. The most active of these sites see 10-15 patients per month with suspected lacunar stroke, of whom 25-33% would be eligible. Some interested sites have indicated that they could randomise 1-2 per month. Patients will be recruited from hospital-based stroke services, including patients who have participated in previous completed studies and indicated that they would like to be informed of future stroke studies.

On entry to the study, participants will be assessed by the study researcher, including presenting history, past medical history, medication history, neurological examination, check of brain MR or CT scan, blood pressure measurement and cognitive testing. They will then be randomised.

Patients will receive a telephone call at 1-2 weeks post randomisation into the study and again after 3-4 weeks, with intermediary phone calls as necessary, to ascertain that they have escalated the dose and have achieved the full dose or are established on a tolerated dose if not the full dose. Further follow up by site or central Trial staff will occur at six months (phone/post) and 12 months (obtain MRI, phone/post/in person).

Figure 2. Study flow diagram

*Diagnostic MR or CT brain scan assessment (Baseline) refers to a visual assessment of the scan that has already been performed as part of the patient's routine stroke clinical assessment and diagnosis. Features present on the scan are scored for their presence and severity to create a total sum of small vessel disease score. This is used in the minimisation algorithm.
Dispensing may be at 3-monthly intervals if preferred in particular centres.

The patient will return to hospital to collect their next prescription every three or six months and for an MRI at 12 months.

After the end of 52 weeks, the patients will reduce the tablets over about two weeks and then stop the medication (section 6.2). They will return any unused drug to their local hospital pharmacy for tablet counting and then destruction. They will complete participation in the study after a final phone call when they are off the medication.

Participants who stop IMP (e.g. through tolerability issues or for medical reasons) will remain in the trial in follow-up unless they withdraw consent for further participation in the trial. Patients may withdraw consent at any time, in the event of which the trial will not collect further data on that patient. Data already collected will be kept and included in the analysis unless participants specifically ask for their data not to be included. Study medication maybe withdrawn in line with SPC guidelines at any time by the investigator or treating physician if deemed advisable for medical reasons, but the patient will remain in follow-up unless they withdraw consent.

4 STUDY POPULATION

4.1 NUMBER OF PARTICIPANTS

About 400 participants will be recruited from multiple UK sites.

4.2 INCLUSION CRITERIA

- Clinical lacunar stroke syndrome.
- Brain scanning* with MR including diffusion imaging wherever possible, and obtained soon after the presentation with stroke, shows either:
    - a recent, relevant (in time and location) acute lacunar infarct on diffusion MR imaging[1],
    - or, if no visible acute lacunar infarct on diffusion MR imaging[2] then there is no competing pathology as a cause for stroke (e.g. no acute cortical infarct, no acute intra-cerebral haemorrhage, no stroke mimic such as tumour, subdural haematoma);
    - or, if only a CT brain scan is available[2] as in section 3 above, then there is a small relevant (in age and location) subcortical infarct, or if no infarct then there is no competing pathology as a cause for stroke (e.g. no acute cortical infarct, no acute intra-cerebral haemorrhage, no stroke mimic such as tumour, subdural haematoma).

  [1]*Note that if there is no acute lacunar infarct on MR diffusion imaging but there is a recent-appearing lacunar infarct on FLAIR, T2, or T1 (i.e. no cavitation or ex-vacuo effect; may be slightly swollen, ill-defined edges; or scan in the few weeks before the stroke does not show a lesion but there is an acute lacunar infarct on MR T2, FLAIR, T1 scanning after the stroke in an appropriate area of the brain for symptoms), then the T2, FLAIR, T1 lesion may be counted as the acute lacunar infarct in the absence of a diffusion lesion. Similarly, on CT*[2] *a recent relevant small subcortical infarct would not show cavitation or shrinkage/ex vacuo effect.*

  [2]*Note that about a third of patients with a clinically definite lacunar syndrome do not have a corresponding recent infarct visible on MRI but should still be classed as 'lacunar stroke' if no other explanation can be found for the symptoms. The presence of a recent cortical infarct on FLAIR, T2, T1, the recent timing being indicated by the characteristics above, would count as a competing pathology.*

  Note that the complete absence of any abnormality on MR or CT brain imaging (no acute subcortical infarct or pre-existing SVD such as white matter hyperintensities, lacunes, etc.) while occasionally seen in lacunar stroke is unusual and should question the diagnosis of lacunar ischaemic stroke.
- Age > 30 years
- Independent in activities of daily living (modified Rankin ≤2)
- Capacity to give consent themselves

4.3 EXCLUSION CRITERIA

4.3.1 General exclusion criteria

- Other significant active neurological illness present since suffering stroke (e.g. recurrent seizures, multiple sclerosis, brain tumour). Well-controlled epilepsy present prior to the stroke, a single seizure at onset of the stroke or provoked seizure is not an exclusion.
- Requiring assistance with activities of daily living (Modified Rankin ≥3)
- Has been diagnosed as having dementia on formal clinical assessment
- Diagnosis of hypotension, defined as sitting systolic blood pressure less than 100mmHg
- Unable to swallow tablets
- Planned surgery during the trial period including carotid endarterectomy. Note prior and apparently successful carotid endarterectomy (or other surgery) is not an exclusion criterion and patients who would otherwise be eligible but require endarterectomy first may be randomised after recovery from successful endarterectomy.
- Other concurrent life threatening illness
- Unlikely to be available for follow-up (e.g. moving outside or visitor to the area)
- History of drug overdose or attempted suicide or significant active mental illness
- Pregnant or breastfeeding women, women of childbearing age not taking contraception. Acceptable contraception in women of childbearing age is a “highly effective” contraceptive measure as defined by the Clinical Trials Facilitation Group (http://www.hma.eu/fileadmin/dateien/Human_Medicines/01-About_HMA/Working_Groups/CTFG/2014_09_HMA_CTFG_Contraception.pdf) and includes combined (oestrogen and progesterone containing) or progesterone-only contraception associated with inhibition of ovulation, or intrauterine device or bilateral tubal occlusion. Contraception must be continued for up to 30 days after the end of the IMP dosing schedule.
- Renal impairment (creatinine clearance <25 ml/min)
- Hepatic impairment
- Current enrolment in another Clinical Trial of Investigational Medicinal Product (CTIMP); still in extended follow-up beyond the CTIMP primary outcome and no longer taking that trial’s IMP is not an exclusion to enrolment in LACI-2.
- Unable to tolerate MRI or contraindication to MRI (Claustrophobia, Pacemaker)

4.3.2 Cilostazol exclusion criteria (still allows randomisation to ISMN)

- Definite indication for (i.e. already prescribed) Cilostazol, or definite contraindication to Cilostazol as per SPCs.
- Prohibited medications to Cilostazol (see sections 4.5 of the appended SPCs and protocol section 6.6.3, plus no anticoagulant drugs).
- Active cardiac disease (atrial fibrillation, myocardial infarction in past 6 months, active angina, symptomatic cardiac failure).
- Bleeding tendency (e.g. known platelets<100, active peptic ulcer, history of intracranial haemorrhage such as subdural haematoma, subarachnoid haemorrhage, intracerebral haemorrhage, but not asymptomatic haemorrhagic transformation of infarction or a few microbleeds, taking anticoagulant medication).

4.3.3 ISMN exclusion criteria (still allows randomisation to Cilostazol)

- Definite indication for (i.e. already prescribed) ISMN, or definite contraindication to ISMN as per SPCs.
- Prohibited medications to ISMN (see sections 4.5 of the appended SPCs and protocol section 6.6.3).

4.4 CO-ENROLMENT

If **a participant has already been enrolled into another CTIMP, they cannot be enrolled into this trial. If a patient is enrolled into LACI-2, they may not subsequently be enrolled into another CTIMP whilst they are randomised and participating in LACI-2.**
Inclusion in another non-CTIMP research study is permissible as long as the Chief Investigators of both studies and the Sponsor have agreed to co-enrolment (see ACCORD guideline: http://www.accord.scot/sites/default/files/GL001%20Guidelines%20for%20Co-Enrolment%20v2.0.pdf).

5 PARTICIPANT SELECTION AND ENROLMENT

5.1 IDENTIFYING PARTICIPANTS

Participants will be recruited from stroke services in participating NIHR Clinical Research Network sites. This will include patients presenting for the first time with a new stroke or recurrent stroke and patients who presented to the stroke services of participating sites in the past. Furthermore, participants will be recruited from existing registries, studies and trials where participants remain in long term contact, meet eligibility criteria, and have indicated that they are willing to be contacted about new studies.

Patients under the care of the stroke services will initially be approached by a member of the participant's usual consultant-lead clinical care team (which includes the researcher teams at some locations). This initial contact will take place in person, by phone or by post following local clinical practice. The member of the clinical care team will inform the patient about the trial and provide them with the Information Sheet. If, after reviewing the Information sheet, the patient expresses an interest and requests further information, s/he will then be contacted directly by a member of the research team. Patients from existing registries (including National Databases such as SHARE), studies and trials will only be contacted by researchers if they previously indicated that they were happy to receive information about new studies, via approved SHARE or relevant procedures.

The investigator or their nominee will inform the patient of all aspects pertaining to participation in the trial. It will be explained to the potential participant that entry into the trial is entirely voluntary and that their treatment and care will not be affected by their decision. It will also be explained that they can withdraw at any time. In the event of their withdrawal it will be determined if the patient agrees that the data collected so far can be retained in the trial analyses where appropriate so as not to jeopardise the scientific credibility of the trial. The discussion of the trial and preferred method of contact will be recorded in the patients' notes. Patients will be given as much time as they require (at least 24 hours) to consider the trial information before deciding whether to join the trial or not. After allowing time for consideration, patients will be contacted by a member of the research team (clinical research fellow or research nurse) using their preferred contact method to find out if they are interested or not. Patients who are interested will be invited to attend for the first assessment visit and an appointment arranged.

5.2 CONSENTING PARTICIPANTS

Patients will give informed written consent upon entry to the trial (baseline), prior to any trial related procedures taking place. The decision to participate in clinical research is voluntary and should only be based on a clear understanding of what is involved.

Consent will only be taken from the patient. Patients unable to give consent for any reason will be excluded, including those considered to lack capacity by medical staff. Patients who are found to have an exclusion criterion after consent but before randomisation, will be recorded as 'consented but not randomised', will therefore not constitute part of the intention to treat population, will be excluded from the primary analysis and will not be followed for AEs.

Consent will be obtained by Good Clinical Practice trained staff who are members of the clinical research team after full discussion of the trial procedures and requirements with the patient. The Investigator or designated person is responsible for ensuring that the consent form is completed, signed and dated by all parties prior to any protocol specific procedures being carried out. Participants must receive adequate oral and written information – appropriate Participant Information Sheet (PIS) and Informed Consent Form (ICF) will be provided. The oral explanation to the participant should be performed by the Investigator or designated person, and must cover all elements specified in the PIS/ICF. The participant must be given every opportunity to clarify any points that they do not understand and, if necessary, ask for more information. It should be emphasised that the participant may withdraw their consent to participate at any time without loss of clinical care benefits to which he/she would be entitled.

The participant will be asked for their permission to contact a relative/partner/carer in case of difficulty in contacting themselves during follow-up and who could and would be willing to provide information about the participant during follow-up in the event that the participant themselves becomes incapacitated and unable to provide the information during the course of the trial. If there is no suitable relative/partner/carer, or the participant is not willing to have such a person approached, then they can still participate in the trial and their wishes will be noted in the site file, hospital notes and eCRF. An information sheet and consent form about the trial for relatives/partners/carers will be given to the participant, or posted or given directly to the relative/partner/carer if accompanying the participant, at the start of the trial. At this point relatives/partners/carers contact details will be stored in the site files only. Consent from the relative/partner/carer for central storage of their contact details and their involvement in the trial will be sought. If consent is given the relative/partner/carer's contact details will be stored in a secure, encrypted, password-protected database linked to the participant number, enabling the follow-up to be done centrally. The absence of a nominated informant will not be counted as a protocol deviation or violation. A 12 month follow-up pack will be posted to the relative/partner/carer. This will contain a covering letter, the Informant Questionnaire on Cognitive Decline in the Elderly, IQCODE and a prepaid envelope for return. The relative/partner/carer will be asked to complete and return the questionnaire. It is their decision as to whether they complete the form or not. Data may be collected by phone from the relative if the questionnaire is not returned, unless the relative/partner/carer has not agreed to this.

The participant should be informed and agree to their medical records being inspected by regulatory authorities and representatives of the sponsor. They should also be informed about and agree to the possibility of further analyses of the trial data (anonymised) as new relevant questions arise, the contribution of the data to individual patient data meta-analyses and the need to make the trial dataset publicly available as per research funder requirements.

The date that the patient is given the PIS should be documented in the patient's medical records. The Investigator or delegated member of the trial team and the participant should sign and date the ICF(s) to confirm that consent has been obtained. The participant should receive a copy of this document, a copy should be filed in the patient's medical records (a PDF will be uploaded to the medical record on sites where medical records are only held electronically) and the original filed in the Investigator Site File (ISF). Full details of the consent process should also be recorded in the patient's medical records. A copy of the PIS should be filed in the patient's medical notes. The patient should retain their copy of the PIS, and a copy of the completed consent form.

5.3 SCREENING FOR ELIGIBILITY

Following consent, the research doctor will confirm that the patient is eligible to participate in the trial as per the inclusion/exclusion criteria. Eligibility will be documented by the PI (or a medically qualified doctor who has been delegated this role) in the participant's medical notes. The baseline information required for randomisation will be recorded in the eCRF.

5.4 INELIGIBLE AND NON-RECRUITED PARTICIPANTS

Participants who have been approached for the study and subsequently found to be ineligible will be informed of the reasons why and continue under the care of their stroke physician. Screening logs will not be used as part of the data collection for this study.

5.5 RANDOMISATION

5.5.1 Randomisation Procedures

The electronic randomisation system and trial database are hosted by the University of Nottingham. Randomisation involves minimisation on key prognostic factors including: age, sex, stroke severity (NIHSS), dependency resulting from the stroke, systolic blood pressure ≤/>140, smoker status, time after stroke, years of education. Years of education give an estimate of pre-morbid cognitive ability and predicts post-stroke cognitive impairment; BP and smoking predict recurrent stroke; delay since stroke reflects disease activity; age, sex and stroke severity are standard minimisation variables. This approach ensures concealment of allocation (note allocation concealment means that it is not possible to guess which treatment the next patient will be randomised to), minimises differences in key baseline prognostic variables, and improves statistical power. Randomisation will not be minimised by Centre because this may result in high rates of allocation prediction, but a pre-specified post-hoc analysis by centre will be performed to investigate and adjust for heterogeneity of treatment effect by centre.

Randomisation will allocate a trial treatment, which will be prepared by the local participating Pharmacy.

Note that randomisation will be performed during normal office hours (09.00 – 17.00) and the trial allows for randomisation to occur months or years after the stroke. Therefore, in the event of computer failure (for example: server failure), the investigator will wait until the computer system is re-established to perform the randomisation. Normally, this would only be a few minutes to two hours. However, if this delay requires the patient to return on a separate occasion to collect the drugs, then a separate appointment will be made and the patient's travel expenses covered as per other trial visits.

Patients will be randomised to one of the following at a 1:1 ratio as per Section 6.2:

- cilostazol versus no cilostazol
- isosorbide mononitrate versus no isosorbide mononitrate

resulting in a partial factorial comparison of cilostazol versus isosorbide mononitrate versus both drugs versus neither drug.

The randomisation algorithm will allocate a unique identifier which will be used to label the treatment pack containing the appropriate drug made up for the patient and dispensed by the local Pharmacy.

Participants with an indication for or contraindication to one of the trial drugs may still be randomised to the other trial drug. If a participant allocated to both trial drugs develops a contraindication to one of the trial drugs after randomisation into the trial, then they should discontinue that drug but continue to take the other trial drug. If a patient has to discontinue either or both drugs, they should continue to be followed up in the trial as planned (unless they withdraw from the trial). Patients should remain in follow up until the end of the trial follow up period, even if they discontinue trial drug, unless they withdraw from the trial.

The study will be performed open label. Placebo tablets are not available and masking by encapsulation is too complicated and expensive. However, blinding of outcomes is important to obtain unbiased information about tolerability, safety and efficacy. As such, structured symptom questionnaires, ascertainment of clinical outcomes and MRI analysis will be collected by individuals blinded to treatment allocation.

Treatment Allocation: Participants randomised to start drug will be provided with their allocated drug/s after randomisation at the baseline visit, which they will start on the next day (i.e. day 1, week 1). In the event that there is any delay between receiving the trial tablets and starting the treatment, then the patient will receive a reminder telephone call shortly before the day that the medication should be commenced. The first day of treatment defines day 1, week 1.

Participants will be supplied with trial tablets from the hospital pharmacy to cover a maximum period of six months. Detailed dated instructions on dosing will be provided in the patient pack. The participant will be telephoned between the end of weeks 1 and 2 and between the end of weeks 3 and 4, and other times during drug introduction as necessary, as well as follow-up time points, to ensure they are taking the medication correctly.

Drugs will be provided in their packs as marketed and licensed (i.e unaltered) and dispensed by the participating hospital pharmacy under research protocols.

5.5.2 Emergency Unblinding Procedures

If the patient develops a contraindication to the trial medications, the medication should be stopped in line with SPC guidance. Similarly if the patient develops a definite indication for the trial medications the study medications should be stopped as per the SPC.

As this is an open label trial, there should be no need to unblind the allocated treatment. If some contraindication to cilostazol or ISMN develops after randomisation, the trial treatment should simply be stopped as per SPC guidance. If identification of the trial drug is considered necessary, the tablets can simply be examined and identified as cilostazol or ISMN as they will be dispensed in their licensed packaging.

5.5.3 Withdrawal of Study Participants

Participants may withdraw consent at any point. Where participants agree, we will retain the data collected up to the point of withdrawal for analysis. If the participant is willing, we will record the reason for withdrawal. Trial medication may be stopped at any time by the investigator or treating physician if deemed advisable. Stopping trial medication on its own does not necessarily equate with withdrawal from the trial, and follow-up procedures will continue unless the participant withdraws consent. If after randomisation into LACI-2, a participant subsequently is found to have a condition which would have made them ineligible for recruitment, these participants will be retained in the trial, at least for the purposes of follow-up, to protect the 'intention to treat' principle of analysis, and a decision for the continuation of the IMP will be made by the Principal Investigator with the participant or their relatives in the event of loss of capacity on the basis of safety. A recurrent event, such as stroke or other outcome, is not of itself a reason for withdrawal or discontinuation of IMP. The participant's wishes as to continuing trial medication and participation in the event that they lose capacity during the trial will be documented in the trial consent form.

6 INVESTIGATIONAL MEDICINAL PRODUCTS

6.1 STUDY DRUGS

The IMP is defined by the active substance only, therefore all authorised brands may be used. Oral cilostazol or isosorbide mononitrate slow release will be prescribed as per the brand available in the participating hospital pharmacy. Most isosorbide mononitrate preparations are slow release in the UK however, in pharmacies where slow release preparations of isosorbide mononitrate are not available, then non-slow release preparations may be used.

Several doses and brands of these drugs are marketed in the UK, examples are given below.

Oral Cilostazol 100mg tables, Generics [UK] Ltd t/a Mylan, Station Close, Potters Bar, Hertfordshire, EN6 1TL, United Kingdom. (http://www.mylan.co.uk)

Oral Isotard® 25 mg XL (Isosorbide Mononitrate) tablets, Kyowa Kirin Limited, 3 Galabank Business Park, Queen Street, Galashiels, TD1 1QH, United Kingdom. (http://www.kyowa-kirin.com/kkr/)

Isosorbide mononitrate (ISMN) 20mg, Pharmavit Ltd, 177 Bilton Road, Perivale, Greenford, Middlesex, UB6 7HQ (http://www.pharmavit.eu)

Cilostazol and ISMN are both licensed products for treatment of vascular diseases in Europe and the example summaries of the product characteristics are appended to this trial protocol.

6.1.1 Study Drug Identification

The IMP is defined by the active substance only, therefore all authorised brands may be used.

Cilostazol, generic, as 50mg or 100mg tablets.

Isosorbide mononitrate slow release, generic, for example as 25mg XL or 50mg XL tablets to the suggested target dose of 40-60mg daily.

Isosorbide mononitrate, generic, as 20mg tablets to the suggested target dose of 40-60mg daily.

Most isosorbide mononitrate preparations are slow release in the UK. However, where slow release preparations of isosorbide mononitrate are not available, then non-slow release preparations may be used, but the dose should be split half in the morning (e.g. 08.00 am) and half in the evening (e.g. 18.00hrs). Non-slow release preparations may only be available in 20mg tablets in which case the 20mg should be substituted for the 25mg dose. A target dose of ISMN is 40-60mg daily. Detailed prescribing and administration instructions will be provided in the treatment pack.

6.1.2 Study Drug Manufacturer

Details of one manufacturer of each of the trial drugs are given below. All drugs are available from several providers in the UK. Pharmacies may provide the brand of each drug that is available to them, i.e. a specific brand is not required for the trial. Example manufacturers are:

Oral Cilostazol 100mg tables are manufactured by Generics [UK] Ltd t/a Mylan, Station Close, Potters Bar, Hertfordshire, EN6 1TL, United Kingdom. (http://www.mylan.co.uk).

Oral Isotard® 25 mg XL (Isosorbide Mononitrate) tablets are manufactured by Kyona Kirin Limited, 3 Galabank Business Park, Queen Street, Galashiels, TD1 1QH, United Kingdom. (http://www.kyowa-kirin.com/kkr)

Isosorbide mononitrate (ISMN) 20mg are manufactured by Pharmavit Ltd, 177 Bilton Road, Perivale, Greenford, Middlesex, UB6 7HQ (http://www.pharmavit.eu)

6.1.3 Marketing Authorisation Holder

Details of one marketing authorisation holder of each of the trial drugs are given below.

Cilostazol 100mg tables - Generics [UK] Ltd t/a Mylan, Station Close, Potters Bar, Hertfordshire, EN6 1TL, United Kingdom, under marketing authorisation number PL 04569/1426.

Isotard® 25 mg XL tablets – Kyowa Kirin Limited, 3 Galabank Business Park, Queen Street, Galashiels, TD1 1QH, United Kingdom, under marketing authorisation number PL 16508/0018.

Isosorbide mononitrate (ISMN) 20mg tablets - Pharmavit Ltd, 177 Bilton Road, Perivale, Greenford, Middlesex, UB6 7HQ (http://www.pharmavit.eu) under marketing authorisation number PL 04556/0057..

6.1.4 Labelling and Packaging

The IMP will be clearly labelled for clinical trial use only. The participant's study ID number will be displayed on the treatment pack.

Each pack will be labelled in accordance with Annex 13 of Volume 4 of The Rules Governing Medicinal Products in the EU: Good Manufacturing Practices, with the primary and secondary packaging remain together throughout the trial.

Detailed prescribing and administration instructions will be provided with the treatment pack. Dose initiation in first 2-4 weeks will be guided by a regular phone calls and instructions.

6.1.5 Storage

The trial drugs will be stored in participating hospital pharmacies as per requirements for the branded products. They will be stored in a restricted access area where temperature is monitored and maintained below 25°C. Stability data exist which demonstrates that cilostazol is stable for 3 years without temperature restrictions. ISMN is stable for 3 years at temperatures below 25°C.

6.2 DOSING REGIME

Patients will be supplied with trial drug in its usual (marketing) packaging unaltered. Patients will be issued with instructions reflecting the allocated dosing schedule which will instruct them what tablets they have to take initially and how to increase the dose. They will receive a phone call after 1 to 2 and 3 to 4 weeks as per schedule below to guide dose escalation. If a patient encounters intolerable side effects they will be asked to return to the highest previously tolerated dose and this will be recorded in the eCRF and hospital notes. They will be given clear instructions by phone or in person (depending on the stage of the trial). Patients will also receive instruction on how they should decrease the dose of trial drug incrementally at the end of the study.

Table 1: Patients randomised to Isosorbide Mononitrate alone - either XL or non-XL preparations, example

If a slow release preparation is not available, then a non-slow release preparation may be used, but the dose should be given half in the morning (eg 08.00 am) and half in the evening (eg 18.00hrs)

ISMN XL Dosing Regime		
**Day**	**ISMN XL 25mg**	
	**Morning**	**Evening**
1-5	25mg	nil
6-10	50mg	nil
11-15	50mg	nil
16-20	50mg	nil
**At end of study**		
**Week**	**Morning**	**Evening**
53	25mg	Nil
54	Nil	Nil

ISMN non-XL Dosing Regime		
**Day**	**ISMN non-XL 20mg**	
	**Morning**	**Evening**
1-5	nil	20mg
6-10	20mg	20mg
11-15	20mg	20mg
16-20	20mg	20mg
**At end of study**		
**Week**	**Morning**	**Evening**
53	20mg	Nil
54	Nil	Nil

Table 2: Patients randomised to cilostazol alone

Cilostazol Dosing Regime		
**Day**	**Cilostazol**	
	**Morning**	**Evening**
1-5	Nil	50mg
6-10	50mg	50mg
11-15	50mg	100mg
16-20	100mg	100mg
**At end of study**		
**Week**	**Morning**	**Evening**
53	50mg	50mg
54	Nil	Nil

Table 3: Patients randomised to both Isosorbide Mononitrate and Cilostazol

ISMN XL/Cilostazol Dosing Regime				
**Day**	**Isosorbide Mononitrate XL 25mg**		**Cilostazol**	
	**Morning**	**Evening**	**Morning**	**Evening**
1-5	25mg	nil	Nil	Nil
6-10	50mg	nil	Nil	Nil
11-15	50mg	nil	Nil	50mg
16- 20	50mg	nil	50mg	50mg
21-25	50mg	nil	50mg	100mg
26-30	50mg	nil	100mg	100mg
**At end of study**				
**Week**	**Morning**	**Evening**	**Morning**	**Evening**
53	25mg	Nil	50mg	50mg
54	Nil	Nil	Nil	Nil

ISMN non-XL/Cilostazol Dosing Regime				
**Day**	**Isosorbide Mononitrate 20mg**		**Cilostazol**	
	**Morning**	**Evening**	**Morning**	**Evening**
1-5	nil	20mg	Nil	Nil
6-10	20mg	20mg	Nil	Nil
11-15	20mg	20mg	Nil	50mg
16- 20	20mg	20mg	50mg	50mg
21-25	20mg	20mg	50mg	100mg
26-30	20mg	20mg	100mg	100mg
**At end of study**				
**Week**	**Morning**	**Evening**	**Morning**	**Evening**
53	20mg	Nil	50mg	50mg
54	Nil	Nil	Nil	Nil

Table 4: Patients randomised to neither Isosorbide Mononitrate or Cilostazol

Neither ISMN/Cilostazol Dosing Regime				
**Day**	**Isosorbide Mononitrate**		**Cilostazol**	
	**Morning**	**Evening**	**Morning**	**Evening**
1-4	Nil	Nil	Nil	Nil
5-8	Nil	Nil	Nil	Nil
9-12	Nil	Nil	Nil	Nil
13, 14	Nil	Nil	Nil	Nil
**At end of study**				
**Week**	**Morning**	**Evening**	**Morning**	**Evening**
53	Nil	Nil	Nil	Nil
54	Nil	Nil	Nil	Nil

6.3 DOSE CHANGES

Doses will be initiated as per the example regime in section 6.2. Patients will be allowed to increment the dose more slowly, or to stay at a previously tolerated dose where their symptoms preclude reaching the target dose stated in 6.2. Variation of dose like this will not count as a protocol deviation. Patients will be able to stay on the dose they can tolerate. If necessary, this will be done under close guidance of the researcher. There will be no other changes to the doses described in section 6.2.

6.4 PARTICIPANT COMPLIANCE

Adherence and the tolerability of these treatments is a key outcome measure as part of the trial feasibility assessment. Symptoms related to taking either drug will be assessed using a structured questionnaire given as per schedule and the patient will be asked if they are continuing to take the medication at each review of symptoms.
As indicated in 7.1 below, during the start-up phase, patients will be asked to contact the local PI or delegated research nurse if they experience adverse symptoms, or trial help line should neither of the above be available. Once patients are established on their steady dose of tablets, they will be asked to inform the trial team via the help line or contact their local PI if they experience untoward symptoms (in addition to contacting their GP or other relevant hospital service). Such episodes of contact will be recorded in the eCRF or AE form or reported as SAEs as appropriate.

Patients will be asked to return any unused tablets to the hospital pharmacy that provided the tablets at six and 12 months. Pharmacy will submit a tablet count to the eCRF.

6.5 OVERDOSE

Cilostazol: The SPC for Cilostazol states that there is limited information on the effects of acute overdose in humans. It is anticipated to feature severe headache, diarrhoea, tachycardia and possibly cardiac arrhythmia. Management would be supportive care and gastric lavage as appropriate.

Isosorbide Mononitrate: The SPC describes the expected effects of isosorbide mononitrate in overdose and details measures for management.

The risk of overdose will be minimised by excluding patients with a history of overdose or suicide, or significant active mental health problems.

6.6 OTHER MEDICATIONS

6.6.1 Non-Investigational Medicinal Products

Not applicable

6.6.2 Permitted Medications

Patients may continue to take all usual prescribed medication during the study except those listed in 6.6.3 and in the exclusion criteria.

6.6.3 Prohibited Medications

Isosorbide mononitrate:

Phosphodiesterase 5’ inhibitors (tadalafil, sildenafil, vardenafil).

Cilostazol:

Other strong inhibitors of metabolic enzymes CYP3A4 or CYP2C19 (e.g. diltiazem).
Erythromycin, clarithromycin, ketoconazole, itraconazole, omeprazole
Dual antiplatelet drugs (e.g. aspirin and clopidogrel simultaneously)
Anticoagulants (warfarin, heparin, dabigatran, rivaroxaban, apixaban)

Although not noted in the BNF, SPCs for cilostazol indicate caution is advised with other strong inhibitors of metabolic enzymes CYP3A4 or CYP2C19, such as simvastatin, atorvastatin,

lovastatin, carbamazepine, phenytoin, rifampicin. BNF notes caution with isosorbide mononitrate and hypotensive agents such as diltiazem, hydralazine, etc.
Please refer to the SPCs for full details.

7 STUDY ASSESSMENTS

7.1 SAFETY ASSESSMENTS

Patients will be encouraged to contact the Trial team if, after randomisation, they have any concerns at all about the study drugs, their stroke, if they develop symptoms suggestive of a recurrent vascular event, if they become cognitively impaired or have another medical problem.

Patients will be assessed at 1-2-weekly intervals in the first month and six monthly intervals as per Study Flow diagram and Section 7.2 for the presence of any symptoms related to taking the trial drugs. This will include a structured questionnaire to assess for headache, dizziness, palpitations, bleeding, recurrent neurological or cardiac symptoms as outcome events that are also common after stroke, and adherence to medication.

7.2 STUDY ASSESSMENTS

Study assessments are shown in **Table 5** below, and in **Figure 2**.

Table 5: Study Assessments

Assessment	Prior to Baseline	Visit 1 Baseline	Week 1-2 FU	Week 3-4 FU	6 month FU	12 month FU
Screening for eligibility and Consent[1];	X^{S}					
Confirm and document ongoing consent		X^{S}				
Medical including drug history		X^{S}				
Assess MR or CT diagnostic scan and send a copy to Edinburgh		X^{S}				
Randomisation		X^{S}				
Haematology (full blood count) and Biochemistry (urea, electrolytes, creatinine) – most recent value obtained since time of index stroke is acceptable unless clinical reason to expect change		X^{S}				
Blood Pressure recorded		X^{S}				X^{S,*}
Cognitive test: document years of education; Montreal Cognitive Assessment (MOCA)		X^{S}				
Timed Trail Making Test B		X^{S}				X^{S,*}
Dispense trial medication[2]		X^{S}			X^{S}	
Structured questionnaire: symptoms; medication history and IMP tablet adherence			X^{S}	X^{S}	X^{C}	X^{C}
Structured Q: recurrent vascular events, mRS, TICS, TMOCA, SIS, ZUNG					X^{C}	X^{C}
Covid questionnaire		X^{S}	X^{S}	X^{S}	X^{C}	X^{C}
Obtain IQCODE (post/phone) from relative						X^{C}
Follow-up brain MRI						X^{S}
Health Economics data: EQ-5D-5L, EQ-VAS						X^{C}
Adverse event / con meds reporting as necessary			X^{S}	X^{S}	X^{S,C}	X^{S,C}

[1]Consent will be obtained before the data collection procedures commence or randomisation is performed. Randomisation occurs at the end of the baseline visit. *at 12 months in some centres only. [2] Dispensing in 3-monthly intervals is allowed. [S]Assessment performed by local site team. [C]Assessment performed by blinded assessor who is part of the central trial team.

SIS=Stroke Impact Scale, TICS= Telephone Interview for Cognitive Status, TMOCA= telephone MOCA.

Medical history: Will be performed by the study researcher and will include details of incident stroke, past medical history, vascular risk factors, medication history and years of education.

Baseline brain MR (preferred wherever possible) or CT: To identify the index stroke or exclude other causes of symptoms, and assess the burden of SVD – this is the clinical scan with T2, FLAIR, T1, T2* and diffusion imaging or CT obtained soon after the presentation with stroke that shows either:
- a recent, relevant (in time and location) acute lacunar infarct,
- or, if no visible acute lacunar infarct, there is no competing pathology as a cause for stroke (e.g. no acute cortical infarct, no acute lacunar or cortical intra-cerebral haemorrhage, no stroke mimic such as tumour, subdural haematoma). Scans will be sent for central reading.

Cognitive baseline testing: Patients will be assessed at baseline using the Montreal Cognitive Assessment (MoCA), the timed Trail Making Test B for processing speed/executive function, and ZUNG for mood. Their years in full time education will be recorded.

Symptoms and adverse effects: Will be assessed using a structured validated questionnaire to assess for recurrent neurological symptoms and whether these meet clinical criteria for TIA or stroke, angina or MI, peripheral vascular disease (and details thereof), headache, dizziness, palpitations, altered bowel habit, and adherence to medication, and if any other symptoms have occurred. The patient will be asked whether were severe enough to interfere with normal daily activities. Patients will also be able to report any other symptoms and describe them in detail. Depending on the time point in the study this will either be completed face to face at a study visit or by telephone.

Tablet adherence: Will be assessed by asking the patient about their medication usage and recording the details in the eCRF. In addition, patients will be asked to return any packs of unused tablets to the hospital pharmacy at the end of the trial. All returned tablets will be counted at Pharmacy and then be destroyed.

Blood pressure: Will be assessed using a validated clinical instrument that meets British Hypertension Society standards.

Haematology and biochemistry: Full blood count, urea and electrolytes and renal function will be obtained from the most recent sample obtained since the time of the index stroke. If there is a clinical reason to expect change in which case the full blood count and biochemistry should be repeated prior to randomisation. These samples will be analysed in the hospital haematology or biochemistry labs in the centre in which the patient has been recruited.

Neurological outcomes: Information on recurrent stroke, TIA, angina, MI, PVD will be assessed by postal or phone questionnaire at 6 and 12 months. Patients will be encouraged to seek medical advice if they develop new neurological events during the trial.

Functional outcome: modified Rankin Scale to assess dependency at 6 and 12 months. If participants are unable to be contacted, the central assessor will obtain the most recent modified Rankin Score from the local staff (if available).

Cognitive outcomes: Telephone MOCA, Telephone Interview for Cognitive Status (TICS), mobility and physical functioning sections of the Stroke Impact Scale and the ZUNG will be collected remotely at six and 12 months by the central trial team. TRAILS B will be repeated when attending for 1 year MR in Centres that are able to do this. TICS will be collected for consistency with prior and ongoing trials (ENOS, TARDIS, RIGHT 2, TICH) and good comparability.[62] TMOCA will be collected for comparison with TICS for potential use in future

trials as it purports to assess executive function more than TICS. IQCODE will be collected from the relative/partner/carer by post or phone.

Covid-19 status: Using a structured questionnaire, participants will be asked at each visit if they have had Covid-19 and, if so, how this was diagnosed and managed.

Health economics: EQ-5D-5L, EQ-VAS, return to work, hospitalisation, need for long term supportive care will be collected at 12 months.

Follow-up Brain MRI: all patients will have repeat brain MRI at 12 months to assess burden of WMH, microbleeds, lacunes, new infarcts or haemorrhage and atrophy and where feasible to assess diffusion tensor tissue parameters. These scans will be sent to Edinburgh for central blinded adjudication.

Follow-up at 6 and 12 months will be by post and telephone, blinded to allocated treatment.A trained assessor who is part of the central trial team and based either at the University of Edinburgh or University of Nottingham and who is blinded to treatment and baseline clinical information, will first confirm with the GP that the participant is contactable. They will then contact the patient or carer by post and phone to administer the questionnaires following a standardised script.

In addition to the assessments detailed above, participants will receive an informal contact at least once between 4 weeks and 6 months and 6 months and 12 months (e.g. a brief phone call or a participant newsletter). During those contacts, a member of the central or site teams will update participants on the trial status, thank them for their continued help with the study, and give them an opportunity to ask questions.

8 DATA COLLECTION

Please see section 7.2 above for measurements and time points.

The medical history, cognitive assessment, and structured questionnaire for symptoms and adverse events will be obtained from the patient in person (and medical notes where necessary) and by telephone by the study researcher after appropriate training as required and entered into the electronic case record form. These researchers will also measure blood pressure, obtain blood samples for haematology and biochemistry as necessary (analysed in the hospital NHS haematology or biochemistry labs in the centre in which the patient has been recruited).

The researcher at each site will co-ordinate the appointment for one year MRI. A reminder that the MRI appointment is due will be sent by the Trial Co-ordinating Centre. The MRI will be performed in approved radiology departments using sequences specified for the trial.

All baseline medical, cognitive, scanning and laboratory data will be entered into a secure password protected electronic case record form (eCRF) run from the University of Nottingham, accessible to approved research staff for data entry. The data from central reading of MRI will be added to the eCRF data for statistical analysis. Participant and relative/carer contact details, required to perform Trial follow-up, will be stored in an encrypted secure password-protected database also provided by the University of Nottingham, separate to the Trial eCRF database, and only accessible to selected researchers in the Trials Unit, University of Edinburgh and the University of Nottingham.

Paper versions of the CRF (i.e. source data sheet) will be available to assist with data collection when interviewing patients at baseline and during follow-up visits in phone or in person. Paper CRFs will be filed in the patient's paper-based folder and held in a secure locked filing cabinet at site.

The MR or CT brain images obtained at diagnosis of the stroke and 1 year follow-up MRI will be anonymised and sent to Edinburgh where they will be assessed and quantified for the index

stroke, features of small vessel disease and other features, using validated scores and volume measurements by an experienced rater. Images will only be identified by study ID.

Questionnaires will be checked for completeness and any queries resolved by phone. Telephone follow-up will be used to minimise missing data. All paper forms will be stored in a secure locked facility after data have been entered onto the eCRF.

9 STATISTICS AND DATA ANALYSIS

9.1 SAMPLE SIZE CALCULATION

There are few data on which to base sample size. There is little experience with cilostazol in Europe or North America, and little experience with ISMN in this group of patients in the UK (or in any patients with stroke). We have been guided by colleagues in Japan where cilostazol is in common use and there is experience with it in stroke. The main purposes of the trial are to assess tolerability and safety.

Table 6 Annual absolute risks (%) of outcome events after lacunar stroke

Vascular death	Non-vasc death	Non-fatal isch stroke or TIA	Non-fatal ICH	MI	MACE	Dependent (mRS 3-5)	Any cogn impt.	Dementia
1.8	0.5	2.5	0.5	0.6	3	15	30	15

We calculated mean annual event rates (Table 6) from trials (SPS3,[33] lacunar patients in ENOS[50] IST-3,[63] of cilostazol, Figure 1) and observational data (LADIS;[64] our[4, 65] and other[66] studies). We expect deaths including vascular deaths of median 2.0% p.a., with 4% being the upper 95% CI of 2% in 400 patients.[33] LACI-2 therefore has a sample size of 400.

For a main Phase III trial, recurrent stroke 2.5%pa, MI 0.6%pa and vascular death 1.8%pa,[4, 33] (MACE) are infrequent; new cognitive impairment c16% at one year,[6, 66] dependency 15%, are frequent and important to patients, justifying their inclusion in a composite primary outcome. However, these event rates are imprecise with wide ranges; more precise estimates are needed. We estimate main Phase III trial sample size at 1100 (Table 7), assuming 80% power, alpha 5%, 10% loss to follow-up, composite event rate (45%) and conservative estimate of cilostazol effect (20% RRR is the lower 95% CI of effect, Figure 1). A primary outcome including MRI SVD progression (A, below) was considered but at least 15% of patients miss repeat MRI, sample sizes were similar (or larger, due to variance in WMH change[65] short term), the cost is much higher and the WMH intermediary measure is less relevant to patients.

Table 7. Sample size for composite outcome in main trial, estimated event rates.

Composite model	A	B	Ci	Cii	D
Composite outcome for Phase III includes:	MACE, dementia, non-vasc death, new MR signs	MACE, dementia, death	MACE, cog↓, dependency↓, death		MACE, cog imp, depend, all death
1-beta (power)	80%	80%	80%	80%	80%
Event rate, control, pa	50%	10%	30%	30%	45%
Relative risk reduction	20%	20%	20%	30%	20%
Event rate, active, pa	40%	8%	24%	21%	36%
Total sample size	950	6626	1784	778	976
Total trial size, inc losses	1250	7400	2000	900	1100

Assume: 1:1 randomisation and Fleiss adjustment; alpha 5%; primary outcome incomplete in 10%

9.2 PROPOSED ANALYSES

We will compare cilostazol v no cilostazol, ISMN v no ISMN, cilostazol and ISMN v neither. The proportion completing to target dose and any adverse events will be assessed using odds ratios. The LACI-2 will focus on numbers recruited, retained, tolerance and safety outcomes, blinded for TSC and unblinded intention to treat (ITT) for DMC. Safety analysis will use: Kaplan-Meier and Cox proportional regression for analysis of time-to-event outcomes of death; binary logistic regression for SAEs, recurrent stroke, MI, and in those completing 12 month MRI, the amount of microbleeds, siderosis, new infarcts, WMH burden; all adjusted for minimisation variables to maximise power.[67] We will collect data for future health economics analysis (EQ-5D-5L, EQ-VAS).

A secondary ITT analysis will be performed using logistic regression adjusted for minimisation variables of cilostazol+/-ISMN versus control on efficacy and safety outcomes, with secondary tests of subgroup interactions. Ordinal logistic regression will be used for ordinal categorical outcomes (e.g. mRS) to increase power.[68]

A Statistical Analysis Plan (SAP) will be published prior to database lock and will describe analysis procedures and procedures for missing, unused or spurious data, and definitions of populations analysed.

10 ADVERSE EVENTS

Safety monitoring will only commence after the patient has been randomised into the trial.

The Investigator is responsible for the detection and documentation of events meeting the criteria and definitions detailed below. Full details of contraindications and side effects that have been reported following administration of the IMP can be found in the relevant Summary of Product Characteristics (SPC)/Investigator’s Brochure (IB).

10.1 DEFINITIONS

Adverse events will only be recorded/reported after the participant has been randomised into the trial.

An **adverse event** (AE) is any untoward medical occurrence in a clinical trial participant which does not necessarily have a causal relationship with an investigational medicinal product (IMP).

An **adverse reaction** (AR) is any untoward and unintended response to an IMP which is related to any dose administered to that participant.

A **serious adverse event** (SAE), **serious adverse reaction** (SAR). Any AE or AR that at any dose:

- results in death of the clinical trial participant;
- is life threatening*;
- requires in-patient hospitalisation^ or prolongation of existing hospitalisation;
- results in persistent or significant disability or incapacity;
- consists of a congenital anomaly or birth defect;
- results in any other significant medical event not meeting the criteria above.

*Life-threatening in the definition of an SAE or SAR refers to an event where the participant was at risk of death at the time of the event. It does not refer to an event which hypothetically might have caused death if it were more severe.

^Any hospitalisation that was planned prior to enrolment will not meet SAE criteria. Any hospitalisation that is planned post enrolment will not meet the SAE criteria unless it constitutes an untoward medical occurrence (e.g. cosmetic elective surgery, social and/or convenience admission, etc., will not count as SAEs).

A suspected unexpected serious adverse reaction (SUSAR) is any AR that is classified as serious and is suspected to be caused by the IMP, that it is not consistent with the information about the IMP in the Summary of Product Characteristics (SPC) or Investigators Brochure.

10.2 IDENTIFYING AEs AND SAEs

Participants will be asked about the occurrence of AEs/SAEs at each visit during the study. Participants will also be encouraged to contact the local PI or Central Trial Office (ECTU) via the Trial Helpline if they experience a potential adverse event. Open-ended and non-leading verbal questioning of the participant will be used to enquire about AE/SAE occurrence. Participants will also be asked if they have been admitted to hospital, had any accidents, used any new medicines or changed concomitant medication regimens. If there is any doubt as to whether a clinical observation is an AE, the event will be recorded.

AEs and SAEs may also be identified via information from GPs, other hospital specialties, and support departments e.g. laboratories, using usual procedures

10.3 RECORDING AEs AND SAEs

When an AE/SAE occurs, it is the responsibility of the Investigator, or another suitably qualified physician in the research team who is delegated to record and report AEs/SAEs, to review all documentation (e.g. hospital notes, laboratory and diagnostic reports) related to the event. The Investigator will then record all relevant information in the participant's medical records.

Information to be collected includes type of event, onset date, dose at the time, Investigator assessment of severity and causality, date of resolution as well as treatment required, investigations needed and outcome as per ACCORD requirements.

Depending on the type of the event and the seriousness and causality assessment, all relevant information will also be recorded on the eCRF, AE log, SAE form and/or eCRF as detailed below and summarised in Table 8. The flow chart in Figure 3 can be used by investigators to aid recording and reporting of safety events.

10.3.1 Pre-existing Medical Conditions

Pre-existing medical conditions (i.e. existed prior to informed consent) should be recorded as medical history and only recorded as AEs if medically judged to have unexpectedly worsened during the study.

10.3.2 Outcome Events

The following events are **outcome events** and will be recorded as such in the eCRF. All outcome events must be assessed for seriousness and causality by the PI or a delegated trial physician. Outcome measures are not recorded as AEs or further reported to the Sponsor, unless they fulfil the criteria of seriousness as defined in section 10.1 and are deemed possibly related to the IMP. The TSC will review all outcome events six monthly blinded to treatment allocation; the DMC will review all outcomes unblinded to treatment allocation annually and on special request. These events are not of themselves reasons to automatically discontinue the trial treatment, the need for which should be assessed by the PI and attending medical staff and decisions notified to the Trials Office. The participant's wishes in the event of loss of capacity are collected on the Trial Participant Consent Form which should also be consulted.

- Bleeding
- Bruising
- Chest pain, non-specific
- Diarrhoea
- Dizziness or light headedness
- Falls
- Headache
- Heart attack

- Heartburn
- Hypotension
- Loss of mental capacity
- Nausea
- Palpitations
- Recurrent stroke or TIA

10.3.3 Events That Are Common in the Trial Population

The primary event of stroke is classified as a pre-existing condition. As such, the occurrence or expected progression of stroke related events are common. In addition, participants are likely to have many minor adverse events during the course of the study. These events will be recorded in the medical records and assessed as per section 10.4. However, they are not usually recorded as AEs/SAEs, unless they are thought to be caused by the participant's involvement in the trial.

- Accidents (domestic, traffic, occupational)
- Agitation
- Anxiety
- Asthma
- Atrial fibrillation or other cardiac dysrhythmia
- Bacteraemia
- Blepharospasm
- Breathlessness
- Bronchitis
- Bronchospasm
- Candida
- Carotid endarterectomy
- Chelitis
- Chest infection
- Cholecystitis, gall stones
- Chronic Obstructive Lung Disease or exacerbation thereof
- Common cold
- Constipation
- Deep vein thrombosis
- Dysphagia
- Eczema
- Electrolyte imbalance
- Embolism
- Fatigue
- Flu
- Gastrointestinal disturbance, non-specific
- Heart failure
- Heart valve or septal disorder
- Hernia
- Hypercalcaemia
- Hyperglycaemia
- Hypertension
- Hyperuricaemia
- Hypoxia
- Impetigo
- Incontinence, urinary or faecal
- Leucopenia
- Lymphadenopathy

- Malignancy, new diagnosis or new treatment for existing diagnosis
- Mild ankle swelling
- Mood disorders
- Muscle twitching,
- Oesophagitis
- Operations or medical procedures (endoscopy, pacemaker insertion)
- Oral ulceration
- Osteoarthritis
- Osteoporosis
- Other infections including soft tissue or skin infections and generalised sepsis
- Painful shoulder syndromes
- Pancreatitis,
- Periodontal disease
- Peripheral vascular disease
- Pleural effusions
- Pneumonia
- Pneumothorax
- Pressure sores
- Renal dysfunction
- Renal stones
- Sedation
- Seizure
- Sexual dysfunction
- Shingles
- Spasticity or contractures
- Spinal disc problem including spinal stenosis
- Thrombophlebitis
- Urinary catheterisation
- Urinary infections
- Varicose ulcers
- Vasculitis
- Venous thromboembolism
- Vertigo
- Visual Loss e.g. cataract or macular degeneration or retinal detachment
- Vomiting
- Weight loss
- Any other known complications of or symptoms suggestive of worsening stroke

10.3.4 All Other Events Not Covered Above

All events not covered by sections 10.3.2 or 10.3.3, including known IMP side effects, should be recorded as AEs/SAEs and assessed and reported as per sections 10.4 to 10.6.

Table 8: Summary of safety recording requirements.

	**Outcome event (listed in section 10.3.2)**	**Event common in stroke patients (listed in section 10.3.3)**	**All other events**
**Event not related to IMP(s), non-serious**	eCRF	Medical notes only	AE log
**Event not related to IMP(s), serious**	eCRF	Medical notes only	SAE report form
**Event potentially related to IMP(s), non-serious**	eCRF	AE log	AE log

**Event potentially related to IMP(s), serious**	SAE report form	SAE report form	SAE report form

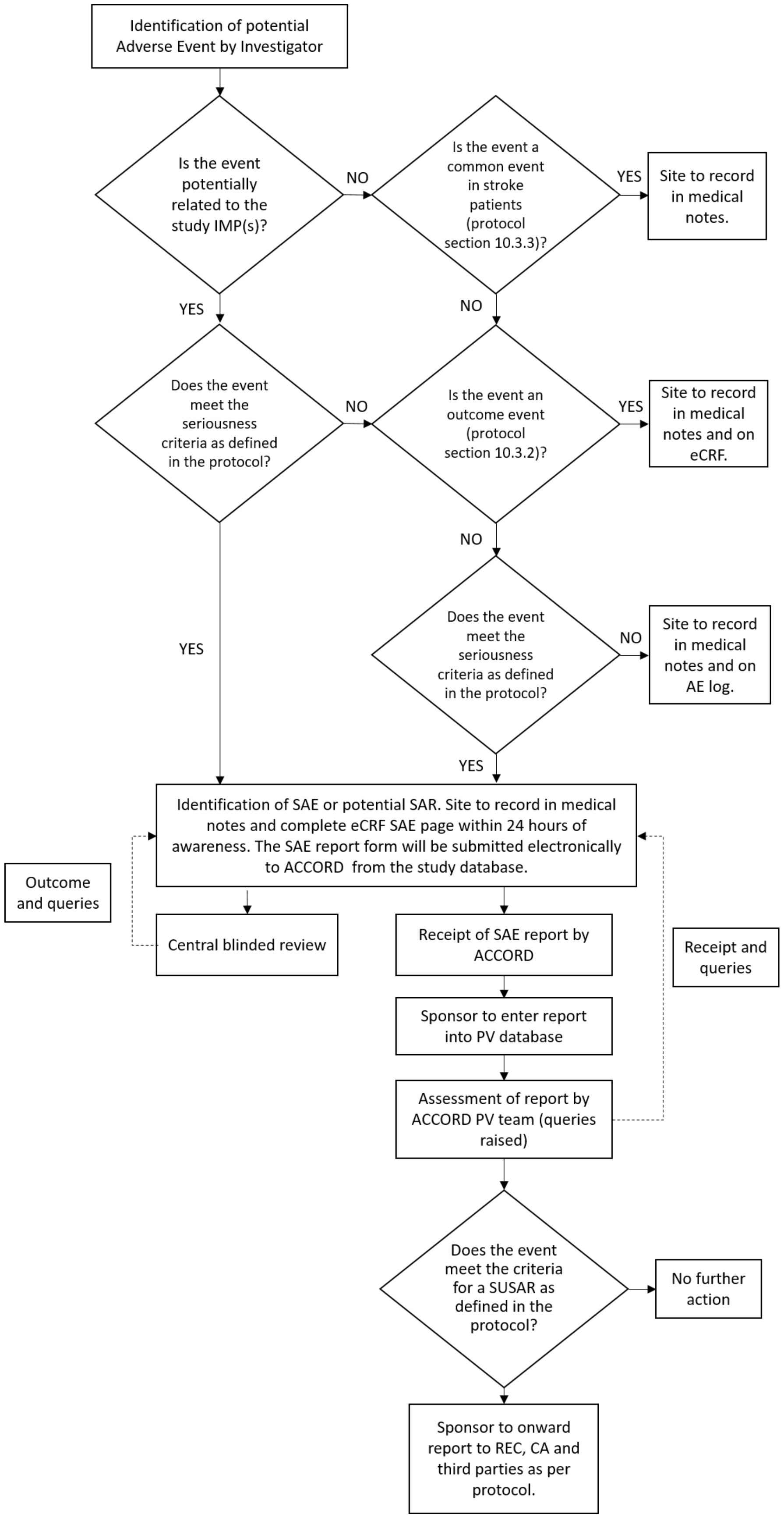

Figure 3: Flow diagram showing the process for recording and reporting of safety information.

10.4 ASSESSMENT OF AEs AND SAEs

Each AE must be assessed for seriousness, causality, severity and ARs must be assessed for expectedness by the Principal Investigator or another suitably qualified physician in the research team who has been delegated this role.

For randomised studies, AEs will be assessed as though the participant is taking active IMP. SUSARs will be unblinded by ACCORD before they are reported to the Research Ethics Committee (REC) and the Competent Authority (CA) (by ACCORD).

The Chief Investigator (CI) may not downgrade an event that has been assessed by an Investigator as an SAE or SUSAR, but can upgrade an AE to an SAE, SAR or SUSAR if appropriate.

10.4.1 Assessment of Seriousness

The Investigator will make an assessment of seriousness as defined in Section 10.1.

10.4.2 Assessment of Causality

The Investigator will make an assessment of whether the AE/SAE is likely to be related to the IMP according to the definitions below.

- Unrelated: where an event is not considered to be related to the IMP.

- Possibly Related: The nature of the event, the underlying medical condition, concomitant medication or temporal relationship make it possible that the AE has a causal relationship to the study drug. The assessment of causality will be made against the reference safety information found in the Summary of Product Characteristics.

Where non Investigational Medicinal Products (NIMPs) e.g. rescue/escape drugs are given: if the AE is considered to be related to an interaction between the IMP and the NIMP, or where the AE might be linked to either the IMP or the NIMP but cannot be clearly attributed to either one of these, the event will be considered as an AR. Alternative causes such as natural history of the underlying disease, other risk factors and the temporal relationship of the event to the treatment should be considered and investigated. The blind should not be broken for the purpose of making this assessment.

10.4.3 Assessment of Expectedness

If the event is an AR, the evaluation of expectedness will be made based on knowledge of the reaction and the relevant product information documented in the SPC/IB.

The event may be classed as either:

Expected: the AR is consistent with the toxicity of the IMP listed in the SPC/IB.

Unexpected: the AR is not consistent with the toxicity in the SPC/IB.

10.4.4 Assessment of Severity

The Investigator will make an assessment of severity for each AE/SAE and record this on the CRF or SAE form according to one of the following categories:

Mild: an event that is easily tolerated by the participant, causing minimal discomfort and not interfering with every day activities.

Moderate: an event that is sufficiently discomforting to interfere with normal everyday activities.

Severe: an event that prevents normal everyday activities.

Note: the term 'severe', used to describe the intensity, should not be confused with 'serious' which is a regulatory definition based on participant/event outcome or action criteria. For

example, a headache may be severe but not serious, while a minor stroke is serious but may not be severe.

10.5 REPORTING OF AEs TO THE SPONSOR

Adverse events occurring during the trial that are observed by the investigator or reported by the participant should be recorded in the patient's medical records.

Only SAEs/SARs/SUSARs will be documented in the eCRF and reported expeditiously to the Sponsor as below.

10.6 REPORTING OF SAEs/SARs/SUSARs

Once the Investigator becomes aware that an SAE has occurred in a study participant, the information will be reported to the ACCORD Research Governance & QA Office **immediately or within 24 hours**. If the Investigator does not have all information regarding an SAE, they should not wait for this additional information before notifying ACCORD. The SAE report form can be updated when the additional information is received. The SAE report will be submitted simultaneously to the CI.

The SAE report will provide an assessment of causality and expectedness at the time of the initial report to ACCORD according to Sections 10.4.2, Assessment of Causality and 10.4.3, Assessment of Expectedness.

The SAE report will be submitted electronically to the ACCORD Research Governance & QA Office and the CI directly from the study database. If the database is inaccessible then a paper copy of the SAE form should be completed and be transmitted by fax to ACCORD on **+44 (0)131 242 9447** or may be transmitted by hand to the office or submitted via email to safety@accord.scot. Only forms in a pdf format will be accepted by ACCORD via email.

Where missing information has not been sent to ACCORD after an initial report, ACCORD will contact the Investigator and request the missing information. The Investigator must respond to these requests in a timely manner.

All reports will be retained by the Investigator in the Investigator Site File (ISF).

10.7 REGULATORY REPORTING REQUIREMENTS

The ACCORD Research Governance & QA Office is responsible for Pharmacovigilance reporting on behalf of the co-sponsors (The University of Edinburgh and Lothian Health Board).

The ACCORD Research Governance & QA Office has a legal responsibility to notify the regulatory competent authority and relevant ethics committee (REC that approved the trial). Fatal or life threatening SUSARs will be reported no later than 7 calendar days and all other SUSARs will be reported no later than 15 calendar days after ACCORD is first aware of the reaction.

ACCORD, unless otherwise delegated, will inform Investigators at participating sites of all SUSARs and any other arising safety information.

ACCORD will be responsible for providing safety line listings and assistance; however, it is the responsibility of the Investigator to prepare the Development Safety Update Report. This annual report lists all SARs and SUSARs reported during that time period. The responsibility of submitting the Development Safety Update Report to the regulatory authority and RECs, lies with ACCORD.

10.8 CENTRAL BLINDED REVIEW OF SAEs

To ensure that collected safety data is reliable and robust, a central blinded assessor will evaluate all investigator-reported SAEs. This reviewer will have access to the web-based SAE form once this has been reported by the local investigator and submitted to ACCORD. The independent reviewer will assess the event diagnosis, expectedness of event and likely

causality, blinded to treatment allocation. If required, the reviewer will be able to request further information from the specific trial sites. The outcome of the central blinded review will be made available to local investigators for information. In case of discrepancies, the investigator's assessment as reported to ACCORD will comprise the definitive safety data for this trial and will be used for all analyses. Should an investigator change their assessment based on the outcome of the central blinded review, they will update the SAE record and submit a follow-up report to ACCORD.

10.9 FOLLOW UP PROCEDURES

After initially recording an AE or recording and reporting an SAE, the Investigator should make every effort to follow each event until a final outcome can be recorded or reported as necessary. Follow up information on an SAE will be reported to the ACCORD office.

If, after follow up, resolution of an event cannot be established, an explanation should be recorded on the CRF or AE log or additional information section of the SAE form. Adverse events will continue to be recorded for 1 month after the last dose of IMP.

11 PREGNANCY

Although pregnancy is not considered an AE or SAE, as a matter of safety, the Investigator will be required to record any female participant's pregnancy or any pregnancy of a female partner of a male participant, who became pregnant while participating in the study. The Investigator will need to record the information on a Pregnancy Notification Form and submit this to the ACCORD office within 14 days of being made aware of the pregnancy.

All pregnant female participants and pregnant partners of male participants will be followed up until the outcome of the pregnancy.

12 TRIAL MANAGEMENT AND OVERSIGHT ARRANGEMENTS

12.1 TRIAL MANAGEMENT GROUP

The trial will be coordinated by a Trial Management Group, consisting of the grant holders Professor Joanna Wardlaw (Chief Investigator), Professor Philip Bath (Deputy Chief Investigator, Principal Investigator in Nottingham), Dr Fergus Doubal (Principal Investigator, Edinburgh), Dr Niki Sprigg (Co-Investigator, Nottingham), Anna Heye, Trial Manager (Edinburgh), clinical research fellow (Edinburgh and Nottingham where relevant), trial co-ordinator (Nottingham and Edinburgh), trial statistician (Nottingham), Trial programmer (Nottingham), the Trial Image Data Manager (Edinburgh) and other Trial administrative and data management staff as appropriate.

The Trial Co-ordinator/Manager will oversee the day-to-day running of the study and will be accountable to the Chief Investigator. The Trial Manager will be responsible for checking the CRFs for completeness, plausibility and consistency. Any queries will be resolved by the Investigator or delegated member of the trial team. The Trial Co-ordinator in Edinburgh will cross check a sample of eCRFs of patients recruited in Nottingham and the trial coordinator in Nottingham will cross check a sample of eCRFs of patients recruited in Edinburgh.
A Delegation Log will be prepared for each site, detailing the responsibilities of each member of staff working on the trial.

12.2 TRIAL STEERING COMMITTEE

A Trial Steering Committee (TSC) has been established to oversee the conduct and progress of the trial. The current members may change as the study progresses: changes necessitated by illness or death or relocation of TSC members will not constitute a Protocol Deviation or Violation and will be addressed as a minor amendment. The roles and responsibilities of the TSC will be defined in the TSC Charter.

12.3 DATA MONITORING COMMITTEE

An independent Data Monitoring Committee (DMC), consisting of a Chair, two senior academic clinicians familiar with stroke and imaging and a statistician has been established to oversee the safety of participants in the trial. The current members may change as the study progresses: changes necessitated by illness or death or relocation of TSC members will not constitute a Protocol Deviation or Violation and will be addressed as a minor amendment. The roles and responsibilities of the DMC will be defined in the DMC Charter.

12.4 INSPECTION OF RECORDS

Investigators and institutions involved in the study will permit trial related monitoring and audits on behalf of the sponsor, REC review, and regulatory inspection(s). In the event of an audit or monitoring, the Investigator agrees to allow the representatives of the sponsor direct access to all study records and source documentation. In the event of regulatory inspection, the Investigator agrees to allow inspectors direct access to all study records and source documentation.

12.5 RISK ASSESSMENT

An independent risk assessment will be performed by an ACCORD Clinical Trials Monitor to determine if monitoring is required and if so, at what level. An independent risk assessment will also be carried out by the ACCORD Quality Assurance Group to determine if an audit should be performed before/during/after the study and if so, at what locations and at what frequency.

12.6 STUDY MONITORING AND AUDIT

An ACCORD Clinical Trials Monitor or an appointed monitor will contact the Investigator site prior to the start of the study and during the course of the study if required, in accordance with the monitoring plan, if required. Risk assessment will determine if audit, by the ACCORD QA group, is required. Details will be captured in an audit plan. Audit of Investigator sites, study management activities and study collaborative units, facilities and 3rd parties may be performed.

13 GOOD CLINICAL PRACTICE

13.1 ETHICAL CONDUCT

The study will be conducted in accordance with the principles of the International Conference on Harmonisation Tripartite Guideline for Good Clinical Practice (ICH GCP).

A favorable ethical opinion will be obtained from the HRA REC and local R&D approval will be obtained prior to commencement of the study.

13.2 REGULATORY COMPLIANCE

The study will not commence until a Clinical Trial Authorisation (CTA) is obtained from the appropriate Regulatory Authority. The protocol and study conduct will comply with the Medicines for Human Use (Clinical Trials) Regulations 2004, as amended.

13.3 INVESTIGATOR RESPONSIBILITIES

The Investigator is responsible for the overall conduct of the study at the site and compliance with the protocol and any protocol amendments. In accordance with the principles of ICH GCP, the following areas listed in this section are also the responsibility of the Investigator. Responsibilities may be delegated to an appropriate member of study site staff.

13.3.1 Informed Consent

The Investigator is responsible for ensuring informed consent is obtained before any protocol specific procedures are carried out. The decision of a participant to participate in clinical research is voluntary and should be based on a clear understanding of what is involved.

Participants must receive adequate oral and written information using the approved Participant Information and Informed Consent Forms. The oral explanation to the participant will be performed by the Investigator or qualified delegated person, and will cover all the elements specified in the Participant Information Sheet and Consent Form.

The participant must be given every opportunity to clarify any points they do not understand and, if necessary, ask for more information. The participant must be given sufficient time to consider the information provided. It should be emphasised that the participant may withdraw their consent to participate at any time without loss of benefits to which they otherwise would be entitled.

The participant will be informed and agree to their medical records being inspected by regulatory authorities and representatives of the sponsor(s) but understand that their name will not be disclosed outside the hospital.

The Investigator or delegated member of the trial team and the participant will sign and date the Informed Consent Form(s) to confirm that consent has been obtained. The participant will receive a copy of this document and a copy filed in the Investigator Site File (ISF) and participant's medical notes (a PDF version will be uploaded to the participant medical record at sites where records are all electronic).

13.3.2 Study Site Staff

The Investigator must be familiar with the IMP, protocol and the study requirements. It is the Investigator's responsibility to ensure that all staff assisting with the study are adequately informed about the IMP, protocol and their trial related duties.

13.3.3 Data Recording

The Principal Investigator is responsible for the quality of the data recorded in the eCRF at each Investigator Site. The source data plan identifies which source data correspond to eCRF data and states which data are recorded directly into the eCRF.

13.3.4 Investigator Documentation

Prior to beginning the study, each Investigator will be asked to provide particular essential documents to the ACCORD Research Governance & QA Office, including but not limited to:

- An original signed Investigator's Declaration (as part of the Clinical Trial Agreement documents);
- Curriculum vitae (CV) signed and dated by the Investigator indicating that it is accurate and current.

The ACCORD Research Governance & QA Office will ensure all other documents required by ICH GCP are retained in a Trial Master File (TMF), where required, and that appropriate documentation is available in local ISFs.

13.3.5 GCP Training

All study staff must hold evidence of appropriate GCP training.

13.3.6 Confidentiality

All laboratory specimens, evaluation forms, reports, and other records must be identified in a manner designed to maintain participant confidentiality. All records must be kept in a secure storage area with limited access. Clinical information will not be released without the written permission of the participant. The Investigator and study site staff involved with this study may not disclose or use for any purpose other than performance of the study, any data, record, or other unpublished, confidential information disclosed to those individuals for the purpose of the study. Prior written agreement from the sponsor or its designee must be obtained for the disclosure of any said confidential information to other parties.

13.3.7 Data Protection

All Investigators and study site staff involved with this study must comply with the requirements of the appropriate data protection legislation (including the General Data Protection Regulation and Data Protection Act) with regard to the collection, storage, processing and disclosure of personal information. Access to collated identifiable participant data will be restricted to individuals from the research team treating the participants, representatives of the sponsor(s) and representatives of regulatory authorities.

Computers used to collate the data will have limited access measures via user names and passwords.

Published results will not contain any personal data that could allow identification of individual participants.

14 STUDY CONDUCT RESPONSIBILITIES

14.1 PROTOCOL AMENDMENTS

Any changes in research activity, except those necessary to remove an apparent, immediate hazard to the participant in the case of an urgent safety measure, must be reviewed and approved by the Chief Investigator.

Amendments to the protocol must be submitted in writing to the appropriate REC, Regulatory Authority and local R&D for approval prior to participants being enrolled into an amended protocol.

14.2 PROTOCOL VIOLATIONS AND DEVIATIONS

Prospective protocol deviations, i.e. protocol waivers, will not be approved by the sponsors and therefore will not be implemented, except where necessary to eliminate an immediate hazard to study participants. If this necessitates a subsequent protocol amendment, this should be submitted to the REC, Regulatory Authority and local R&D for review and approval if appropriate.

Protocol deviations will be recorded in a protocol deviation log and logs will be submitted to the sponsors every 3 months. Each protocol violation will be reported to the sponsor within 24 hours of becoming aware of the violation.

Protocol violation: A protocol violation is a deviation that may potentially significantly impact the completeness, accuracy, and/or reliability of the study data or that may significantly affect a subject’s rights, safety, or well-being.

As tolerability of trial IMP is an outcome and will be recorded in the eCRF at each study visit, therefore a participant’s inability to tolerate the allocated IMP (ie not able to take target dose or even any of the dose) will not constitute a protocol deviation or violation.

14.3 SERIOUS BREACH REQUIREMENTS

A serious breach is a breach which is likely to effect to a significant degree:

(a) the safety or physical or mental integrity of the participants of the trial; or
(b) the scientific value of the trial.

If a potential serious breach is identified by the Chief investigator, Principal Investigator or delegates, the co-sponsors (QA@accord.scot) must be notified within 24 hours. It is the responsibility of the co-sponsors to assess the impact of the breach on the scientific value of the trial, to determine whether the incident constitutes a serious breach and report to regulatory authorities and research ethics committees as necessary.

14.4 STUDY RECORD RETENTION

As there is limited data on the use of isosorbide mononitrate and cilostazol in UK stroke patients all study documentation including personal linked data will be kept for a minimum of 5 years from the protocol defined end of study point. This is in case of any later events and also because of sponsor guidelines. Study documentation will not be destroyed without permission from the sponsor.

14.5 END OF STUDY

The end of study is defined as the completion of analysis of the study data.

The TSC and/or the co-sponsor(s) have the right at any time to terminate the study for clinical or administrative reasons, e.g. on recommendation of the DMC.

The end of the study will be reported to the Sponsor, REC and Regulatory Authority within 90 days, or 15 days if the study is terminated prematurely. The Investigators will inform participants of the premature study closure and ensure that the appropriate follow up is arranged for all participants involved.

A final clinical study report of the study will be provided to the REC and Sponsor within 1 year of the end of the study. The trial summary results will be uploaded to the European EudraCT trial entry and notified to MHRA as per ACCORD SOP CR009.

14.6 CONTINUATION OF DRUG FOLLOWING THE END OF STUDY

This is an early phase III trial to assess tolerability and safety of the trial medications in stroke patients taking routine post stroke secondary prevention medications. As such the trial drugs will not be continued beyond the study period as, at present, there is no evidence of efficacy in addition to standard post stroke care.

14.7 INSURANCE AND INDEMNITY

The co-sponsors are responsible for ensuring proper provision has been made for insurance or indemnity to cover their liability and the liability of the Chief Investigator and staff.
The following arrangements are in place to fulfil the co-sponsors' responsibilities:

- The Protocol has been designed by the Chief Investigator and researchers employed by the University and collaborators. The University has insurance in place (which includes no-fault compensation) for negligent harm caused by poor protocol design by the Chief Investigator and researchers employed by the University.
- Sites participating in the study will be liable for clinical negligence and other negligent harm to individuals taking part in the study and covered by the duty of care owed to them by the sites concerned. The co-sponsors require individual sites participating in the study to arrange for their own insurance or indemnity in respect of these liabilities.

- Sites which are part of the United Kingdom's Nation Health Service will have the benefit of NHS Indemnity.
- The manufacturer supplying IMP has accepted limited liability related to the manufacturing and original packaging of the study drug and to the losses, damages, claims or liabilities incurred by study participants based on known or unknown Adverse Events which arise out of the manufacturing and original packaging of the study drug, but not where there is any modification to the study drug (including without limitation re-packaging and blinding).

15 REPORTING, PUBLICATIONS AND NOTIFICATION OF RESULTS

15.1 AUTHORSHIP POLICY

Ownership of the data arising from this study resides with the study team. On completion of the study, the study data will be analysed and tabulated, and a clinical study report will be prepared for publication in a peer reviewed journal in accordance with ICH guidelines. The paper will be published by named members of the trial team on behalf of the Lacunar Intervention (LACI) 2 Trial (LACI-2). Members of the collaborative group will be listed in the publication. A report will be submitted to the funder (BHF UK). Papers describing secondary analyses will also be published and the data will be contributed to a systematic review where relevant. Any secondary publication may be published by named individuals, but with appropriate acknowledgement of the collaborative group.

15.2 PUBLICATION

The clinical study report will be used for publication and presentation at scientific meetings on stroke and dementia such as UK Stroke Forum, European Stroke Organisation Conference, International Stroke Conference, the World Stroke Congress, and conferences on Alzheimer’s disease and dementia. Investigators have the right to publish orally or in writing the results of the study. Reporting will be in compliance with CONSORT.

Summaries of results will also be made available to all Investigators for dissemination within their clinics (where appropriate and according to their discretion).

A newsletter will be sent to the participants informing them of the results and of other information relevant to small vessel disease and general information about maintaining a healthy lifestyle.

15.3 PEER REVIEW

The trial design was informed by a Stroke Research Network-funded NIHR Stroke Research Network Writing Workshop, held in Nottingham, 31 March 2014 and attended by 20 experts on small vessel disease, stroke, dementia and imaging. The workshop proposal underwent peer review prior to securing funding.

The trial underwent peer review during the funding application to the BHF.

A paper describing potential drugs to prevent SVD progression was peer reviewed and is now published in the International Journal of Stroke.[16]

The concepts described in this protocol have been presented at several Stroke and Dementia conferences and discussed.

The Stroke Research Network Prevention Studies Group reviewed the proposal in 2014 and supported the work.

Many UK SRN Centers have expressed interest in joining the trial.

16 REFERENCES

(1) Iadecola C. The pathobiology of vascular dementia. *Neuron* 2013;80:844-66.

(2) Wardlaw JM, Smith C, Dichgans M. Mechanisms of sporadic cerebral small vessel disease: insights from neuroimaging. *Lancet Neurol* 2013;12:483-97.

(3) Brayne C, Richardson K, Matthews FE, Fleming J, Hunter S, Xuereb JH et al. Neuropathological correlates of dementia in over-80-year-old brain donors from the population-based Cambridge City over-75s Cohort (CC75C) study. *J Alzheimers Dis* 2009;18:645-58.

(4) Jackson CA, Hutchison A, Dennis MS, Wardlaw JM, Lewis SC, Sudlow CL. Differences between ischemic stroke subtypes in vascular outcomes support a distinct lacunar ischemic stroke arteriopathy. A prospective, hospital-based study. *Stroke* 2009;40:3679-84.

(5) Jacova C, Pearce LA, Costello R, McClure LA, Holliday SL, Hart RG et al. Cognitive impairment in lacunar strokes: the SPS3 trial. *Ann Neurol* 2012;72:351-62.

(6) Makin S, Turpin S, Dennis M, Wardlaw J. Cognitive impairment after lacunar stroke: systematic review and meta-analysis of incidence, prevalence and comparison with other stroke sub-types. *J Neurol Neurosurg Psychiatry* 2013;84:893-900.

(7) Jackson CA, Hutchison A, Dennis MS, Wardlaw JM, Lindgren A, Norrving B et al. Differing risk factor profiles of ischemic stroke subtypes: evidence for a distinct lacunar arteriopathy? *Stroke* 2010;41:624-9.

(8) Morris Z, Whiteley WN, Longstreth WT, Jr., Weber F, Lee YC, Tsushima Y et al. Incidental findings on brain magnetic resonance imaging: systematic review and meta-analysis. *BMJ* 2009;339:b3016.

(9) Feigin VL, Forouzanfar MH, Krishnamurthi R, Mensah GA, Connor M, Bennett DA et al. Global and regional burden of stroke during 1990-2010: findings from the Global Burden of Disease Study 2010. *Lancet* 2014;383:245-54.

(10) Wardlaw JM, Smith EE, Biessels GJ, Cordonnier C, Fazekas F, Frayne R et al. Neuroimaging standards for research into small vessel disease and its contribution to ageing and neurodegeneration: a united approach. *Lancet Neurol* 2013;12:822-38.

(11) The IST-3 Collaborative Group. Association between brain imaging signs, early and late outcomes, and response to intravenous alteplase after acute ischaemic stroke in the third International Stroke Trial (IST-3): secondary analysis of a randomised controlled trial. *Lancet Neurol* 2015;14:485-96.

(12) Debette S, Markus HS. The clinical importance of white matter hyperintensities on brain magnetic resonance imaging: systematic review and meta-analysis. *BMJ* 2010;341:c3666.

(13) Vermeer SE, Longstreth WT, Jr., Koudstaal PJ. Silent brain infarcts: a systematic review. *Lancet Neurol* 2007;6:611-9.

(14) Staals J, Makin SDJ, Doubal F, Dennis M, Wardlaw JM. Stroke subtype, vascular risk factors and total MRI brain small vessel disease burden. *Neurology* 2014;83:1228-34.

(15) Jickling GC, Chen C. Rating total cerebral small-vessel disease: Does it add up? *Neurology* 2014;doi: 10.1212/WNL.0000000000000843.

(16) Bath PM, Wardlaw JM. Pharmacological treatment and prevention of cerebral small vessel disease: a review of potential interventions. *Int J Stroke* 2015;10:469-78.

(17) Del Bene A, Makin SDJ, Doubal FN, Inzitari D, Wardlaw JM. Variation in risk factors for recent small subcortical infarcts with infarct size, shape and location. *Stroke* 2013;44:3000-6.

(18) Wardlaw JM, Allerhand M, Doubal FN, Valdes Hernandez M, Morris Z, Gow AJ et al. Vascular risk factors, large artery atheroma and brain white matter hyperintensities. *Neurology* 2014;82:1331-8.

(19) Bailey EL, Smith C, Sudlow CLM, Wardlaw JM. Pathology of lacunar ischaemic stroke in humans - a systematic review. *Brain Pathol* 2012;22:583-91.

(20) Hassan A, Hunt BJ, O'Sullivan M, Parmar K, Bamford JM, Briley D et al. Markers of endothelial dysfunction in lacunar infarction and ischaemic leukoaraiosis. *Brain* 2003;126:424-32.

(21) Stevenson SF, Doubal FN, Shuler K, Wardlaw JM. A systematic review of dynamic cerebral and peripheral endothelial function in lacunar stroke versus controls. *Stroke* 2010;41:e434-e442.

(22) Wardlaw JM, Doubal F, Armitage P, Chappell F, Carpenter T, Maniega SM et al. Lacunar stroke is associated with diffuse blood-brain barrier dysfunction. *Ann Neurol* 2009;65:194-202.

(23) Farrall AJ, Wardlaw JM. Blood brain barrier: ageing and microvascular disease - systemic review and meta-analysis. *Neurobiol Aging* 2009;30:337-52.

(24) Simpson JE, Wharton SB, Cooper J, Gelsthorpe C, Baxter L, Forster G et al. Alterations of the blood-brain barrier in cerebral white matter lesions in the ageing brain. *Neurosci Lett* 2010;486:246-51.

(25) Topakian R, Barrick TR, Howe FA, Markus HS. Blood-brain barrier permeability is increased in normal-appearing white matter in patients with lacunar stroke and leucoaraiosis. *J Neurol Neurosurg Psychiatry* 2010;81:192-7.

(26) Taheri S, Gasparovic C, Huisa BN, Adair JC, Edmonds E, Prestopnik J et al. Blood-brain barrier permeability abnormalities in vascular cognitive impairment. *Stroke* 2011;42:2158-63.

(27) Wardlaw JM, Doubal FN, Eadie E, Chappell F, Shuler K, Cvoro V. Little association between intracranial arterial stenosis and lacunar stroke. *Cerebrovasc Dis* 2011;31:12-8.

(28) Deplanque D, Lavallee PC, Labreuche J, Gongora-Rivera F, Jaramillo A, Brenner D et al. Cerebral and extracerebral vasoreactivity in symptomatic lacunar stroke patients: a case-control study. *Int J Stroke* 2013;8:413-21.

(29) Aribisala BS, Morris Z, Eadie E, Thomas A, Gow A, Valdes Hernandez MC et al. Blood pressure, internal carotid artery flow parameters and age-related white matter hyperintensities. *Hypertension* 2014;63:1011-8.

(30) Fernando MS, Simpson JE, Matthews F, Brayne C, Lewis CE, Barber R et al. White matter lesions in an unselected cohort of the elderly: molecular pathology suggests origin from chronic hypoperfusion injury. *Stroke* 2006;37:1391-8.

(31) Cantin S, Villien M, Moreaud O, Tropres I, Keignart S, Chipon E et al. Impaired cerebral vasoreactivity to CO2 in Alzheimer's disease using BOLD fMRI. *Neuroimage* 2011;58:579-87.

(32) Dumas A, Dierksen GA, Gurol ME, Halpin A, Martinez-Ramirez S, Schwab K et al. Functional magnetic resonance imaging detection of vascular reactivity in cerebral amyloid angiopathy. *Ann Neurol* 2012;72:76-81.

(33) The SPS3 Investigators. Effects of clopidogrel added to aspirin in patients with recent lacunar stroke. *N Engl J Med* 2012;367:817-25.

(34) Palacio S, Hart RG, Pearce LA, Benavente OR. Effect of addition of clopidogrel to aspirin on mortality: systematic review of randomized trials. *Stroke* 2012;43:2157-62.

(35) SPS3 Study Group, Benavente OR, Coffey CS, Conwit R, Hart RG, McClure LA et al. Blood-pressure targets in patients with recent lacunar stroke: the SPS3 randomised trial. *Lancet* 2013;382:507-15.

(36) Pearce LA, McClure LA, Anderson DC, Jacova C, Sharma M, Hart RG et al. Effects of long-term blood pressure lowering and dual antiplatelet treatment on cognitive function in patients with recent lacunar stroke: a secondary analysis from the SPS3 randomised trial. *Lancet Neurol* 2014;13:1177-85.

(37) ten Dam VH, van den Heuvel DM, van Buchem MA, Westendorp RG, Bollen EL, Ford I et al. Effect of pravastatin on cerebral infarcts and white matter lesions. *Neurology* 2005;64:1807-9.

(38) Ariesen MJ, Claus SP, Rinkel GJE, Algra A. Risk factors for intracerebral hemorrhage in the general population. A systematic review. *Stroke* 2003;34:2060-6.

(39) Arboix A, Blanco-Rojas L, Marti-Vilalta JL. Advancements in understanding the mechanisms of symptomatic lacunar ischemic stroke: translation of knowledge to prevention strategies. *Expert Rev Neurother* 2014;14:261-76.

(40) Willmot M, Gray L, Gibson C, Murphy S, Bath PM. A systematic review of nitric oxide donors and L-arginine in experimental stroke; effects on infarct size and cerebral blood flow. *Nitric Oxide* 2005;12:141-9.

(41) Pedder H, Vesterinen H, Macleod M, Wardlaw J. A systematic review and meta-analysis of interventions tested in animal models of lacunar stroke. *Stroke* 2014;45:563-70.

(42) Comerota AJ. Effect on platelet function of cilostazol, clopidogrel, and aspirin, each alone or in combination. *Atherosclerosis Supplements* 2006;6:13-9.

(43) Miyamoto N, Pham LD, Hayakawa K, Matsuzaki T, Seo JH, Magnain C et al. Age-related decline in oligodendrogenesis retards white matter repair in mice. *Stroke* 2013;44:2573-8.

(44) Gotoh F, Tohgi H, Hirai S, Terashi A, Fukuuchi Y, Otomo E et al. Cilostazol stroke prevention study: A placebo-controlled double-blind trial for secondary prevention of cerebral infarction. *J Stroke Cerebrovasc Dis* 2000;9:147-57.

(45) Uchiyama S, Demaerschalk BM, Goto S, Shinohara Y, Gotoh F, Stone WM et al. Stroke prevention by cilostazol in patients with atherothrombosis: meta-analysis of placebo-controlled randomized trials. *J Stroke Cerebrovasc Dis* 2009;18:482-90.

(46) Shinohara Y, Katayama Y, Uchiyama S, Yamaguchi T, Handa S, Matsuoka K et al. Cilostazol for prevention of secondary stroke (CSPS 2): an aspirin-controlled, double-blind, randomised non-inferiority trial. *Lancet Neurol* 2010;9:959-68.

(47) DiNicolantonio JJ, Lavie CJ, Fares H, Menezes AR, O'Keefe JH, Bangalore S et al. Meta-analysis of cilostazol versus aspirin for the secondary prevention of stroke. *Am J Cardiol* 2013;112:1230-4.

(48) Bath PM, Pathansali R, Iddenden R, Bath FJ. The effect of transdermal glyceryl trinitrate, a nitric oxide donor, on blood pressure and platelet function in acute stroke. *Cerebrovasc Dis* 2001;11:265-72.

(49) Presley TD, Morgan AR, Bechtold E, Clodfelter W, Dove RW, Jennings JM et al. Acute effect of a high nitrate diet on brain perfusion in older adults. *Nitric Oxide* 2011;24:34-42.

(50) The ENOS Trial Investigators. Efficacy of nitric oxide, with or without continuing antihypertensive treatment, for management of high blood pressure in acute stroke (ENOS): a partial-factorial randomised controlled trial. *Lancet* 2015;385:617-28.

(51) Willmot M, Ghadami A, Whysall B, Clarke W, Wardlaw J, Bath PM. Transdermal glyceryl trinitrate lowers blood pressure and maintains cerebral blood flow in recent stroke. *Hypertension* 2006;47:1209-15.

(52) Sahathevan R, Brodtmann A, Donnan GA. Dementia, stroke, and vascular risk factors; a review. *Int J Stroke* 2012;7:61-73.

(53) Omote Y, Deguchi K, Tian F, Kawai H, Kurata T, Yamashita T et al. Clinical and pathological improvement in stroke-prone spontaneous hypertensive rats related to the pleiotropic effect of cilostazol. *Stroke* 2012;43:1639-46.

(54) Jeng JS, Sun Y, Lee JT, Lin RT, Chen CH, Po HL et al. The efficacy and safety of cilostazol in ischemic stroke patients with peripheral arterial disease (SPAD): protocol of a randomized, double-blind, placebo-controlled multicenter trial. *Int J Stroke* 2015;10:123-7.

(55) Han SW, Lee SS, Kim SH, Lee JH, Kim GS, Kim OJ et al. Effect of cilostazol in acute lacunar infarction based on pulsatility index of transcranial Doppler (ECLIPse): a multicenter, randomized, double-blind, placebo-controlled trial. *Eur Neurol* 2012;69:33-40.

(56) Bailey EL, Smith C, Sudlow CL, Wardlaw JM. Is the spontaneously hypertensive stroke prone rat a pertinent model of subcortical ischaemic stroke? A systematic review. *Int J Stroke* 2011;6:434-44.

(57) Rashid PA, Whitehurst A, Lawson N, Bath PM. Plasma nitric oxide (nitrate/nitrite) levels in acute stroke and their relationship with severity and outcome. *J Stroke Cerebrovasc Dis* 2003;12:82-7.

(58) Ovbiagele B, Diener HC, Yusuf S, Martin RH, Cotton D, Vinisko R et al. Level of systolic blood pressure within the normal range and risk of recurrent stroke. *JAMA* 2011;306:2137-44.

(59) Sabayan B, van Vliet P, de Ruijter W, Gussekloo J, de Craen AJ, Westendorp RG. High blood pressure, physical and cognitive function, and risk of stroke in the oldest old: the Leiden 85-Plus Study. *Stroke* 2013;44:15-20.

(60) Greenberg SM, Vernooij MW, Cordonnier C, Viswanathan A, Al-Shahi Salman R, Warach S et al. Cerebral microbleeds: a guide to detection and interpretation. *Lancet Neurol* 2009;8:165-74.

(61) Davidai G, Cotton D, Gorelick P, Bath PM, Lipton RB, Sacco R et al. Dipyridamole-induced headache and lower recurrence risk in secondary prevention of ischaemic stroke: a post hoc analysis. *Eur J Neurol* 2014;21:1311-7.

(62) Castanho TC, Amorim L, Zihl J, Palha JA, Sousa N, Santos NC. Telephone-based screening tools for mild cognitive impairment and dementia in aging studies: a review of validated instruments. *Front Aging Neurosci* 2014;6:16.

(63) The IST-3 Collaborative Group. The benefits and harms of intravenous thrombolysis with recombinant tissue plasminogen activator within 6 h of acute ischaemic stroke (the third international stroke trial [IST-3]): a randomised controlled trial. *Lancet* 2012;379:2352-63.

(64) Schmidt R, Berghold A, Jokinen H, Gouw AA, van der Flier WM, Barkhof F et al. White matter lesion progression in LADIS: frequency, clinical effects, and sample size calculations. *Stroke* 2012;43:2643-7.

(65) Wardlaw JM, Doubal FN, Valdes-Hernandez MC, Wang X, Chappell FM, Shuler K et al. Blood-brain barrier permeability and long term clinical and imaging outcomes in cerebral small vessel disease. *Stroke* 2013;44:525-7.

(66) Pavlovic AM, Pekmezovic T, Tomic G, Trajkovic JZ, Sternic N. Baseline predictors of cognitive decline in patients with cerebral small vessel disease. *J Alzheimers Dis* 2014;42:S37-S43.

(67) The Optimising the Analysis of Stroke Trials (OAST) Collaboration, Gray LJ, Bath PM, Collier T. Should stroke trials adjust functional outcome for baseline prognostic factors? *Stroke* 2009;40:888-94.

(68) The Optimising Analysis of Stroke Trials (OAST) Collaboration. Calculation of sample size for stroke trials assessing functional outcome: comparison of binary and ordinal approaches. *Int J Stroke* 2008;3:78-84.

SUPPLEMENT – LACunar Intervention Trial 2 (LACI-2) Statistical Analysis Plan

SECTION 1. ADMINISTRATIVE INFORMATION

1 Title and trial registration
1a Title: Lacunar Intervention Trial 2
Acronym: LACI-2
1b Registration: ISRCTN14911850; IRAS project number: 206480

2 SAP version: 1.5 (06 June 2022)

3 Protocol version: 7.0 (14 October 2020)

4 SAP revisions
4a Revision history:
Version 1.4 to 1.5
- Text added about the planned soft database lock and analysis (section 13a).
- Text added explaining comparison of dual versus no treatment (section 27a).
- Differences and p values removed (Tables 1, 5).
- Analyses comparing dual versus no treatment do not include all four groups so cilostazol only and ISMN groups removed (Table 9).

4b Justification for each revision: N/A
4c Timing of SAP revisions: These antedate data lock and analysis. Where there is a difference between the protocol (on website), published protocol [1] and SAP, the SAP will take precedence.

5 Roles and responsibilities
Author: Philip M Bath
Responsible statisticians: Iris Mhlanga (blinded statistician), Lisa J Woodhouse (blinded statistician), Alan A Montgomery
Chief Investigator: Joanna M Wardlaw
Contributors and roles:
Philip M Bath, Iris Mhlanga, Lisa J Woodhouse, Fergus Doubal, Katherine Oatey, Alan A Montgomery, Joanna M Wardlaw, for the LACI-2 Investigators*

6 Signatures

Role	Name	Signature	Date
6a Author:	Philip Bath		
6b Senior statistician	Alan Montgomery		
6c Chief Investigator:	Joanna Wardlaw		

SECTION 2. INTRODUCTION

7 Background and rationale
Prior to analysis and presentation of the primary results, this publication presents the statistical analysis plan (SAP) [2,3] alongside the detailed listing of baseline

characteristics presented in the accompanying baseline paper. This Supporting Information Appendix S1 details the full SAP and is presented prior to locking of the study database so that analyses are not data driven or reported selectively.[4] In addition to the SAP, we also list planned secondary analyses and substudies. The SAP follows the recommended layout.[2 3]

8 Objectives

8a Primary Objective: To determine whether a prospective, randomised trial of cilostazol and ISMN, individually or in combination, on a background of guideline stroke prevention therapy, in lacunar ischaemic stroke is feasible in the UK, thence proceeding as seamlessly as possible to a large phase III trial.
8b Secondary Objectives: To assess drug tolerability, safety, recruitment rates and accuracy, outcome event rates and retention in preparation to a large phase III randomised controlled trial to prevent recurrent lacunar stroke and physical and cognitive impairment.

This SAP focuses on these primary and secondary objectives. Planned follow-on publications will address tertiary questions.

SECTION 3. STUDY METHODS

9 Trial design

Prospective randomised open-label blinded end-point (PROBE) partial-factorial phase IIb/c trial aiming to recruit 400 patients recruited in UK Stroke Network Centres, with follow-up to one year.

10 Randomisation: By central computer-generated allocation at the University of Nottingham with minimisation on key prognostic factors: age, sex, stroke severity (NIHSS), dependency resulting from the stroke, systolic blood pressure ≤/>140 mmHg, smoking status, time after stroke, and years of education.

11 Sample size/power considerations

Conservatively, we have used sample size calculations based on binary measures. Use of ordinal measures at the time of analysis will increase statistical power.
11a Event rates: Annual event rates (Table A) were assessed from trials (SPS3,[5] lacunar patients in ENOS,[6 7] IST-3 [8 9]) and observational data (LADIS;[10] our [11-13] and other [14] studies). All-cause death rates were assumed to be 2.0% with upper 95% CI of 4% in 400 patients.[5] Hence, the sample size was set at 400 participants.

Table A Annual absolute risks (%) of outcome events after lacunar stroke

Vascular death	Non-vascular death	Non-fatal IS or TIA	Non-fatal ICH	MI	MACE	Dependent (mRS 3-5)	Cognitive impairment	Dementia
1.8	0.5	2.5	0.5	0.6	3	15	30	15

ICH: intracerebral haemorrhage; IS: ischaemic stroke; MACE: major adverse cardiac events; MI: myocardial infarction; mRS: modified Rankin scale; TIA: transient ischaemic stroke

11b Comparison of two groups in a future phase III trial: Assuming power 0.80, alpha=0.05, 1:1 randomisation, composite event rate (MACE, dementia, non-vascular death, new MRI signs) 45% and absolute reduction 9% (relative risk reduction 20%), and loss to follow-up 10%, a sample size of 1100 will be needed. A number of outcomes are relevant to patients with SVD and using these has implications for the sample size (Table B).

Table B. Sample size for composite outcome in main trial using estimated event rates.[1]

Composite model	A	B	Ci	CiI	D
Composite outcome for phase III	MACE, dementia, non-vascular death, new MR signs	MACE, dementia, death	MACE, cognitive decline, dependency decline, all-cause death		MACE, cognitive impairment, dependency, all-cause death
1-beta (power)	80%	80%	80%	80%	80%
Event rate, control, pa	50%	10%	30%	30%	45%
Relative risk reduction	20%	20%	20%	30%	20%
Event rate, active, pa	40%	8%	24%	21%	36%
Total sample size	950	6626	1784	778	976
Total trial size, including losses	1250	7400	2000	900	1100

MACE: major adverse cardiac events; MRI: magnetic resonance imaging

12 Framework
The primary objectives are to assess the feasibility of recruitment and adherence to medication.

13 Statistical interim analyses and stopping guidance
13a Interim analyses: Data are tabulated twice annually prior to Trial Steering Committee (TSC) and Data Monitoring Committee (DMC) meetings. No unblinded comparative analyses will be performed until data collection has been completed and the database locked.

Prior to the final database lock, the database will be subject to a soft lock and the provisional data tabulated and analysed for review by the TSC and DMC. Any final queries will be raised and resolved prior to final database lock. Members of staff still involved in the collation of data and resolution of data queries will not attend the meeting and the data reviewed at the meeting will remain strictly confidential until the point of final database lock to avoid any bias.

13b Adjustments of significance level: There is no planned adjustment.
13c Stopping rules: There are no formal stopping rules, but the DMC have responsibility to make recommendations to pause or modify the study, should there be any safety or efficacy considerations.

14 Timing of final analyses
These will be performed once data collection has been completed and the database has been locked.

15 Timing of outcome assessments
Assessments will be performed at baseline, 1-2 weeks, 3-4 weeks, 6 and 12 months (***Table C***).

Table C. Assessments at baseline and follow-up by time point (adapted from protocol and [1]).

**Assessment**	**Prior to Baseline**	**Visit 1 Baseline**	**Week 1-2**	**Week 3-4**	**Month 6**	**Month 12**
Screening for eligibility and consent [†]	X^{S}					
Confirm and document ongoing consent		X^{S}				
Medical including drug history		X^{S}				
Assess MR or CT diagnostic scan; send copy to Edinburgh		X^{S}				
Randomisation		X^{S}				
Haematology (full blood count) and Biochemistry (urea, electrolytes, creatinine) – most recent value obtained since time of index stroke is acceptable unless clinical reason to expect change		X^{S}				
Blood pressure		X^{S}				X^{S} [‡]
Cognitive test: document years of education; Montreal Cognitive Assessment (MOCA)		X^{S}				
Timed Trail Making Test B		X^{S}				X^{S} [‡]
Dispense trial medication[2]		X^{S}			X^{S}	
Structured questionnaire: symptoms; medication history and IMP tablet adherence			X^{S}	X^{S}	X^{C}	X^{C}
Structured questionnaire: recurrent vascular events, mRS, TICS, t-MOCA, SIS, ZUNG					X^{C}	X^{C}
Obtain IQCODE (post/phone) from relative						X^{C}
Follow-up brain MRI						X^{S}
Health Economics data: EQ-5D-5L, EQ-VAS						X^{C}
Adverse event / con meds reporting as necessary			X^{S}	X^{S}	$X^{S,C}$	$X^{S,C}$

[†] Consent will be obtained before the data collection procedures commence or randomisation is performed. Randomisation occurs at the end of the baseline visit.

[‡] at 12 months in some centres only.

[2] Dispensing in 3-monthly intervals is allowed.

[S] Assessment performed by local site team.

[C] Assessment performed by blinded assessor who is part of the central trial team.

SIS: Stroke Impact Scale; TICS: telephone interview for cognitive status; t-MOCA: telephone MOCA.

SECTION 4. STATISTICAL PRINCIPLES

Confidence and p values
16 Level of statistical significance
The results of analyses and comparisons will be shown with p<0.05.

17 Multiplicity
No adjustment will be made for multiplicity.

18 Levels of confidence intervals
The results of analyses and comparisons will be shown with 95% confidence intervals.

19 Adherence
19a Definition: 75% of patients will be able to tolerate trial medication, in at least half dose, up to one year after randomisation (i.e. less than 25% will stop trial medication completely through inability to tolerate the drugs).
19b Adherence presentation: See Table 4.
19c Protocol deviations: Protocol violations will be reported to the sponsor within 24 hours of becoming aware of the violation. Protocol deviations will be recorded in a protocol deviation log with these submitted to the sponsors every 3 months.
19d Protocol deviation presentation: Listing of violations and deviations and their frequency.

20 Analysis populations
Three populations are defined:
1. Intention-to-treat: All consented participants with a primary outcome measure.
2. Per protocol: All consented participants with a primary outcome measure who received at least one dose of randomised medication and who had no protocol violation, e.g. they fulfilled all eligibility criteria.
3. Safety: All consented participants who received at least one dose of randomised medication.

Multiple variable analyses will include all patients with complete data for the dependent and each independent variable. All available data will be used, and missing data will not be imputed.

SECTION 5. TRIAL POPULATION

21 Screening data
No screening logs will be kept so that data collection can be prioritised.

22 Eligibility
1. Clinical lacunar stroke syndrome.
2. Brain scanning with MR including diffusion imaging wherever possible, and obtained soon after the presentation with stroke, which showed either:
    a. A recent, relevant (in time and location) acute small subcortical (i.e. lacunar) infarct on diffusion MR imaging.
    b. If no visible acute small subcortical infarct on diffusion MR imaging then there is no competing pathology as a cause for stroke (e.g. no acute cortical infarct, no acute intra-cerebral haemorrhage, no stroke mimic such as tumour, subdural haematoma);
    c. If only a CT brain scan is available, then there is a small relevant (in time and location) subcortical (i.e. acute lacunar) infarct, or if no infarct then there is no competing pathology as a cause for stroke (e.g. no acute cortical infarct, no acute intra-cerebral haemorrhage, no stroke mimic such as tumour, subdural haematoma).

3. Age >30 years.
4. Independent in activities of daily living (modified Rankin Scale <=2).
5. Capacity to give consent themselves.

23 Recruitment
Recruitment will be summarised in a CONSORT flow diagram.

24 Withdrawal/follow-up
Withdrawals, and missed follow-ups and their timing will be summarised in the CONSORT flow diagram.

25 Baseline patient characteristics
25a Baseline characteristics: These will comprise demographic, education, premorbid function, cognitive ability, medical history, blood pressure, stroke investigations, clinical and brain imaging parameters (Table 1).
25b Summarisation: Data will be shown as number (%), median [interquartile range] or mean (standard deviation) as appropriate.

SECTION 6. ANALYSIS

26 Outcome definitions
26a Specifications:
Primary endpoint
Feasibility of a Phase III efficacy trial assessed as:
- Recruitment of sufficient patients, i.e. 400 patients in 24 months in the UK (and taking account of interruption due to COVID-19).
- >95% of randomised patients are retained for follow-up at one year.

Secondary outcomes - participant
Tolerability: 75% of patients will be able to tolerate trial medication, in at least half dose, up to one year after randomisation (i.e. less than 25% will stop trial medication completely through inability to tolerate the drugs).
Safety
- Symptoms of systemic or intracranial bleeding.
- The absolute risk of death, including fatal haemorrhage, does not differ significantly, i.e. fall outside the upper 95% CI of 2% per year on trial drugs versus no trial drugs, when given in addition to guideline stroke prevention drugs.
- There are no new ischaemic or haemorrhagic brain lesions or increase in SVD lesions on one year MRI significantly (at the p<0.01 level).

Efficacy
- Individual event-rates for stroke, TIA, myocardial ischaemia, cognitive impairment and dementia.
- The *combined rate* of recurrent stroke, MI, death, mild cognitive impairment (including dementia), dependency and new stroke lesions on scanning at 1 year will be 40-50% at one year after enrolment in order to allow detection of a modest but clinically-important reduction in poor outcomes in a phase III trial.
- Health economic measures include the health utility score (EQ-5D-5L) and the visual analogue score (EQVAS) at 12 months.

26b Units: Units will be shown in tables.
26c Calculations/transformations: Quality of life using UK weightings.
Brain frailty
Based on neuroimaging:
- Brain frailty = Atrophy + WML + Previous stroke lesion [7]

- SVD score for CT = WML, lacunes; for MRI includes WMH, lacunes, PVS and microbleeds [15]

Montreal cognitive assessment-modified (MoCA-m) trails

Since Trails A and B are performed, the MoCA trail is not collected but rather estimated from the Trails B score:

- If Trails B score <12 then MoCA trail = 0
- If Trails B score >=12 then MoCA trail = 1

27 Analysis methods

27a Methods

Primary endpoint

- Tabulation and graphical presentation of participant recruitment aiming for 400 participants in 24 months.
- Tabulation of retention of participants at one year aiming for >95%.

Analyses of secondary outcomes

Tabulations of:

- Tolerability to trial medications aiming for 75% of patients taking at least half dose for up to one year after randomisation.
- Death, including fatal haemorrhage, aiming for less than outside the upper 95% CI of 2% per year.
- The *combined rate* of recurrent stroke, MI, death, cognitive impairment and dependency, aiming for 40-50% at one year after enrolment.

Comparison of rates of events between the treatment groups: cilostazol vs no cilostazol, ISMN vs no ISMN, and cilostazol and ISMN vs neither, for:

- Systemic or intracranial bleeding, recurrent cerebral and systemic vascular events, and vascular and non-vascular causes of death.
- Death, all cause.
- New ischaemic or haemorrhagic stroke lesion or increase in SVD lesions on MRI.
- Composite of: recurrent clinically-evident stroke, MI, death, cognitive impairment (including dementia) and dependency.
- Individual event: stroke (clinically-evident or imaging-detected ischaemic or haemorrhagic stroke to be reported separately), TIA, myocardial ischaemia, cognitive impairment and dementia.
- New infarcts and haemorrhages, absolute and change in WMH, microhaemorrhages, lacunes, atrophy imaging variables from central read of baseline imaging and one year MRI
- The comparisons of combined cilostazol and ISMN versus neither, whilst being very underpowered statistically, are presented since these may be the two groups studied in the planned follow-on trial.

Central tendency, comparisons and regressions will be analysed as follows (***Table D***).

Table D. Descriptive and analytical statistics

	Binary	Nominal	Ordinal	Continuous
Central tendency and distribution	N (%)	N (%)	Median [interquartile range]	Mean (standard deviation)
Comparisons	Chi-square (2x2)	Chi-square (2x2, or rxc)	Mann-Whitney U or Kruskal-Wallis	t-test (pooled) or 1-way ANOVA
Regression	Binary logistic regression (BLR)	-	Ordinal logistic regression (OLR)	Multiple linear regression (MLR)

27b Covariate adjustment: Analyses will be adjusted for minimisation covariates:

- Age, sex, stroke severity (NIHSS), dependency resulting from the stroke, systolic blood pressure, smoking status, time after stroke, years of education.

Covariate adjustment with continuous variables (age, NIHSS, SBP, time after stroke, years of education) will use original, not dichotomised, data.

27c Assumption checking: The assumption of proportionality will be tested using the likelihood test.

27d Alternative methods: If the data fail the assumption of proportionality (tested using the likelihood test), we will use alternative methods such as multiple logistic regression.

27e Sensitivity analyses: In addition to assessment of raw data, the primary outcome will be analysed using additional statistical approaches in sensitivity analyses:

- Unadjusted analysis
- Imputation (multiple regression imputation) of missing data (adjusted) [16]

27f Subgroup analyses: The secondary endpoint of the composite of: recurrent stroke, MI, death, cognitive impairment and dependency, will be studied in:

- Pre-specified subgroups comprising the minimisation variables.
- Any other variables demonstrating imbalance at baseline.

The results of these subgroup analyses will not be adjusted for multiple testing. These analyses are planned for the phase III efficacy trial and so will be tested in the present study.

28 Missing data

Missing data may occur at outcome level or at test level or within a test at component item level. Notably, some tests have to exclude components if performed by telephone and/or postal questionnaire or require a one year MRI. There is often a relationship between inability to complete outcome assessment and cognitive function or neurological deficit after stroke (e.g. inability to hold a pen) and so assumptions around random missingness may not be valid, even if the patterns of missing data initially suggest 'missing completely at random' status. Indeed, failure to complete a test may be an indicator of cognitive impairment rather than real missingness.

The approaches taken to missing cognitive and other data can have a substantial effect on epidemiological estimates.[16] We will use the approach that makes greatest use of available data.

Where in study data are not available, or participants are lost to follow-up, we have permissions to allow for linkage of the study dataset to primary and secondary care electronic health records. This will allow for an assessment of clinical outcomes across all the participants.

29 Additional analyses

Global outcomes

We will assess global outcomes integrating multiple scores into one analysis and so provide a more holistic measure and improve statistical power. We will assess global outcomes comprising:

- Recurrent ordinal stroke (clinically-evident and/or imaging-detected),[17] ordinal MI, cognition (MoCA), dependency (mRS), quality of life (EQ-5D).

Analyses will use the Wei-Lachin test [18-21] with comparison of data at 1 year.

Cognitive domains, based on DSM-V

We will categorise cognition into 7- and 4-level ordinal scales based on DSM-V [22] categorisation (Table E).[23] We will calculate scores for cognitive domains using sub-scores of MoCA (or TICS if missing) although we recognise that these global cognitive assessments have some test items that map to a more than one domain, e.g. the

clock drawing test in the MoCA includes aspects of attention, executive function and visual-perceptual function.

- Learning and memory: orientation in place (from MoCA), delayed recall of five word (MoCA), and recall and delayed recall of ten words (TICS)
- Language: using comprehension, semantic and recent memory (from MoCA; similar elements in TICS-M)
- Perceptual-motor function: Cube copy and clock drawing from MoCA
- Executive function: Trail making tests A & B, verbal fluency test (VFT-phonemic)-F (from MoCA); verbal fluency test (VFT-semantic)-animals; clock drawing test (from MoCA); digits forward (from MoCA); digits backward (from MoCA)
- Complex attention: using serial sevens subtraction (MoCA), letter tapping (MoCA)
- Social cognition is not classically assessed in cognitive screening tools and there are no agreed generic short form assessments for social cognition. Aspects of social cognition will be assessed through informant data and NPI-Q although these are not part of the core outcome set.

Table E. Categorisation of cognition based on DSM V with operationalisation (adapted summary from [23]).

	**Seven-level categorisation and operationalisation**	**Four-level categorisation and operationalisation**
**Normal cognition**	No evidence of cognitive impairment<br>(T-MoCA:20-22 AND TICS-m: 25-39)	No evidence of cognitive impairment<br>(T-MoCA:20-22 AND TICS-m: 25-39)
**Minor Neurocognitive disorder (mild cognitive impairment)**	**Single domain**<br>Scores are reduced by > 1 point in only one cognitive domain of T-MoCA	Evidence of cognitive impairment<br>(T-MoCA: 15-19 OR TICS-m: 17-24)<br>AND<br>No evidence of functional impairment<br>(mRS <2 OR no change in mRS if pre-stroke mRS >1)
	**Multi-domain**<br>Scores are reduced by > 1 point in more than one cognitive domain of T-MoCA	
**Major neurocognitive disorder**	**Mild cognitive impairments**<br>(T-MoCA 15-19 OR TICS-m 17-23)<br>**AND**<br>**Mild dysfunction**<br>(mRS <3)	Persisting cognitive impairment<br>(T-MoCA score <19 OR TICS-m<24 on more than one follow-up)<br>AND<br>Functional impairment<br>(mRS ≥2 or IQCODE >3.6 at final follow-up)<br><br>OR<br><br>Any clinical diagnosis of dementia made independent of study, e.g. by memory clinic, in primary care,<br>recording of dementia on death certification, prescription of cholinesterase inhibitor or memantine
	**Moderate cognitive impairments**<br>(T-MoCA 10-14 OR TICS-m 12-16)<br>**AND**<br>**Moderate dysfunction**<br>(mRS 3 or 4)	
	**Severe cognitive impairments**<br>(T-MoCA <10 OR TICS-m <12)<br>**AND**<br>**Severe dysfunction**	

	In a care-home OR mRS 4,5	
**Death**	Death (mRS 6)	Death (mRS 6)

30 Harms
These are presented as serious adverse events in Tables 6a-c and 7a-c.

31 Statistical software
Statistical Analysis System (SAS) version 9.4, SAS Institute Incorporation, Cary, North Carolina.

SECTION 7. ADDITIONAL INFORMATION

Confounding covariates
The primary outcome is recruitment, and this will be tabulated; as such, there are no confounding covariates.

The possible primary outcome in any following trial will likely be a composite comprising MACE, dementia or mild cognitive impairment, non-vascular death and new MRI signs and these event rates will be assessed in the current trial. The components are likely to be correlated. Example confounding variables are given and these are categorised by whether these were 'measured', as per routine practice, or 'unmeasured'; the latter will lead to residual confounding.

Composite outcome
Measured variables: Age, highest educational attainment, main occupation, socioeconomic status, stroke severity, function at randomisation, cognitive ability at randomisation, diabetes mellitus, hypertension, smoking, carotid disease, blood pressure, prescribed medications, time from stroke to randomisation, presence of a relevant infarct and SVD lesion severity on brain imaging.
Unmeasured: Examples are social isolation, vision, hearing, cardiac function (atrial fibrillation and heart failure are documented) and post-stroke complications.

Governance
LACI-2 is funded by the British Heart Foundation (CS/15/5/31475) and approved by the East Midlands – Nottingham 2 Research Ethics Committee (Ref: 17/EM/0077). The Sponsor is the ACCORD office, University of Edinburgh and NHS Lothian. NHS Research and Development/ Innovation approval is given at each participating site. The study is adopted by the National Institute for Health Research (NIHR) Clinical Research Network in England and the Stroke Research Network in Scotland.

Minimising bias
Multiple approaches are taken to minimise bias: central data registration with real-time on-line validation; minimisation at randomisation; blinded central postal and/or telephone assessment of outcomes; blinded adjudication of neuroimaging; inclusion of patients enrolled in other studies (co-enrolment) where feasible; analysis by intention-to-enrol (i.e. all participants) and in pre-specified subgroups.

Publications, published and planned
1. Protocol - published
2. SAP and baseline data - this publication
3. Primary results paper
4. Other secondary publications as determined by the Trial Steering Committee

Data sharing
In the future, the anonymised study data will be made available for use by external investigators in appropriate analyses upon request via a publicly accessible portal (e.g. University of Edinburgh data share). Data from LACI-2 will also be shared as appropriate with individual patient data pooling projects involving stroke and dementia; a non-inclusive list includes:

- The Cerebrovascular diseases database, Edinburgh (https://www.ed.ac.uk/clinical-sciences/edinburgh-imaging/research/themes-and-topics/analysis-and-processing/image-databanks/cerebrovascular-diseases-image-databank)
- Dementia Platform UK data portal (https://www.dementiasplatform.uk/)
- Virtual International Stroke Trials Archive-Cognition (VISTA-COG)
- Virtual International Cardiovascular and Cognitive Trials Archive (VICCTA, http://www.virtualtrialsarchives.org)
- META-VCI Map (https://metavcimap.org/)
- STROKOG (https://cheba.unsw.edu.au/consortia/strokog)

Similarly, anonymised neuroimaging data will be published.[24] The mechanisms and processes for managing external access will be determined during the course of the study. Proposals will be considered by the LACI-2 Trial Steering Committee.

ABREVIATIONS

Abbreviation	Full Text
CI	Confidence Interval
cSVD	Cerebral small vessel disease
CT	Computed Tomography
DMC	Data Monitoring Committee
ECG	Electrocardiogram
EQVAS	EuroQol-Visual Analogue Scale
EQ-5D-5L	EuroQoL- 5 Dimension- 5 Level quality of life questionnaire
F/T	Full Time
ICH	Intracerebral Haemorrhage
IMP	Investigational Medicinal Product
IS	Ischaemic Stroke
ISMN	Isosorbide Mononitrate
LACI-2	Lacunar Intervention Trial-2
LACS	lacunar syndrome
LVH	left ventricular hypertrophy
MACE	Major Adverse Cardiac Events
MI	Myocardial Infarction
MOCA	Montreal Cognitive Assessment
MRI	Magnetic Resonance Imaging
mRS	Modified Rankin scale
NIHSS	National Institutes Health Stroke Scale
NO	Nitric oxide
PACS	Partial Anterior Circulation Syndrome;
PROBE	Prospective Randomised Open-label Blinded-Endpoint
PGI2	Prostacyclin
POCS	Posterior Circulation Syndrome
P/T	Part Time
SAP	Statistical Analysis Plan
SAS	Statistical Analysis System
SBP	Systolic Blood Pressure
SIS	Stroke Impact Scale
SVD	Small vessel disease
TACS	Total Anterior Circulation Syndrome
TIA	Transient Ischaemic Attack
TICS	Telephone Interview for Cognitive Status
t-MOCA	Telephone MOCA
TSC	Trial Steering Committee
WMH	White Matter Hyperintensity

REFERENCES

1. Wardlaw J, Bath PMW, Doubal F, et al. Protocol: The Lacunar Intervention Trial 2 (LACI-2). A trial of two repurposed licenced drugs to prevent progression of cerebral small vessel disease. *Eur Stroke J* 2020;5(3):297-308. doi: 10.1177/2396987320920110 [published Online First: 20200420]
2. Gamble C, Krishan A, Stocken D, et al. Guidelines for the Content of Statistical Analysis Plans in Clinical Trials. *JAMA* 2017;318(23):2337-43. doi: 10.1001/jama.2017.18556
3. Hemming K, Kearney A, Gamble C, et al. Prospective reporting of statistical analysis plans for randomised controlled trials. *Trials* 2020;21(1):898. doi: 10.1186/s13063-020-04828-8 [published Online First: 20201028]
4. MacMahon S, Collins R. Reliable assessment of the effects of treatment on mortality and major morbidity, II: observational studies. *Lancet* 2001;357(9254):455-62. doi: 10.1016/s0140-6736(00)04017-4 [published Online First: 2001/03/29]
5. SPS3 Investigators BO, Hart RG, McClure LA, Szychowski JM, Coffey CS, Pearce LA. Effects of clopidogrel added to asprin in patients with recent lacunar stroke. *New England Journal of Medicine* 2012;376(9):817-25. doi: 10.1056/NEJMoa1204133
6. Bath PM, Woodhouse L, Scutt P, et al. Efficacy of nitric oxide, with or without continuing antihypertensive treatment, for management of high blood pressure in acute stroke (ENOS): a partial-factorial randomised controlled trial. *Lancet* 2015;385(9968):617-28. doi: 10.1016/s0140-6736(14)61121-1 [published Online First: 2014/12/04]
7. Appleton JP, Woodhouse LJ, Adami A, et al. Imaging markers of small vessel disease and brain frailty, and outcomes in acute stroke. *Neurology* 2020;94(5):e439-e52. doi: 10.1212/WNL.0000000000008881 [published Online First: 2019/12/27]
8. Sandercock P, Wardlaw JM, Lindley RI, et al. The benefits and harms of intravenous thrombolysis with recombinant tissue plasminogen activator within 6 h of acute ischaemic stroke (the third international stroke trial [IST-3]): a randomised controlled trial. *Lancet* 2012;379(9834):2352-63. doi: 10.1016/S0140-6736(12)60768-5 [published Online First: 20120523]
9. Sandercock P, Wardlaw JM, Dennis M, et al. Effect of thrombolysis with alteplase within 6 h of acute ischaemic stroke on long-term outcomes (the third International Stroke Trial IST-3): 18-month follow-up of a randomised controlled trial. *Lancet Neurology* 2013;12(8):768-76. doi: 10.1016/s1474-4422(13)70130-3
10. Schmidt R, Berghold A, Jokinen H, et al. White matter lesion progression in LADIS: frequency, clinical effects, and sample size calculations. *Stroke* 2012;43(10):2643-7. doi: 10.1161/strokeaha.112.662593 [published Online First: 2012/08/11]
11. McHutchison CA, Cvoro V, Makin S, et al. Functional, cognitive and physical outcomes 3 years after minor lacunar or cortical ischaemic stroke. *J Neurol Neurosurg Psychiatry* 2019;90(4):436-43. doi: 10.1136/jnnp-2018-319134 [published Online First: 20181215]
12. Wardlaw JM, Doubal FN, Valdes-Hernandez M, et al. Blood-brain barrier permeability and long-term clinical and imaging outcomes in cerebral small vessel disease. *Stroke* 2013;44(2):525-7. doi: 10.1161/strokeaha.112.669994 [published Online First: 2012/12/13]
13. Chappell FM, Del Carmen Valdés Hernández M, Makin SD, et al. Sample size considerations for trials using cerebral white matter hyperintensity progression as an intermediate outcome at 1 year after mild stroke: results of a prospective cohort study. *Trials* 2017;18(1):78. doi: 10.1186/s13063-017-1825-7 [published Online First: 20170221]
14. Pavlovic AM, Pekmezovic T, Tomic G, et al. Baseline predictors of cognitive decline in patients with cerebral small vessel disease. *Journal of Alzheimer's disease : JAD* 2014;42 Suppl 3:S37-43. doi: 10.3233/JAD-132606

15. Staals J, Makin SD, Doubal FN, et al. Stroke subtype, vascular risk factors, and total MRI brain small-vessel disease burden. *Neurology* 2014;83(14):1228-34. doi: 10.1212/WNL.0000000000000837 [published Online First: 20140827]
16. Lees RA, Hendry Ba K, Broomfield N, et al. Cognitive assessment in stroke: feasibility and test properties using differing approaches to scoring of incomplete items. *Int J Geriatr Psychiatry* 2017;32(10):1072-78. doi: 10.1002/gps.4568 [published Online First: 2016/08/16]
17. Bath PM, Woodhouse LJ, Appleton JP, et al. Antiplatelet therapy with aspirin, clopidogrel, and dipyridamole versus clopidogrel alone or aspirin and dipyridamole in patients with acute cerebral ischaemia (TARDIS): a randomised, open-label, phase 3 superiority trial. *Lancet* 2018;391(10123):850-59. doi: 10.1016/s0140-6736(17)32849-0 [published Online First: 2017/12/25]
18. Lachin J. Applications of the Wei-Lachin multivariate one-sided test for multiple outcomes on possibly different scales. *PLoS One* 2014;9(10) doi: 10.1371/journal.pone.0108784
19. Muresanu D, Heiss W, Hoemberg V, et al. Cerebrolysin and Recovery After Stroke (CARS): A Randomized, Placebo-Controlled, Double-Blind, Multicenter Trial. *Stroke* 2016;47(1):151-9. doi: 10.1161/STROKEAHA.115.009416 [published Online First: 12 Nov 2015]
20. Appleton J, Scutt P, Sprigg N, et al. Hypercholesterolaemia and vascular dementia. *Clinical Sciences* 2017;131(14):1561-78. doi: 10.1042/CS20160382 [published Online First: 21 March 2017]
21. Bath PM, Woodhouse LJ, Krishnan K, et al. Prehospital Transdermal Glyceryl Trinitrate for Ultra-Acute Intracerebral Hemorrhage: Data From the RIGHT-2 Trial. *Stroke* 2019;50(11):3064-71. doi: 10.1161/STROKEAHA.119.026389 [published Online First: 2019/10/07]
22. Sachdev PS, Blacker D, Blazer DG, et al. Classifying neurocognitive disorders: the DSM-5 approach. *Nature reviews Neurology* 2014;10(11):634-42. doi: 10.1038/nrneurol.2014.181 [published Online First: 2014/09/30]
23. Wardlaw JM, Doubal F, Brown R, et al. Rates, risks and routes to reduce vascular dementia (R4vad), a UK-wide multicentre prospective observational cohort study of cognition after stroke: Protocol. *European Stroke Journal* 2021;6(1):89-101. doi: 10.1177/2396987320953312
24. Wardlaw J, Bath P, Sandercock P, et al. The NeuroGrid stroke examplar clinical trial protocol. *International Journal of Stroke* 2007;2:63-9.

Main paper tables

Table 1. Baseline characteristics by treatment group isosorbide mononitrate (ISMN), cilostazol (Cil) or both (ISMN+Cil).
Data are number (%), median [interquartile range] or mean (standard deviation).

	N	All	ISMN	No ISMN	Cil	No Cil	ISMN + Cil	ISMN only	Cil only	Neither
N	XX	XX	XX	XX	XX	XX	XX	XX	XX	XX
**Demographics**										
Age (yr) †	XX	XX (X)	XX (X)	XX (X)	XX (X)	XX (X)	XX (X)	XX (X)	XX (X)	XX (X)
<=70 years	XX	XX (X)	XX (X)	XX (X)	XX	XX (X)	XX	XX (X)	XX (X)	XX (X)
Sex, female (%)	XX	XX (X)	XX (X)	XX (X)	XX (X)	XX (X)	XX (X)	XX (X)	XX (X)	XX (X)
modified Rankin Scale >1 (%) †	XX	XX (X)	XX (X)	XX (X)	XX (X)	XX (X)	XX (X)	XX (X)	XX (X)	XX (X)
Onset to randomisation (days) †	XX	XX (X)	XX (X)	XX (X)	XX (X)	XX (X)	XX (X)	XX (X)	XX (X)	XX (X)
<= 100 days	XX	XX (X)	XX (X)	XX (X)	XX (X)	XX (X)	XX (X)	XX (X)	XX (X)	XX (X)
Age completing education (yr)	XX	XX (X)	XX (X)	XX (X)	XX (X)	XX (X)	XX (X)	XX (X)	XX (X)	XX (X)
Highest education (%) †										
Primary	XX	XX (X)	XX (X)	XX (X)	XX (X)	XX (X)	XX (X)	XX (X)	XX (X)	XX (X)
Secondary	XX	XX (X)	XX (X)	XX (X)	XX (X)	XX (X)	XX (X)	XX (X)	XX (X)	XX (X)
O' level/GCSE	XX	XX (X)	XX (X)	XX (X)	XX (X)	XX (X)	XX (X)	XX (X)	XX (X)	XX (X)
A' level	XX	XX (X)	XX (X)	XX (X)	XX (X)	XX (X)	XX (X)	XX (X)	XX (X)	XX (X)
Undergraduate degree	XX	XX (X)	XX (X)	XX (X)	XX (X)	XX (X)	XX (X)	XX (X)	XX (X)	XX (X)
Postgraduate degree	XX	XX (X)	XX (X)	XX (X)	XX (X)	XX (X)	XX (X)	XX (X)	XX (X)	XX (X)
Lifestyle										
Smoking †										
Current	XX	XX (X)	XX (X)	XX (X)	XX (X)	XX (X)	XX (X)	XX (X)	XX (X)	XX (X)
Past	XX	XX (X)	XX (X)	XX (X)	XX (X)	XX (X)	XX (X)	XX (X)	XX (X)	XX (X)
Never	XX	XX (X)	XX (X)	XX (X)	XX (X)	XX (X)	XX (X)	XX (X)	XX (X)	XX (X)
**History (%)**										
Hypertension, drug treated	XX	XX (X)	XX (X)	XX (X)	XX (X)	XX (X)	XX (X)	XX (X)	XX (X)	XX (X)
Hyperlipidaemia, drug treated	XX	XX (X)	XX (X)	XX (X)	XX (X)	XX (X)	XX (X)	XX (X)	XX (X)	XX (X)
Diabetes mellitus										
Oral agents	XX	XX (X)	XX (X)	XX (X)	XX (X)	XX (X)	XX (X)	XX (X)	XX (X)	XX (X)
Insulin	XX	XX (X)	XX (X)	XX (X)	XX (X)	XX (X)	XX (X)	XX (X)	XX (X)	XX (X)
Atrial fibrillation	XX	XX (X)	XX (X)	XX (X)	XX (X)	XX (X)	XX (X)	XX (X)	XX (X)	XX (X)
Heart failure	XX	XX (X)	XX (X)	XX (X)	XX (X)	XX (X)	XX (X)	XX (X)	XX (X)	XX (X)
Previous stroke	XX	XX (X)	XX (X)	XX (X)	XX (X)	XX (X)	XX (X)	XX (X)	XX (X)	XX (X)
Previous TIA	XX	XX (X)	XX (X)	XX (X)	XX (X)	XX (X)	XX (X)	XX (X)	XX (X)	XX (X)

Family history, young stroke	XX	XX (X)	XX (X)	XX (X)	XX (X)	XX (X)	XX (X)	XX (X)	XX (X)	XX (X)
**Medications**										
Anticoagulants	XX	XX (X)	XX (X)	XX (X)	XX (X)	XX (X)	XX (X)	XX (X)	XX (X)	XX (X)
Antibiotics	XX	XX (X)	XX (X)	XX (X)	XX (X)	XX (X)	XX (X)	XX (X)	XX (X)	XX (X)
Antihypertensives	XX	XX (X)	XX (X)	XX (X)	*XX (X)*	*XX (X)*	*XX (X)*	*XX (X)*	*XX (X)*	*XX (X)*
Antiplatelets	XX	XX (X)	XX (X)	XX (X)	*XX (X)*	*XX (X)*	*XX (X)*	XX (X)	XX (X)	XX (X)
Lipid-lowering	XX	XX (X)	XX (X)	XX (X)	*XX (X)*	*XX (X)*	*XX (X)*	XX (X)	XX (X)	XX (X)
Proton pump inhibitor	XX	XX (X)	XX (X)	XX (X)	*XX (X)*	*XX (X)*	XX (X)	XX (X)	XX (X)	XX (X)
PDE5 inhibitor	XX	XX (X)	XX (X)	XX (X)	*XX (X)*	*XX (X)*	XX (X)	XX (X)	XX (X)	XX (X)
Other drugs	XX	XX (X)	XX (X)	XX (X)	*XX (X)*	*XX (X)*	XX (X)	XX (X)	XX (X)	XX (X)
No. medications /day	XX	XX (X)	XX (X)	XX (X)	XX (X)	XX (X)	XX (X)	XX (X)	XX (X)	XX (X)
Grapefruit juice (%)	XX	XX (X)	XX (X)	XX (X)	XX (X)	XX (X)	XX (X)	XX (X)	XX (X)	XX (X)
**Clinical (%)**										
Systolic BP (mmHg) †	XX	XX (X)	XX (X)	XX (X)	XX (X)	XX (X)	XX (X)	XX (X)	XX (X)	XX (X)
Diastolic BP mmHg)	XX	XX (X)	XX (X)	XX (X)	XX (X)	XX (X)	XX (X)	XX (X)	XX (X)	XX (X)
Atrial fibrillation	XX	XX (X)	XX (X)	XX (X)	*XX (X)*	*XX (X)*	*XX (X)*	*XX (X)*	*XX (X)*	*XX (X)*
NIHSS (/42) †	XX	XX (X)	XX (X)	XX (X)	XX (X)	XX (X)	XX (X)	XX (X)	XX (X)	XX (X)
Weakness, Side (%)										
right	XX	XX (X)	XX (X)	XX (X)	XX (X)	XX (X)	XX (X)	XX (X)	XX (X)	XX (X)
left	XX	XX (X)	XX (X)	XX (X)	XX (X)	XX (X)	XX (X)	XX (X)	XX (X)	XX (X)
both	XX	XX (X)	XX (X)	XX (X)	XX (X)	XX (X)	XX (X)	XX (X)	XX (X)	XX (X)
Sensory loss (%)										
Right	XX	XX (X)	XX (X)	XX (X)	XX (X)	XX (X)	XX (X)	XX (X)	XX (X)	XX (X)
Left	XX	XX (X)	XX (X)	XX (X)	XX (X)	XX (X)	XX (X)	XX (X)	XX (X)	XX (X)
both	XX	XX (X)	XX (X)	XX (X)	XX (X)	XX (X)	XX (X)	XX (X)	XX (X)	XX (X)
Ataxia (%)										
left	XX	XX (X)	XX (X)	XX (X)	XX	XX (X)	XX (X)	XX (X)	XX	XX (X)
right	XX	XX (X)	XX (X)	XX (X)	XX	XX (X)	XX (X)	XX (X)	XX	XX (X)
Neglect/inattention (%)	XX	XX (X)	XX (X)	XX (X)	XX (X)	XX (X)	XX (X)	XX (X)	XX (X)	XX (X)
Dysphasia (%)	XX	XX (X)	XX (X)	XX (X)	XX (X)	XX (X)	XX (X)	XX (X)	XX (X)	XX (X)
Dysarthria (%)	XX	XX (X)	XX (X)	XX (X)	XX (X)	XX (X)	XX (X)	XX (X)	XX (X)	XX (X)
Visual loss (%)	XX	XX (X)	XX (X)	XX (X)	XX (X)	XX (X)	XX (X)	XX (X)	XX (X)	XX (X)
**Cognition**										
MoCA	XX	XX (X)	XX (X)	XX (X)	XX (X)	XX (X)	XX (X)	XX (X)	XX (X)	XX (X)
MoCA <=24	XX	XX (X)	XX (X)	XX (X)			XX (X)	XX (X)	XX (X)	
Verbal fluency F, <11 words	XX	XX (X)	XX (X)	XX (X)	XX (X)	XX (X)	XX (X)	XX (X)	XX (X)	XX (X)
Trails B, time	XX	XX (X)	XX (X)	XX (X)	XX (X)	XX (X)	XX (X)	XX (X)	XX (X)	XX (X)
Trails B, points	XX	XX (X)	XX (X)	XX (X)	XX (X)	XX (X)	XX (X)	XX (X)	XX (X)	XX (X)
**Investigations**										
CT scan	XX	XX (X)	XX (X)	XX (X)	XX (X)	XX (X)	XX (X)	XX (X)	XX (X)	XX (X)
MRI Scan	XX	XX (X)	XX (X)	XX (X)	XX (X)	XX (X)	XX (X)	XX (X)	XX (X)	XX (X)
Both scans	XX	XX (X)	XX (X)	XX (X)	XX (X)	XX (X)	XX (X)	XX (X)	XX (X)	XX (X)
Stroke-CT scan (days)	XX	XX (X)	XX (X)	XX (X)	XX (X)	XX (X)	XX (X)	XX (X)	XX (X)	XX (X)
Stroke-MRI scan (days)	XX	XX (X)	XX (X)	XX (X)	XX (X)	XX (X)	XX (X)	XX (X)	XX (X)	XX (X)

Index infarct present (%)	XX	XX (X)	XX (X)	XX (X)	XX (X)	XX (X)	XX (X)	XX (X)	XX (X)	XX (X)
Index infarct side, left (%)	XX	XX (X)	XX (X)	XX (X)	XX (X)	XX (X)	XX (X)	XX (X)	XX (X)	XX (X)
cSVD moderate/severe (%)	XX	XX (X)	XX (X)	XX (X)	XX (X)	XX (X)	XX (X)	XX (X)	XX (X)	XX (X)
WMH/ hypoattenuations	XX	XX (X)	XX (X)	XX (X)	XX (X)	XX (X)	XX (X)	XX (X)	XX (X)	XX (X)
Carotid stenosis	XX	XX (X)	XX (X)	XX (X)			XX (X)	XX (X)		
Left >=50%	XX	XX (X)	XX (X)	XX (X)	XX (X)	XX (X)	XX (X)	XX (X)	XX (X)	XX (X)
Right >=50%	XX	XX (X)	XX (X)	XX (X)	XX (X)	XX (X)	XX (X)	XX (X)	XX (X)	XX (X)
ECG, (%)										
Sinus	XX	XX (X)	XX (X)	XX (X)	XX (X)	XX (X)	XX (X)	XX (X)	XX (X)	XX (X)
AF	XX	XX (X)	XX (X)	XX (X)	XX (X)	XX (X)	XX (X)	XX (X)	XX (X)	XX (X)
Haemoglobin (g/l)	XX	XX (X)	XX (X)	XX (X)	XX (X)	XX (X)	XX (X)	XX (X)	XX (X)	XX (X)
Creatinine (µmol/l)	XX	XX (X)	XX (X)	XX (X)	XX (X)	XX (X)	XX (X)	XX (X)	XX (X)	XX (X)
eGFR (ml/min)	XX	XX (X)	XX (X)	XX (X)	XX (X)	XX (X)	XX (X)	XX (X)	XX (X)	XX (X)
**Contraindications to treatment**										
ISMN	XX	XX (X)	XX (X)	XX (X)	XX (X)	XX (X)	XX (X)	XX (X)	XX (X)	XX (X)
Cilostazol	XX	XX (X)	XX (X)	XX (X)	XX (X)	XX (X)	XX (X)	XX (X)	XX (X)	XX (X)

† Minimisation variable
ECG: electrocardiogram; F/T: full time; ICH: intracerebral haemorrhage; IS: ischaemic stroke; LACS: lacunar syndrome; LVH: left ventricular hypertrophy; PACS; partial anterior circulation syndrome; POCS: posterior circulation syndrome; P/T: part time; TACS: total anterior circulation syndrome; TIA: transient ischaemic attack

Table 2. Feasibility measures.
Data are number (%).

Measure	Metric	Achieved
Primary		
Recruitment	400 patients	363/400 (90.8%)
Retention of enrolees at 1 year	>95%	XX (X)
Secondary		
Tolerability	>=75% on at least half dose	XX (X)
ISMN alone		XX (X)
Cilostazol alone		XX (X)
Both ISMN and cilostazol		XX (X)
**Safety**		
Symptomatic extracranial bleeding		XX (X)
Symptomatic intracranial bleeding		XX (X)
Death	<2%	XX (X)
Stroke		XX (X)
Haemorrhage		XX (X)
Extracranial		XX (X)
Intracranial		XX (X)
**Efficacy**		XX (X)
Stroke		XX (X)
TIA		XX (X)
Myocardial infarction		XX (X)
Cognitive impairment		XX (X)
Dependency, mRS>2		XX (X)
Any of these	40-50%	XX (X)

Table 3. Clinical Outcomes at 12 months.
Data are number (%), median [interquartile range] or mean (standard deviation). Analyses performed using binary logistic regression (BLR), ordinal logistic regression (OLR) or multiple linear regression (MLR) with adjustment for age, sex, time from stroke onset to randomisation, years of education, smoking status, and baseline mRS (dependency), stroke severity (NIHSS) and systolic blood pressure. Mean (SD) and MLR will be used instead of median [IQR] and OLR for ordinal scales with more than 7 levels (central limit theorem/large sample). The Wei-Lachin test is used to analyse multiple outcomes in parallel.

Number	**ISMN**	**No ISMN**	**Difference (p)**	**Cilostazol**	**No cilostazol**	**Difference (p)**	**ISMN + Cil**	**Neither**	**Difference (p)**
**Composite**			CPHR			CPHR			CPHR
Stroke	XX (X)	XX (X)	BLR	XX (X)	XX (X)	BLR	XX (X)	XX (X)	BLR
TIA	XX (X)	XX (X)	BLR	XX (X)	XX (X)	BLR	XX (X)	XX (X)	BLR
MI	XX (X)	XX (X)	BLR	XX (X)	XX (X)	BLR	XX (X)	XX (X)	BLR
Cognitive impairment	XX (X)	XX (X)	BLR	XX (X)	XX (X)	BLR	XX (X)	XX (X)	BLR
Dependency, mRS>2	XX (X)	XX (X)	BLR	XX (X)	XX (X)	BLR	XX (X)	XX (X)	BLR
Death	XX (X)	XX (X)	BLR	XX (X)	XX (X)	BLR	XX (X)	XX (X)	BLR
**Cognition**									
Cognition, 7 level			OLR			OLR			OLR
Normal	XX (X)	XX (X)		XX (X)	XX (X)		XX (X)	XX (X)	
Minor, single domain	XX (X)	XX (X)		XX (X)	XX (X)		XX (X)	XX (X)	
Minor, multi-domain	XX (X)	XX (X)		XX (X)	XX (X)		XX (X)	XX (X)	
Major, mild	XX (X)	XX (X)		XX (X)	XX (X)		XX (X)	XX (X)	

Major, moderate	XX (X)	XX (X)		XX (X)	XX (X)		XX (X)	XX (X)	
Major, severe	XX (X)	XX (X)		XX (X)	XX (X)		XX (X)	XX (X)	
Death	XX (X)	XX (X)		XX (X)	XX (X)		XX (X)	XX (X)	
Cognition, 4 level			OLR			OLR			OLR
Normal	XX (X)	XX (X)		XX (X)	XX (X)		XX (X)	XX (X)	
Minor	XX (X)	XX (X)		XX (X)	XX (X)		XX (X)	XX (X)	
Dementia	XX (X)	XX (X)		XX (X)	XX (X)		XX (X)	XX (X)	
Death	XX (X)	XX (X)		XX (X)	XX (X)		XX (X)	XX (X)	
Memory/thinking problem	XX (X)	XX (X)	BLR	XX (X)	XX (X)	BLR	XX (X)	XX (X)	BLR
MoCA	XX (X)	XX (X)	MLR	XX (X)	XX (X)	MLR	XX (X)	XX (X)	MLR
TICS-m	XX (X)	XX (X)	MLR	XX (X)	XX (X)	MLR	XX (X)	XX (X)	MLR
Verbal fluency, animal naming	XX (X)	XX (X)	MLR	XX (X)	XX (X)	MLR	XX (X)	XX (X)	MLR
Trails B, time	XX (X)	XX (X)	MLR	XX (X)	XX (X)	MLR	XX (X)	XX (X)	MLR
Trails B, points	XX (X)	XX (X)	MLR	XX (X)	XX (X)	MLR	XX (X)	XX (X)	MLR
Dementia, clinical diagnosis	XX (X)	XX (X)	BLR	XX (X)	XX (X)	BLR	XX (X)	XX (X)	BLR
**Clinical**									
Systolic BP (mmHg)	XX (X)	XX (X)	MLR	XX (X)	XX (X)	MLR	XX (X)	XX (X)	MLR
Diastolic BP (mmHg)	XX (X)	XX (X)	MLR	XX (X)	XX (X)	MLR	XX (X)	XX (X)	MLR

Heart rate (bpm)	XX (X)	XX (X)	MLR	XX (X)	XX (X)	MLR	XX (X)	XX (X)	MLR
mRS	XX (X)	XX (X)	OLR	XX (X)	XX (X)	OLR	XX (X)	XX (X)	OLR
Disposition	XX (X)	XX (X)	OLR	XX (X)	XX (X)	OLR	XX (X)	XX (X)	OLR
ZDS	XX (X)	XX (X)	MLR	XX (X)	XX (X)	MLR	XX (X)	XX (X)	MLR
Clinical depression	XX (X)	XX (X)	BLR	XX (X)	XX (X)	BLR	XX (X)	XX (X)	BLR
EQ-5D-5L, as HU	XX (X)	XX (X)	MLR	XX (X)	XX (X)	MLR	XX (X)	XX (X)	MLR
EQ-VAS	XX (X)	XX (X)	MLR	XX (X)	XX (X)	MLR	XX (X)	XX (X)	MLR
SIS	XX (X)	XX (X)	MLR	XX (X)	XX (X)	MLR	XX (X)	XX (X)	MLR
Global	XX (X)	XX (X)	WLT	XX (X)	XX (X)	WLT	XX (X)	XX (X)	WLT

BP: blood pressure; MoCA: Montreal cognitive assessment-modified; mRS: modified Rankin Scale; SIS: stroke impact scale; TICS-m: Telephone interview cognitive status- modified; ZDS: Zung depression scale
Global: Recurrent ordinal stroke,[17] ordinal MI, cognition (MoCA), dependency (mRS), stroke impact scale, quality of life (EQ-5D).

Table 4. Adherence to medication with at least half dose or more by randomised group: isosorbide mononitrate (ISMN), cilostazol (Cil) and both together.

Week	ISMN	No ISMN	p	Cilostazol	No cilostazol	p	ISMN +Cil	ISMN alone	Cil only	Neither	p
1-2	XX (X)	XX (X)	Chi-sq	XX (X)	XX (X)	Chi-sq	XX (X)	XX (X)	XX (X)	XX (X)	Chi-sq
3-4	XX (X)	XX (X)	Chi-sq	XX (X)	XX (X)	Chi-sq	XX (X)	XX (X)	XX (X)	XX (X)	Chi-sq
26	XX (X)	XX (X)	Chi-sq	XX (X)	XX (X)	Chi-sq	XX (X)	XX (X)	XX (X)	XX (X)	Chi-sq
52	XX (X)	XX (X)	Chi-sq	XX (X)	XX (X)	Chi-sq	XX (X)	XX (X)	XX (X)	XX (X)	Chi-sq

A more detailed table by strata of drug adherence (i.e. 25%, 50%, 75% and 100% adherence) will also be prepared.

Table 5. Adjudicated baseline imaging characteristics by randomised group: isosorbide mononitrate (ISMN), cilostazol (Cil) and both together.
Data are number (%), median [IQR], or mean (standard deviation).

	N	All	ISMN	No ISMN	Cil	No Cil	ISMN + Cil	ISMN only	Cil only	None
Patients randomised	XX	XX	XX	XX	XX	XX	XX	XX	XX	XX
**Scan**										
Scan type(%)										
CT	XX	XX (X)	XX (X)	XX (X)	XX (X)	XX (X)	XX (X)	XX (X)	XX (X)	XX (X)
Time to scan (days)	XX	XX (X)	XX (X)	XX (X)	XX (X)	XX (X)	XX (X)	XX (X)	XX (X)	XX (X)
MR	XX	XX (X)	XX (X)	XX (X)	XX (X)	XX (X)	XX (X)	XX (X)	XX (X)	XX (X)
Time to scan (days)	XX	XX (X)	XX (X)	XX (X)	XX (X)	XX (X)	XX (X)	XX (X)	XX (X)	XX (X)
Scan quality (%)										
Good	XX	XX (X)	XX (X)	XX (X)	XX (X)	XX (X)	XX (X)	XX (X)	XX (X)	XX (X)
Moderate	XX	XX (X)	XX (X)	XX (X)	XX (X)	XX (X)	XX (X)	XX (X)	XX (X)	XX (X)
Poor	XX	XX (X)	XX (X)	XX (X)	XX (X)	XX (X)	XX (X)	XX (X)	XX (X)	XX (X)
**Index Lesion (i.e. main cause of stroke symptoms) (%)**										
Normal Scan	XX	XX (X)	XX (X)	XX (X)	XX (X)	XX (X)	XX (X)	XX (X)	XX (X)	XX (X)
Lesion present (type)										
Primary Acute ischaemia	XX	XX (X)	XX (X)	XX (X)	XX (X)	XX (X)	XX (X)	XX (X)	XX (X)	XX (X)
Primary haemorrhage	XX	XX (X)	XX (X)	XX (X)	XX (X)	XX (X)	XX (X)	XX (X)	XX (X)	XX (X)
Mimic	XX	XX (X)	XX (X)	XX (X)	XX (X)	XX (X)	XX (X)	XX (X)	XX (X)	XX (X)
No visible	XX	XX (X)	XX (X)	XX (X)	XX (X)	XX (X)	XX (X)	XX (X)	XX (X)	XX (X)
Infarct side of brain										
Right	XX	XX (X)	XX (X)	XX (X)	XX (X)	XX (X)	XX (X)	XX (X)	XX (X)	XX (X)
Left	XX	XX (X)	XX (X)	XX (X)	XX (X)	XX (X)	XX (X)	XX (X)	XX (X)	XX (X)
Both	XX	XX (X)	XX (X)	XX (X)	XX (X)	XX (X)	XX (X)	XX (X)	XX (X)	XX (X)
**Location (%)**										

	N	All	ISMN	No ISMN	Cil	No Cil	ISMN + Cil	ISMN only	Cil only	None
Index small subcortical (i.e. acute lacunar) infarct	XX	XX (X)	XX (X)	XX (X)	XX (X)	XX (X)	XX (X)	XX (X)	XX (X)	XX (X)
Internal capsule	XX	XX (X)	XX (X)	XX (X)	XX (X)	XX (X)	XX (X)	XX (X)	XX (X)	XX (X)
External capsule	XX	XX (X)	XX (X)	XX (X)	XX (X)	XX (X)	XX (X)	XX (X)	XX (X)	XX (X)
Lentiform nucleus	XX	XX (X)	XX (X)	XX (X)	XX (X)	XX (X)	XX (X)	XX (X)	XX (X)	XX (X)
Internal border zone	XX	XX (X)	XX (X)	XX (X)	XX (X)	XX (X)	XX (X)	XX (X)	XX (X)	XX (X)
Centrum semiovale	XX	XX (X)	XX (X)	XX (X)	XX (X)	XX (X)	XX (X)	XX (X)	XX (X)	XX (X)
Thalamus	XX	XX (X)	XX (X)	XX (X)	XX (X)	XX (X)	XX (X)	XX (X)	XX (X)	XX (X)
Lacunar- small deep cerebellar lesion	XX	XX (X)	XX (X)	XX (X)	XX (X)	XX (X)	XX (X)	XX (X)	XX (X)	XX (X)
Lacunar- small deep brainstem lesion (Pons)	XX	XX (X)	XX (X)	XX (X)	XX (X)	XX (X)	XX (X)	XX (X)	XX (X)	XX (X)
Lacunar - Medulla	XX	XX (X)	XX (X)	XX (X)	XX (X)	XX (X)	XX (X)	XX (X)	XX (X)	XX (X)
**Non-small subcortical (i.e. large artery cortical or large subcortical or posterior circulation) infarct**										
MCA territory	XX	XX (X)	XX (X)	XX (X)	XX (X)	XX (X)	XX (X)	XX (X)	XX (X)	XX (X)
PCA territory	XX	XX (X)	XX (X)	XX (X)	XX (X)	XX (X)	XX (X)	XX (X)	XX (X)	XX (X)
**Infarct size (mm)**										
A/P	XX	XX (X)	XX (X)	XX (X)	XX (X)	XX (X)	XX (X)	XX (X)	XX (X)	XX (X)
R/L	XX	XX (X)	XX (X)	XX (X)	XX (X)	XX (X)	XX (X)	XX (X)	XX (X)	XX (X)
Cranio-caudal	XX	XX (X)	XX (X)	XX (X)	XX (X)	XX (X)	XX (X)	XX (X)	XX (X)	XX (X)
**Microhaemorrhage (%)**										
Microhaemorrhages	XX	XX (X)	XX (X)	XX (X)	XX (X)	XX (X)	XX (X)	XX (X)	XX (X)	XX (X)
No. microhaemorrhages (%)										
1	XX	XX (X)	XX (X)	XX (X)	XX (X)	XX (X)	XX (X)	XX (X)	XX (X)	XX (X)
2	XX	XX (X)	XX (X)	XX (X)	XX (X)	XX (X)	XX (X)	XX (X)	XX (X)	XX (X)
3	XX	XX (X)	XX (X)	XX (X)	XX (X)	XX (X)	XX (X)	XX (X)	XX (X)	XX (X)
4	XX	XX (X)	XX (X)	XX (X)	XX (X)	XX (X)	XX (X)	XX (X)	XX (X)	XX (X)
>= 5	XX	XX (X)	XX (X)	XX (X)	XX (X)	XX (X)	XX (X)	XX (X)	XX (X)	XX (X)
Type of microhaemorrhages (%)										

	N	All	ISMN	No ISMN	Cil	No Cil	ISMN + Cil	ISMN only	Cil only	None
Lobar	XX	XX (X)	XX (X)	XX (X)	XX (X)	XX (X)	XX (X)	XX (X)	XX (X)	XX (X)
Deep	XX	XX (X)	XX (X)	XX (X)	XX (X)	XX (X)	XX (X)	XX (X)	XX (X)	XX (X)
Both	XX	XX (X)	XX (X)	XX (X)	XX (X)	XX (X)	XX (X)	XX (X)	XX (X)	XX (X)
Superficial siderosis present	XX	XX (X)	XX (X)	XX (X)	XX (X)	XX (X)	XX (X)	XX (X)	XX (X)	XX (X)
Siderosis focal	XX	XX (X)	XX (X)	XX (X)	XX (X)	XX (X)	XX (X)	XX (X)	XX (X)	XX (X)
Siderosis disseminated	XX	XX (X)	XX (X)	XX (X)	XX (X)	XX (X)	XX (X)	XX (X)	XX (X)	XX (X)
Siderosis location										
Left	XX	XX (X)	XX (X)	XX (X)	XX (X)	XX (X)	XX (X)	XX (X)	XX (X)	XX (X)
Right	XX	XX (X)	XX (X)	XX (X)	XX (X)	XX (X)	XX (X)	XX (X)	XX (X)	XX (X)
Both	XX	XX (X)	XX (X)	XX (X)	XX (X)	XX (X)	XX (X)	XX (X)	XX (X)	XX (X)
**Atrophy**										
Brain tissue volume reduction	XX	XX (X)	XX (X)	XX (X)	XX (X)	XX (X)	XX (X)	XX (X)	XX (X)	XX (X)
Central brain tissue volume										
Modest	XX	XX (X)	XX (X)	XX (X)	XX (X)	XX (X)	XX (X)	XX (X)	XX (X)	XX (X)
Severe	XX	XX (X)	XX (X)	XX (X)	XX (X)	XX (X)	XX (X)	XX (X)	XX (X)	XX (X)
Cortical brain tissue volume										
Modest	XX	XX (X)	XX (X)	XX (X)	XX (X)	XX (X)	XX (X)	XX (X)	XX (X)	XX (X)
Severe	XX	XX (X)	XX (X)	XX (X)	XX (X)	XX (X)	XX (X)	XX (X)	XX (X)	XX (X)
**White Matter hyperintensities (%)**										
White matter hyperintensities	XX	XX (X)	XX (X)	XX (X)	XX (X)	XX (X)	XX (X)	XX (X)	XX (X)	XX (X)
Anterior white matter lucency										
Restricted region adjoining ventricles	XX	XX (X)	XX (X)	XX (X)	XX (X)	XX (X)	XX (X)	XX (X)	XX (X)	XX (X)
Covering ventricle to cortex	XX	XX (X)	XX (X)	XX (X)	XX (X)	XX (X)	XX (X)	XX (X)	XX (X)	XX (X)
Posterior white matter lucency										
Restricted region adjoining ventricles	XX	XX (X)	XX (X)	XX (X)	XX (X)	XX (X)	XX (X)	XX (X)	XX (X)	XX (X)
Covering ventricle to cortex	XX	XX (X)	XX (X)	XX (X)	XX (X)	XX (X)	XX (X)	XX (X)	XX (X)	XX (X)
Anterior and/or Posterior white matter lucency										

	N	All	ISMN	No ISMN	Cil	No Cil	ISMN + Cil	ISMN only	Cil only	None
Restricted region adjoining ventricles	XX	XX (X)	XX (X)	XX (X)	XX (X)	XX (X)	XX (X)	XX (X)	XX (X)	XX (X)
Covering ventricle to cortex	XX	XX (X)	XX (X)	XX (X)	XX (X)	XX (X)	XX (X)	XX (X)	XX (X)	XX (X)
Periventricular WMH Fazekas score										
1	XX	XX (X)	XX (X)	XX (X)	XX (X)	XX (X)	XX (X)	XX (X)	XX (X)	XX (X)
2	XX	XX (X)	XX (X)	XX (X)	XX (X)	XX (X)	XX (X)	XX (X)	XX (X)	XX (X)
3	XX	XX (X)	XX (X)	XX (X)	XX (X)	XX (X)	XX (X)	XX (X)	XX (X)	XX (X)
Deep WMH Fazekas score										
1	XX	XX (X)	XX (X)	XX (X)	XX (X)	XX (X)	XX (X)	XX (X)	XX (X)	XX (X)
2	XX	XX (X)	XX (X)	XX (X)	XX (X)	XX (X)	XX (X)	XX (X)	XX (X)	XX (X)
3	XX	XX (X)	XX (X)	XX (X)	XX (X)	XX (X)	XX (X)	XX (X)	XX (X)	XX (X)
Periventricular and/or Deep WMH Fazekas score										
1	XX	XX (X)	XX (X)	XX (X)	XX (X)	XX (X)	XX (X)	XX (X)	XX (X)	XX (X)
2	XX	XX (X)	XX (X)	XX (X)	XX (X)	XX (X)	XX (X)	XX (X)	XX (X)	XX (X)
3	XX	XX (X)	XX (X)	XX (X)	XX (X)	XX (X)	XX (X)	XX (X)	XX (X)	XX (X)
**Enlarged perivascular spaces (%)**										
Enlarged perivascular spaces	XX	XX (X)	XX (X)	XX (X)	XX (X)	XX (X)	XX (X)	XX (X)	XX (X)	XX (X)
Basal ganglia rating, worse side										
<=10	XX	XX (X)	XX (X)	XX (X)	XX (X)	XX (X)	XX (X)	XX (X)	XX (X)	XX (X)
11-20	XX	XX (X)	XX (X)	XX (X)	XX (X)	XX (X)	XX (X)	XX (X)	XX (X)	XX (X)
20-40	XX	XX (X)	XX (X)	XX (X)	XX (X)	XX (X)	XX (X)	XX (X)	XX (X)	XX (X)
>40	XX	XX (X)	XX (X)	XX (X)	XX (X)	XX (X)	XX (X)	XX (X)	XX (X)	XX (X)
Centrum semiovale rating, worse side										
<=10	XX	XX (X)	XX (X)	XX (X)	XX (X)	XX (X)	XX (X)	XX (X)	XX (X)	XX (X)
11-20	XX	XX (X)	XX (X)	XX (X)	XX (X)	XX (X)	XX (X)	XX (X)	XX (X)	XX (X)
20-40	XX	XX (X)	XX (X)	XX (X)	XX (X)	XX (X)	XX (X)	XX (X)	XX (X)	XX (X)
>40	XX	XX (X)	XX (X)	XX (X)	XX (X)	XX (X)	XX (X)	XX (X)	XX (X)	XX (X)
**Old vascular lesions (%)**										

	N	All	ISMN	No ISMN	Cil	No Cil	ISMN + Cil	ISMN only	Cil only	None
Old vascular lesions	XX	XX (X)	XX (X)	XX (X)	XX (X)	XX (X)	XX (X)	XX (X)	XX (X)	XX (X)
Old cortical infarct	XX	XX (X)	XX (X)	XX (X)	XX (X)	XX (X)	XX (X)	XX (X)	XX (X)	XX (X)
Old striatocapsular infarct	XX	XX (X)	XX (X)	XX (X)	XX (X)	XX (X)	XX (X)	XX (X)	XX (X)	XX (X)
Old borderzone infarct	XX	XX (X)	XX (X)	XX (X)	XX (X)	XX (X)	XX (X)	XX (X)	XX (X)	XX (X)
Old lacunar infarct	XX	XX (X)	XX (X)	XX (X)	XX (X)	XX (X)	XX (X)	XX (X)	XX (X)	XX (X)
Number of lacunes (%)										
1	XX	XX (X)	XX (X)	XX (X)	XX (X)	XX (X)	XX (X)	XX (X)	XX (X)	XX (X)
2	XX	XX (X)	XX (X)	XX (X)	XX (X)	XX (X)	XX (X)	XX (X)	XX (X)	XX (X)
3	XX	XX (X)	XX (X)	XX (X)	XX (X)	XX (X)	XX (X)	XX (X)	XX (X)	XX (X)
4	XX	XX (X)	XX (X)	XX (X)	XX (X)	XX (X)	XX (X)	XX (X)	XX (X)	XX (X)
>=5	XX	XX (X)	XX (X)	XX (X)	XX (X)	XX (X)	XX (X)	XX (X)	XX (X)	XX (X)
Old brainstem/cerebellar infarcts	XX	XX (X)	XX (X)	XX (X)	XX (X)	XX (X)	XX (X)	XX (X)	XX (X)	XX (X)
Probable old haemorrhage	XX	XX (X)	XX (X)	XX (X)	XX (X)	XX (X)	XX (X)	XX (X)	XX (X)	XX (X)
**Non-stroke lesions (%)**										
Non-stroke lesion	XX	XX (X)	XX (X)	XX (X)	XX (X)	XX (X)	XX (X)	XX (X)	XX (X)	XX (X)
Classification of non-stroke (%)										
Cerebral tumour	XX	XX (X)	XX (X)	XX (X)	XX (X)	XX (X)	XX (X)	XX (X)	XX (X)	XX (X)
Aneurysm	XX	XX (X)	XX (X)	XX (X)	XX (X)	XX (X)	XX (X)	XX (X)	XX (X)	XX (X)
Vascular malformation	XX	XX (X)	XX (X)	XX (X)	XX (X)	XX (X)	XX (X)	XX (X)	XX (X)	XX (X)
Other non-stroke classification	XX	XX (X)	XX (X)	XX (X)	XX (X)	XX (X)	XX (X)	XX (X)	XX (X)	XX (X)

Table 6a. Serious adverse events for ISMN.
Data are number (%).

	All			Fatal		
Number	ISMN	No ISMN	Difference (p)	ISMN	No ISMN	Difference (p)
**Treatment**						
During	xx (xx.x)	xx (xx.x)	BLR	xx (xx.x)	xx (xx.x)	BLR
After	xx (xx.x)	xx (xx.x)	BLR			BLR
**Relationship**						
Possibly	xx (xx.x)	xx (xx.x)	Ch sq	xx (xx.x)	xx (xx.x)	Ch sq
Probably	xx (xx.x)	xx (xx.x)	-	xx (xx.x)	xx (xx.x)	-
Definitely	xx (xx.x)	xx (xx.x)	-	xx (xx.x)	xx (xx.x)	-

Table 6b. Serious adverse events for cilostazol.
Data are number (%).

	All			Fatal		
Number	Cilostazol	No Cilostazol	Difference (p)	Cilostazol	No Cilostazol	Difference (p)
**Treatment**						
During	xx (xx.x)	xx (xx.x)	BLR	xx (xx.x)	xx (xx.x)	BLR
After	xx (xx.x)	xx (xx.x)	BLR	xx (xx.x)	xx (xx.x)	BLR
**Relationship**						
Possibly	xx (xx.x)	xx (xx.x)	Ch sq	xx (xx.x)	xx (xx.x)	Ch sq
Probably	xx (xx.x)	xx (xx.x)	-	xx (xx.x)	xx (xx.x)	-
Definitely	xx (xx.x)	xx (xx.x)	-	xx (xx.x)	xx (xx.x)	-

Table 6c. Serious adverse events for combined ISMN and cilostazol.
Data are number (%).

	All			Fatal		
Number	ISMN /cil	No ISMN/cil	Difference (p)	ISMN /cil	No ISMN/cil	Difference (p)
**Treatment**						
During	xx (xx.x)	xx (xx.x)	BLR	xx (xx.x)	xx (xx.x)	BLR
After	xx (xx.x)	xx (xx.x)	BLR	xx (xx.x)	xx (xx.x)	BLR
**Relationship**						
Possibly	xx (xx.x)	xx (xx.x)	Ch sq	xx (xx.x)	xx (xx.x)	Ch sq
Probably	xx (xx.x)	xx (xx.x)	-	xx (xx.x)	xx (xx.x)	-
Definitely	xx (xx.x)	xx (xx.x)	-	xx (xx.x)	xx (xx.x)	-

Table 7a. Participants with at least one serious adverse events by organ randomised to isosorbide mononitrate (ISMN) versus none.
Data are number (%); comparison by binary logistic regression.

Number	All ISMN	All No ISMN	All Difference (p)	Fatal ISMN	Fatal No ISMN	Fatal Difference (p)
Cardiovascular	xx (xx.x)	xx (xx.x)	(xx.x, xx.x), p=0.xxx	xx (xx.x)	xx (xx.x)	(xx.x, xx.x), p=0.xxx
Myocardial infarction	xx (xx.x)	xx (xx.x)	(xx.x, xx.x), p=0.xxx	xx (xx.x)	xx (xx.x)	(xx.x, xx.x), p=0.xxx
Nervous system	xx (xx.x)	xx (xx.x)	(xx.x, xx.x), p=0.xxx	xx (xx.x)	xx (xx.x)	(xx.x, xx.x), p=0.xxx
Ischaemic stroke	xx (xx.x)	xx (xx.x)	(xx.x, xx.x), p=0.xxx	xx (xx.x)	xx (xx.x)	(xx.x, xx.x), p=0.xxx
Transient ischaemic attack	xx (xx.x)	xx (xx.x)	(xx.x, xx.x), p=0.xxx	xx (xx.x)	xx (xx.x)	(xx.x, xx.x), p=0.xxx
Intracerebral haemorrhage	xx (xx.x)	xx (xx.x)	(xx.x, xx.x), p=0.xxx	xx (xx.x)	xx (xx.x)	(xx.x, xx.x), p=0.xxx
Respiratory	xx (xx.x)	xx (xx.x)	(xx.x, xx.x), p=0.xxx	xx (xx.x)	xx (xx.x)	(xx.x, xx.x), p=0.xxx
Gastrointestinal	xx (xx.x)	xx (xx.x)	(xx.x, xx.x), p=0.xxx	xx (xx.x)	xx (xx.x)	(xx.x, xx.x), p=0.xxx
Genitourinary	xx (xx.x)	xx (xx.x)	(xx.x, xx.x), p=0.xxx	xx (xx.x)	xx (xx.x)	(xx.x, xx.x), p=0.xxx
Secondary	xx (xx.x)	xx (xx.x)	(xx.x, xx.x), p=0.xxx	xx (xx.x)	xx (xx.x)	(xx.x, xx.x), p=0.xxx
Haematological	xx (xx.x)	xx (xx.x)	(xx.x, xx.x), p=0.xxx	xx (xx.x)	xx (xx.x)	(xx.x, xx.x), p=0.xxx
Metabolic/endocrine	xx (xx.x)	xx (xx.x)	(xx.x, xx.x), p=0.xxx	xx (xx.x)	xx (xx.x)	(xx.x, xx.x), p=0.xxx
Musculoskeletal	xx (xx.x)	xx (xx.x)	(xx.x, xx.x), p=0.xxx	xx (xx.x)	xx (xx.x)	(xx.x, xx.x), p=0.xxx
Infection/sepsis	xx (xx.x)	xx (xx.x)	(xx.x, xx.x), p=0.xxx	xx (xx.x)	xx (xx.x)	(xx.x, xx.x), p=0.xxx
Tumour/malignancy	xx (xx.x)	xx (xx.x)	(xx.x, xx.x), p=0.xxx	xx (xx.x)	xx (xx.x)	(xx.x, xx.x), p=0.xxx
Other	xx (xx.x)	xx (xx.x)	(xx.x, xx.x), p=0.xxx	xx (xx.x)	xx (xx.x)	(xx.x, xx.x), p=0.xxx
Total	xx (xx.x)	xx (xx.x)	(xx.x, xx.x), p=0.xxx	xx (xx.x)	xx (xx.x)	(xx.x, xx.x), p=0.xxx

Table 7b. Participants with at least one serious adverse events by organ randomised to cilostazol (Cil) versus none.
Data are number (%); comparison by binary logistic regression.

	All			Fatal		
Number	Cil	No Cil	Difference (p)	Cil	No Cil	Difference (p)
Cardiovascular	xx (xx.x)	xx (xx.x)	(xx.x, xx.x), p=0.xxx	xx (xx.x)	xx (xx.x)	(xx.x, xx.x), p=0.xxx
Myocardial infarction	xx (xx.x)	xx (xx.x)	(xx.x, xx.x), p=0.xxx	xx (xx.x)	xx (xx.x)	(xx.x, xx.x), p=0.xxx
Nervous system	xx (xx.x)	xx (xx.x)	(xx.x, xx.x), p=0.xxx	xx (xx.x)	xx (xx.x)	(xx.x, xx.x), p=0.xxx
Ischaemic stroke	xx (xx.x)	xx (xx.x)	(xx.x, xx.x), p=0.xxx	xx (xx.x)	xx (xx.x)	(xx.x, xx.x), p=0.xxx
Transient ischaemic attack	xx (xx.x)	xx (xx.x)	(xx.x, xx.x), p=0.xxx	xx (xx.x)	xx (xx.x)	(xx.x, xx.x), p=0.xxx
Intracerebral haemorrhage	xx (xx.x)	xx (xx.x)	(xx.x, xx.x), p=0.xxx	xx (xx.x)	xx (xx.x)	(xx.x, xx.x), p=0.xxx
Respiratory	xx (xx.x)	xx (xx.x)	(xx.x, xx.x), p=0.xxx	xx (xx.x)	xx (xx.x)	(xx.x, xx.x), p=0.xxx
Gastrointestinal	xx (xx.x)	xx (xx.x)	(xx.x, xx.x), p=0.xxx	xx (xx.x)	xx (xx.x)	(xx.x, xx.x), p=0.xxx
Genitourinary	xx (xx.x)	xx (xx.x)	(xx.x, xx.x), p=0.xxx	xx (xx.x)	xx (xx.x)	(xx.x, xx.x), p=0.xxx
Secondary	xx (xx.x)	xx (xx.x)	(xx.x, xx.x), p=0.xxx	xx (xx.x)	xx (xx.x)	(xx.x, xx.x), p=0.xxx
Haematological	xx (xx.x)	xx (xx.x)	(xx.x, xx.x), p=0.xxx	xx (xx.x)	xx (xx.x)	(xx.x, xx.x), p=0.xxx
Metabolic/endocrine	xx (xx.x)	xx (xx.x)	(xx.x, xx.x), p=0.xxx	xx (xx.x)	xx (xx.x)	(xx.x, xx.x), p=0.xxx
Musculoskeletal	xx (xx.x)	xx (xx.x)	(xx.x, xx.x), p=0.xxx	xx (xx.x)	xx (xx.x)	(xx.x, xx.x), p=0.xxx
Infection/sepsis	xx (xx.x)	xx (xx.x)	(xx.x, xx.x), p=0.xxx	xx (xx.x)	xx (xx.x)	(xx.x, xx.x), p=0.xxx
Tumour/malignancy	xx (xx.x)	xx (xx.x)	(xx.x, xx.x), p=0.xxx	xx (xx.x)	xx (xx.x)	(xx.x, xx.x), p=0.xxx
Other	xx (xx.x)	xx (xx.x)	(xx.x, xx.x), p=0.xxx	xx (xx.x)	xx (xx.x)	(xx.x, xx.x), p=0.xxx
Total	xx (xx.x)	xx (xx.x)	(xx.x, xx.x), p=0.xxx	xx (xx.x)	xx (xx.x)	(xx.x, xx.x), p=0.xxx

Table 7c. Participants with at least one serious adverse events by organ randomised to combined isosorbide mononitrate (ISMN) and cilostazol (Cil) versus neither.
Data are number (%); comparison by binary logistic regression.

Number	All: ISMN /Cil	All: No ISNM /Cil	All: Difference (p)	Fatal: ISMN /Cil	Fatal: No ISNM /Cil	Fatal: Difference (p)
Cardiovascular	xx (xx.x)	xx (xx.x)	(xx.x, xx.x), p=0.xxx	xx (xx.x)	xx (xx.x)	(xx.x, xx.x), p=0.xxx
Myocardial infarction	xx (xx.x)	xx (xx.x)	(xx.x, xx.x), p=0.xxx	xx (xx.x)	xx (xx.x)	(xx.x, xx.x), p=0.xxx
Nervous system	xx (xx.x)	xx (xx.x)	(xx.x, xx.x), p=0.xxx	xx (xx.x)	xx (xx.x)	(xx.x, xx.x), p=0.xxx
Ischaemic stroke	xx (xx.x)	xx (xx.x)	(xx.x, xx.x), p=0.xxx	xx (xx.x)	xx (xx.x)	(xx.x, xx.x), p=0.xxx
Transient ischaemic attack	xx (xx.x)	xx (xx.x)	(xx.x, xx.x), p=0.xxx	xx (xx.x)	xx (xx.x)	(xx.x, xx.x), p=0.xxx
Intracerebral haemorrhage	xx (xx.x)	xx (xx.x)	(xx.x, xx.x), p=0.xxx	xx (xx.x)	xx (xx.x)	(xx.x, xx.x), p=0.xxx
Respiratory	xx (xx.x)	xx (xx.x)	(xx.x, xx.x), p=0.xxx	xx (xx.x)	xx (xx.x)	(xx.x, xx.x), p=0.xxx
Gastrointestinal	xx (xx.x)	xx (xx.x)	(xx.x, xx.x), p=0.xxx	xx (xx.x)	xx (xx.x)	(xx.x, xx.x), p=0.xxx
Genitourinary	xx (xx.x)	xx (xx.x)	(xx.x, xx.x), p=0.xxx	xx (xx.x)	xx (xx.x)	(xx.x, xx.x), p=0.xxx
Secondary	xx (xx.x)	xx (xx.x)	(xx.x, xx.x), p=0.xxx	xx (xx.x)	xx (xx.x)	(xx.x, xx.x), p=0.xxx
Haematological	xx (xx.x)	xx (xx.x)	(xx.x, xx.x), p=0.xxx	xx (xx.x)	xx (xx.x)	(xx.x, xx.x), p=0.xxx
Metabolic/endocrine	xx (xx.x)	xx (xx.x)	(xx.x, xx.x), p=0.xxx	xx (xx.x)	xx (xx.x)	(xx.x, xx.x), p=0.xxx
Musculoskeletal	xx (xx.x)	xx (xx.x)	(xx.x, xx.x), p=0.xxx	xx (xx.x)	xx (xx.x)	(xx.x, xx.x), p=0.xxx
Infection/sepsis	xx (xx.x)	xx (xx.x)	(xx.x, xx.x), p=0.xxx	xx (xx.x)	xx (xx.x)	(xx.x, xx.x), p=0.xxx
Tumour/malignancy	xx (xx.x)	xx (xx.x)	(xx.x, xx.x), p=0.xxx	xx (xx.x)	xx (xx.x)	(xx.x, xx.x), p=0.xxx
Other	xx (xx.x)	xx (xx.x)	(xx.x, xx.x), p=0.xxx	xx (xx.x)	xx (xx.x)	(xx.x, xx.x), p=0.xxx
Total	xx (xx.x)	xx (xx.x)	(xx.x, xx.x), p=0.xxx	xx (xx.x)	xx (xx.x)	(xx.x, xx.x), p=0.xxx

Table 8. Targeted symptoms occurring at any time on treatment on isosorbide mononitrate (ISMN), cilostazol (cil) or both.
Data are number (%); comparison by binary logistic regression.

	ISMN	No ISMN	Difference (p)	Cil	No cil	Difference (p)	ISMN /Cil	No ISMN /Cil	Difference (p)
Headache	xx (xx.x)	xx (xx.x)	(xx.x, xx.x), p=0.xxx	xx (xx.x)	xx (xx.x)	(xx.x, xx.x), p=0.xxx	xx (xx.x)	xx (xx.x)	(xx.x, xx.x), p=0.xxx
Stopped normal activities	xx (xx.x)	xx (xx.x)	(xx.x, xx.x), p=0.xxx	xx (xx.x)	xx (xx.x)	(xx.x, xx.x), p=0.xxx	xx (xx.x)	xx (xx.x)	(xx.x, xx.x), p=0.xxx
Palpitations	xx (xx.x)	xx (xx.x)	(xx.x, xx.x), p=0.xxx	xx (xx.x)	xx (xx.x)	(xx.x, xx.x), p=0.xxx	xx (xx.x)	xx (xx.x)	(xx.x, xx.x), p=0.xxx
Stopped normal activities	xx (xx.x)	xx (xx.x)	(xx.x, xx.x), p=0.xxx	xx (xx.x)	xx (xx.x)	(xx.x, xx.x), p=0.xxx	xx (xx.x)	xx (xx.x)	(xx.x, xx.x), p=0.xxx
Dizziness	xx (xx.x)	xx (xx.x)	(xx.x, xx.x), p=0.xxx	xx (xx.x)	xx (xx.x)	(xx.x, xx.x), p=0.xxx	xx (xx.x)	xx (xx.x)	(xx.x, xx.x), p=0.xxx
Stopped normal activities	xx (xx.x)	xx (xx.x)	(xx.x, xx.x), p=0.xxx	xx (xx.x)	xx (xx.x)	(xx.x, xx.x), p=0.xxx	xx (xx.x)	xx (xx.x)	(xx.x, xx.x), p=0.xxx
Loose stools	xx (xx.x)	xx (xx.x)	(xx.x, xx.x), p=0.xxx	xx (xx.x)	xx (xx.x)	(xx.x, xx.x), p=0.xxx	xx (xx.x)	xx (xx.x)	(xx.x, xx.x), p=0.xxx
Stopped normal activities	xx (xx.x)	xx (xx.x)	(xx.x, xx.x), p=0.xxx	xx (xx.x)	xx (xx.x)	(xx.x, xx.x), p=0.xxx	xx (xx.x)	xx (xx.x)	(xx.x, xx.x), p=0.xxx
Nausea	xx (xx.x)	xx (xx.x)	(xx.x, xx.x), p=0.xxx	xx (xx.x)	xx (xx.x)	(xx.x, xx.x), p=0.xxx	xx (xx.x)	xx (xx.x)	(xx.x, xx.x), p=0.xxx
Stopped normal activities	xx (xx.x)	xx (xx.x)	(xx.x, xx.x), p=0.xxx	xx (xx.x)	xx (xx.x)	(xx.x, xx.x), p=0.xxx	xx (xx.x)	xx (xx.x)	(xx.x, xx.x), p=0.xxx
Bleeding	xx (xx.x)	xx (xx.x)	(xx.x, xx.x), p=0.xxx	xx (xx.x)	xx (xx.x)	(xx.x, xx.x), p=0.xxx	xx (xx.x)	xx (xx.x)	(xx.x, xx.x), p=0.xxx
Stopped normal activities	xx (xx.x)	xx (xx.x)	(xx.x, xx.x), p=0.xxx	xx (xx.x)	xx (xx.x)	(xx.x, xx.x), p=0.xxx	xx (xx.x)	xx (xx.x)	(xx.x, xx.x), p=0.xxx
Bruising	xx (xx.x)	xx (xx.x)	(xx.x, xx.x), p=0.xxx	xx (xx.x)	xx (xx.x)	(xx.x, xx.x), p=0.xxx	xx (xx.x)	xx (xx.x)	(xx.x, xx.x), p=0.xxx
Stopped normal activities	xx (xx.x)	xx (xx.x)	(xx.x, xx.x), p=0.xxx	xx (xx.x)	xx (xx.x)	(xx.x, xx.x), p=0.xxx	xx (xx.x)	xx (xx.x)	(xx.x, xx.x), p=0.xxx
Falls	xx (xx.x)	xx (xx.x)	(xx.x, xx.x), p=0.xxx	xx (xx.x)	xx (xx.x)	(xx.x, xx.x), p=0.xxx	xx (xx.x)	xx (xx.x)	(xx.x, xx.x), p=0.xxx
Stopped normal activities	xx (xx.x)	xx (xx.x)	(xx.x, xx.x), p=0.xxx	xx (xx.x)	xx (xx.x)	(xx.x, xx.x), p=0.xxx	xx (xx.x)	xx (xx.x)	(xx.x, xx.x), p=0.xxx

Table 9. Adjudicated 1 year MRI imaging characteristics by randomised group: isosorbide mononitrate (ISMN), cilostazol (Cil) and both together.
Data are number (%), median [IQR], or mean (standard deviation).

	**N**	**All**	**ISMN**	**No ISMN**	**OR/MD/ HR (95%CI)**	**p-value**	**Cil**	**No Cil**	**OR/MD/ HR (95%CI)**	**p-value**	**ISMN + Cil**	**None**	**OR/MD/ HR (95%CI)**	**p-value**
Patients randomised	XX	XX	XX	XX			XX	XX			XX	XX		
**Scan**														
Time to scan (days)	XX	XX (X)	XX (X)	XX (X)	MLR	XX	XX (X)	XX (X)	MLR	XX	XX (X)	XX (X)	MLR	XX
Scan quality (%)					OLR	XX			OLR	XX			OLR	XX
Good	XX	XX (X)	XX (X)	XX (X)			XX (X)	XX (X)			XX (X)	XX (X)		
Moderate	XX	XX (X)	XX (X)	XX (X)			XX (X)	XX (X)			XX (X)	XX (X)		
Poor	XX	XX (X)	XX (X)	XX (X)			XX (X)	XX (X)			XX (X)	XX (X)		
**Appearance of the index infarct now (%)**														
Completely cavitated - visible on T2, FLAIR and T1"	XX	XX (X)	XX (X)	XX (X)	BLR	XX	XX (X)	XX (X)	BLR	XX	XX (X)	XX (X)	BLR	XX
Partially cavitated - lacy	XX	XX (X)	XX (X)	XX (X)	BLR	XX	XX (X)	XX (X)	BLR	XX	XX (X)	XX (X)	BLR	XX
Partially cavitated - hole + large WMH rim	XX	XX (X)	XX (X)	XX (X)	BLR	XX	XX (X)	XX (X)	BLR	XX	XX (X)	XX (X)	BLR	XX
Partially cavitated - FLAIR cavity but not=CSF	XX	XX (X)	XX (X)	XX (X)	BLR	XX	XX (X)	XX (X)	BLR	XX	XX (X)	XX (X)	BLR	XX
Partially cavitated - FLAIR=WMH T2=cavity	XX	XX (X)	XX (X)	XX (X)	BLR	XX	XX (X)	XX (X)	BLR	XX	XX (X)	XX (X)	BLR	XX
Partially cavitated - visible on T2 not FLAIR	XX	XX (X)	XX (X)	XX (X)	BLR	XX	XX (X)	XX (X)	BLR	XX	XX (X)	XX (X)	BLR	XX
Not cavitated (WML-like)	XX	XX (X)	XX (X)	XX (X)	BLR	XX	XX (X)	XX (X)	BLR	XX	XX (X)	XX (X)	BLR	XX
Disappeared	XX	XX (X)	XX (X)	XX (X)	BLR	XX	XX (X)	XX (X)	BLR	XX	XX (X)	XX (X)	BLR	XX
Become visible	XX	XX (X)	XX (X)	XX (X)	BLR	XX	XX (X)	XX (X)	BLR	XX	XX (X)	XX (X)	BLR	XX
Never visible	XX	XX (X)	XX (X)	XX (X)	BLR	XX	XX (X)	XX (X)	BLR	XX	XX (X)	XX (X)	BLR	XX
**Evidence of new stroke (%)**														

	N	All	ISMN	No ISMN	OR/MD/ HR (95%CI)	p-value	Cil	No Cil	OR/MD/ HR (95%CI)	p-value	ISMN + Cil	None	OR/MD/ HR (95%CI)	p-value
Ischaemic	XX	XX (X)	XX (X)	XX (X)	BLR	XX	XX (X)	XX (X)	BLR	XX	XX (X)	XX (X)	BLR	XX
Haemorrhagic	XX	XX (X)	XX (X)	XX (X)	BLR	XX	XX (X)	XX (X)	BLR	XX	XX (X)	XX (X)	BLR	XX
**Microhaemorrhages (%)**	XX	XX (X)	XX (X)	XX (X)	BLR	XX	XX (X)	XX (X)	BLR	XX	XX (X)	XX (X)	BLR	XX
No. microhaemorrhages (%)					OLR	XX			OLR	XX			OLR	XX
1	XX	XX (X)	XX (X)	XX (X)			XX (X)	XX (X)			XX (X)	XX (X)		
2	XX	XX (X)	XX (X)	XX (X)			XX (X)	XX (X)			XX (X)	XX (X)		
3	XX	XX (X)	XX (X)	XX (X)			XX (X)	XX (X)			XX (X)	XX (X)		
4	XX	XX (X)	XX (X)	XX (X)			XX (X)	XX (X)			XX (X)	XX (X)		
>= 5	XX	XX (X)	XX (X)	XX (X)			XX (X)	XX (X)			XX (X)	XX (X)		
Change from baseline	XX	XX [XX, XX]	XX [XX, XX]	XX [XX, XX]	OLR	XX	XX [XX, XX]	XX [XX, XX]	OLR	XX	XX [XX, XX]	XX [XX, XX]	OLR	XX
**Small vessel disease score**														
Total	XX	XX [XX, XX]	XX [XX, XX]	XX [XX, XX]	OLR	XX	XX [XX, XX]	XX [XX, XX]	OLR	XX	XX [XX, XX]	XX [XX, XX]	OLR	XX
Change from baseline	XX	XX [XX, XX]	XX [XX, XX]	XX [XX, XX]	OLR	XX	XX [XX, XX]	XX [XX, XX]	OLR	XX	XX [XX, XX]	XX [XX, XX]	OLR	XX
**Atrophy**														
Brain tissue volume reduction	XX	XX (X)	XX (X)	XX (X)	BLR	XX	XX (X)	XX (X)	BLR	XX	XX (X)	XX (X)	BLR	XX
Central brain tissue volume					OLR	XX			OLR	XX			OLR	XX
Modest	XX	XX (X)	XX (X)	XX (X)			XX (X)	XX (X)			XX (X)	XX (X)		
Severe	XX	XX (X)	XX (X)	XX (X)			XX (X)	XX (X)			XX (X)	XX (X)		
Cortical brain tissue volume					OLR	XX			OLR	XX			OLR	XX
Modest	XX	XX (X)	XX (X)	XX (X)			XX (X)	XX (X)			XX (X)	XX (X)		
Severe	XX	XX (X)	XX (X)	XX (X)			XX (X)	XX (X)			XX (X)	XX (X)		
Change from baseline					OLR	XX			OLR	XX			OLR	XX
More	XX	XX (X)	XX (X)	XX (X)			XX (X)	XX (X)			XX (X)	XX (X)		
Less	XX	XX (X)	XX (X)	XX (X)			XX (X)	XX (X)			XX (X)	XX (X)		
None	XX	XX (X)	XX (X)	XX (X)			XX (X)	XX (X)			XX (X)	XX (X)		

	N	All	ISMN	No ISMN	OR/MD/ HR (95%CI)	p-value	Cil	No Cil	OR/MD/ HR (95%CI)	p-value	ISMN + Cil	None	OR/MD/ HR (95%CI)	p-value
**White Matter hyperintensities (%)**														
White matter hyperintensities	XX	XX (X)	XX (X)	XX (X)	BLR	XX	XX (X)	XX (X)	BLR	XX	XX (X)	XX (X)	BLR	XX
Anterior white matter lucency														
Restricted region adjoining ventricles	XX	XX (X)	XX (X)	XX (X)	BLR	XX	XX (X)	XX (X)	BLR	XX	XX (X)	XX (X)	BLR	XX
Covering ventricle to cortex	XX	XX (X)	XX (X)	XX (X)	BLR	XX	XX (X)	XX (X)	BLR	XX	XX (X)	XX (X)	BLR	XX
Posterior white matter lucency														
Restricted region adjoining ventricles	XX	XX (X)	XX (X)	XX (X)	BLR	XX	XX (X)	XX (X)	BLR	XX	XX (X)	XX (X)	BLR	XX
Covering ventricle to cortex	XX	XX (X)	XX (X)	XX (X)	BLR	XX	XX (X)	XX (X)	BLR	XX	XX (X)	XX (X)	BLR	XX
Anterior and/or Posterior white matter lucency														
Restricted region adjoining ventricles	XX	XX (X)	XX (X)	XX (X)	BLR	XX	XX (X)	XX (X)	BLR	XX	XX (X)	XX (X)	BLR	XX
Covering ventricle to cortex	XX	XX (X)	XX (X)	XX (X)	BLR	XX	XX (X)	XX (X)	BLR	XX	XX (X)	XX (X)	BLR	XX
Periventricular WMH Fazekas score					OLR	XX			OLR	XX			OLR	XX
1	XX	XX (X)	XX (X)	XX (X)			XX (X)	XX (X)			XX (X)	XX (X)		
2	XX	XX (X)	XX (X)	XX (X)			XX (X)	XX (X)			XX (X)	XX (X)		
3	XX	XX (X)	XX (X)	XX (X)			XX (X)	XX (X)			XX (X)	XX (X)		
Change from baseline	XX	XX [XX, XX]	XX [XX, XX]	XX [XX, XX]	OLR	XX	XX [XX, XX]	XX [XX, XX]	OLR	XX	XX [XX, XX]	XX [XX, XX]	OLR	XX
Deep WMH Fazekas score					OLR	XX			OLR	XX			OLR	XX
1	XX	XX (X)	XX (X)	XX (X)			XX (X)	XX (X)			XX (X)	XX (X)		
2	XX	XX (X)	XX (X)	XX (X)			XX (X)	XX (X)			XX (X)	XX (X)		
3	XX	XX (X)	XX (X)	XX (X)			XX (X)	XX (X)			XX (X)	XX (X)		
Change from baseline	XX	XX [XX, XX]	XX [XX, XX]	XX [XX, XX]	OLR	XX	XX [XX, XX]	XX [XX, XX]	OLR	XX	XX [XX, XX]	XX [XX, XX]	OLR	XX
Periventricular and/or Deep WMH Fazekas score					OLR	XX			OLR	XX			OLR	XX
1	XX	XX (X)	XX (X)	XX (X)			XX (X)	XX (X)			XX (X)	XX (X)		
2	XX	XX (X)	XX (X)	XX (X)			XX (X)	XX (X)			XX (X)	XX (X)		

	N	All	ISMN	No ISMN	OR/MD/ HR (95%CI)	p-value	Cil	No Cil	OR/MD/ HR (95%CI)	p-value	ISMN + Cil	None	OR/MD/ HR (95%CI)	p-value
3	XX	XX (X)	XX (X)	XX (X)			XX (X)	XX (X)			XX (X)	XX (X)		
Change from baseline	XX	XX [XX, XX]	XX [XX, XX]	XX [XX, XX]	OLR	XX	XX [XX, XX]	XX [XX, XX]	OLR	XX	XX [XX, XX]	XX [XX, XX]	OLR	XX
WMH change from randomisation	XX	XX (X)	XX (X)	XX (X)	BLR	XX	XX (X)	XX (X)	BLR	XX	XX (X)	XX (X)	BLR	XX
Frontal (%)					OLR	XX			OLR	XX			OLR	XX
More	XX	XX (X)	XX (X)	XX (X)			XX (X)	XX (X)			XX (X)	XX (X)		
Less	XX	XX (X)	XX (X)	XX (X)			XX (X)	XX (X)			XX (X)	XX (X)		
No Change	XX	XX (X)	XX (X)	XX (X)			XX (X)	XX (X)			XX (X)	XX (X)		
Parietal (%)					OLR	XX			OLR	XX			OLR	XX
More	XX	XX (X)	XX (X)	XX (X)			XX (X)	XX (X)			XX (X)	XX (X)		
Less	XX	XX (X)	XX (X)	XX (X)			XX (X)	XX (X)			XX (X)	XX (X)		
No Change	XX	XX (X)	XX (X)	XX (X)			XX (X)	XX (X)			XX (X)	XX (X)		
Occipital (%)					OLR	XX			OLR	XX			OLR	XX
More	XX	XX (X)	XX (X)	XX (X)			XX (X)	XX (X)			XX (X)	XX (X)		
Less	XX	XX (X)	XX (X)	XX (X)			XX (X)	XX (X)			XX (X)	XX (X)		
No Change	XX	XX (X)	XX (X)	XX (X)			XX (X)	XX (X)			XX (X)	XX (X)		
Basal ganglia (%)					OLR	XX			OLR	XX			OLR	XX
More	XX	XX (X)	XX (X)	XX (X)			XX (X)	XX (X)			XX (X)	XX (X)		
Less	XX	XX (X)	XX (X)	XX (X)			XX (X)	XX (X)			XX (X)	XX (X)		
No Change	XX	XX (X)	XX (X)	XX (X)			XX (X)	XX (X)			XX (X)	XX (X)		
Posterior fossa (%)					OLR	XX			OLR	XX			OLR	XX
More	XX	XX (X)	XX (X)	XX (X)			XX (X)	XX (X)			XX (X)	XX (X)		
Less	XX	XX (X)	XX (X)	XX (X)			XX (X)	XX (X)			XX (X)	XX (X)		
No Change	XX	XX (X)	XX (X)	XX (X)			XX (X)	XX (X)			XX (X)	XX (X)		
**Old vascular lesions (%)**														
Old vascular lesions	XX	XX (X)	XX (X)	XX (X)	BLR	XX	XX (X)	XX (X)	BLR	XX	XX (X)	XX (X)	BLR	XX
Old cortical infarct	XX	XX (X)	XX (X)	XX (X)	BLR	XX	XX (X)	XX (X)	BLR	XX	XX (X)	XX (X)	BLR	XX
Old striatocapsular infarct	XX	XX (X)	XX (X)	XX (X)	BLR	XX	XX (X)	XX (X)	BLR	XX	XX (X)	XX (X)	BLR	XX

	N	All	ISMN	No ISMN	OR/MD/ HR (95%CI)	p-value	Cil	No Cil	OR/MD/ HR (95%CI)	p-value	ISMN + Cil	None	OR/MD/ HR (95%CI)	p-value
Old borderzone infarct	XX	XX (X)	XX (X)	XX (X)	BLR	XX	XX (X)	XX (X)	BLR	XX	XX (X)	XX (X)	BLR	XX
Old lacunar infarct	XX	XX (X)	XX (X)	XX (X)	BLR	XX	XX (X)	XX (X)	BLR	XX	XX (X)	XX (X)	BLR	XX
Number of lacunes (%)					OLR	XX			OLR	XX			OLR	XX
1	XX	XX (X)	XX (X)	XX (X)			XX (X)	XX (X)			XX (X)	XX (X)		
2	XX	XX (X)	XX (X)	XX (X)			XX (X)	XX (X)			XX (X)	XX (X)		
3	XX	XX (X)	XX (X)	XX (X)			XX (X)	XX (X)			XX (X)	XX (X)		
4	XX	XX (X)	XX (X)	XX (X)			XX (X)	XX (X)			XX (X)	XX (X)		
>=5	XX	XX (X)	XX (X)	XX (X)			XX (X)	XX (X)			XX (X)	XX (X)		
Change in number of lacunes from baseline	XX	XX [XX, XX]	XX [XX, XX]	XX [XX, XX]	OLR	XX	XX [XX, XX]	XX [XX, XX]	OLR	XX	XX [XX, XX]	XX [XX, XX]	OLR	XX
Old brainstem/cerebellar infarcts	XX	XX (X)	XX (X)	XX (X)	BLR	XX	XX (X)	XX (X)	BLR	XX	XX (X)	XX (X)	BLR	XX
Probable old haemorrhage	XX	XX (X)	XX (X)	XX (X)	BLR	XX	XX (X)	XX (X)	BLR	XX	XX (X)	XX (X)	BLR	XX
**Non-stroke lesions (%)**														
Non-stroke lesion	XX	XX (X)	XX (X)	XX (X)	BLR	XX	XX (X)	XX (X)	BLR	XX	XX (X)	XX (X)	BLR	XX
Classification of non-stroke (%)														
Cerebral tumour	XX	XX (X)	XX (X)	XX (X)	BLR	XX	XX (X)	XX (X)	BLR	XX	XX (X)	XX (X)	BLR	XX
Aneurysm	XX	XX (X)	XX (X)	XX (X)	BLR	XX	XX (X)	XX (X)	BLR	XX	XX (X)	XX (X)	BLR	XX
Vascular malformation	XX	XX (X)	XX (X)	XX (X)	BLR	XX	XX (X)	XX (X)	BLR	XX	XX (X)	XX (X)	BLR	XX
Other non-stroke classification	XX	XX (X)	XX (X)	XX (X)	BLR	XX	XX (X)	XX (X)	BLR	XX	XX (X)	XX (X)	BLR	XX

Figures

1. CONSORT flowchart diagram
2. Forest plot of composite outcome (stroke, TIA, MI, cognitive impairment, dependency or death) by clinical and adjudicated imaging subgroups
3. Forest plot of 7-level ordinal cognition/dementia scale by clinical and adjudicated imaging subgroups
4. Forest plot of Wei-Lachin global outcome by clinical and adjudicated imaging subgroups
5. Forest plot of central imaging reads as per list for table 9 of absolute and change in imaging findings
6. Stacked distributions of 7-level ordinal cognition at 12 months
7. Stacked distributions of 4-level ordinal cognition at 12 months
8. Stacked distributions of mRS at 12 months
9. Graph of imaging outcomes at 12 months (based on Table 9) displaying new incident infarct or haemorrhage, change in WMH, microbleeds, atrophy by allocated group.

Figure 1. CONSORT diagram

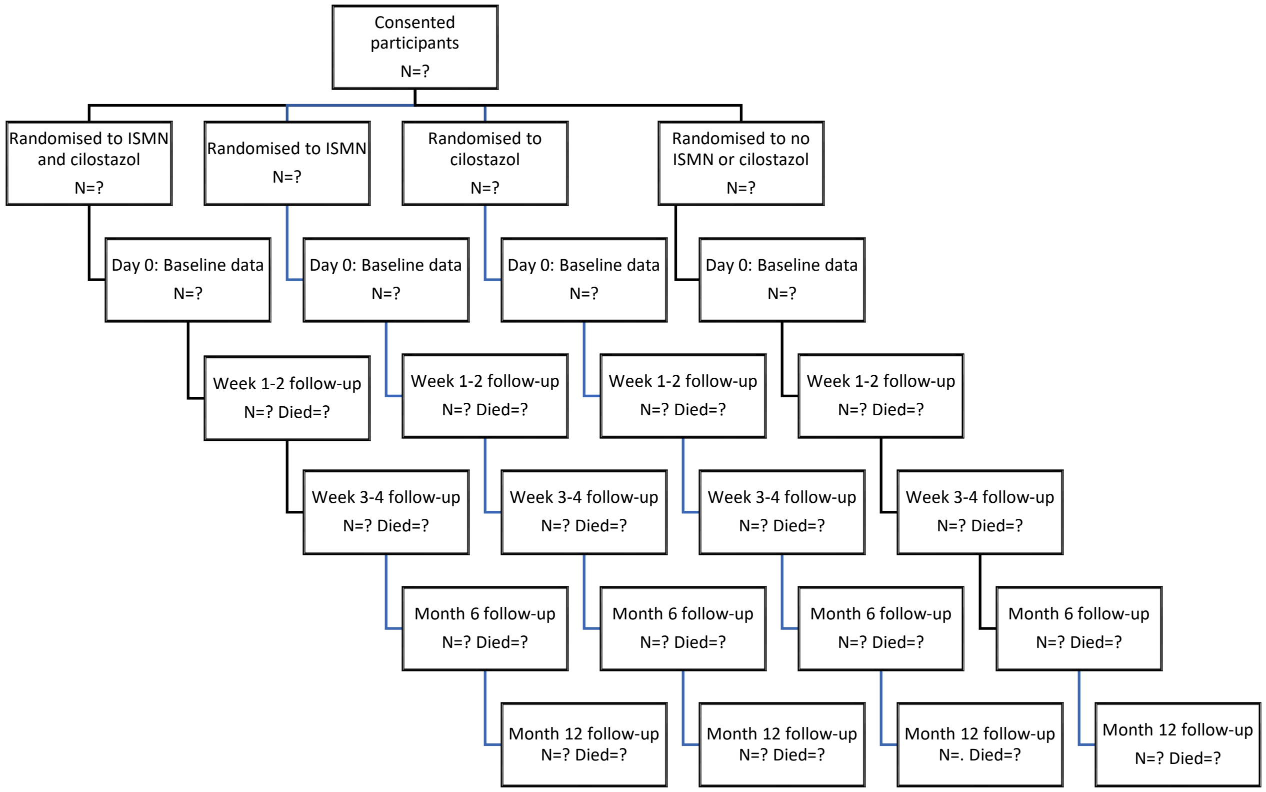

Figure 2a. Forest plot for isosorbide mononitrate versus none of composite outcome (stroke, TIA, MI, cognitive impairment, dependency or death) by clinical and adjudicated imaging subgroups. Adjusted

	Interaction p
Age †	
<60	-
60-69	-
≥70	-
Sex †	
Female	-
Male	-
Highest education †	
A-level (equivalent) or higher	-
O-level/GCSE (equivalent)	-
Primary or secondary primary school	-
Pre-morbid mRS †	
0	-
1	-
>1	-
Time stroke-baseline †	
<180 days	
180-364 days	
≥365 days	
Smoking	
Never	
Past	
Present	
Hypertension	
No	-
Yes	-
Diabetes mellitus	
No	-
Yes	-
History of stroke or TIA	
No	-
Yes	-
Systolic BP †	
<130	-
130-159	-
≥160	-
NIHSS †	
0/1	-
>1	-
Relevant acute infarct on imaging	
No	-
Yes	-
Lacune on imaging	
Not visible	-
Visible	-
WMH or hypoattenuation	
Not visible	-
Visible	-

SVD score	
0	-
1	-
>1	-

† Minimisation variable

Figure 2b. Forest plot for cilostazol versus none of composite outcome (stroke, TIA, MI, cognitive impairment, dependency or death) by clinical and adjudicated imaging subgroups. Adjusted

	Interaction p
Age †	
<60	-
60-69	-
≥70	-
Sex †	
Female	-
Male	-
Highest education †	
A-level (equivalent) or higher	-
O-level/GCSE (equivalent)	-
Primary or secondary primary school	-
Pre-morbid mRS †	
0	-
1	-
>1	-
Time stroke-baseline †	
<180 days	
180-364 days	
≥365 days	
Smoking	
Never	
Past	
Present	
Hypertension	
No	-
Yes	-
Diabetes mellitus	
No	-
Yes	-
History of stroke or TIA	
No	-
Yes	-
Systolic BP †	
<130	-
130-159	-
≥160	-
NIHSS †	
0/1	-
>1	-
Relevant acute infarct on imaging	
No	-
Yes	-
Lacune on imaging	
Not visible	-
Visible	-
WMH or hypoattenuation	
Not visible	-
Visible	-
SVD score	

0	-
1	-
>1	-

† Minimisation variable

Figure 2c. Forest plot for combined isosorbide mononitrate and cilostazol versus neither of composite outcome (stroke, TIA, MI, cognitive impairment, dependency or death) by clinical and adjudicated imaging subgroups. Adjusted

	Interaction p
Age †	
<60	-
60-69	-
≥70	-
Sex †	
Female	-
Male	-
Highest education †	
A-level (equivalent) or higher	-
O-level/GCSE (equivalent)	-
Primary or secondary primary school	-
Pre-morbid mRS †	
0	-
1	-
>1	-
Time stroke-baseline †	
<180 days	
180-364 days	
≥365 days	
Smoking	
Never	
Past	
Present	
Hypertension	
No	-
Yes	-
Diabetes mellitus	
No	-
Yes	-
History of stroke or TIA	
No	-
Yes	-
Systolic BP †	
<130	-
130-159	-
≥160	-
NIHSS †	
0/1	-
>1	-
Relevant acute infarct on imaging	
No	-
Yes	-
Lacune on imaging	
Not visible	-
Visible	-
WMH or hypoattenuation	
Not visible	-
Visible	-
SVD score	

0	-
1	-
>1	-

† Minimisation variable

Figure 3a. Forest plot of 7 level cognition scale for isosorbide mononitrate versus none at 12 months by clinical and adjudicated imaging subgroups. Adjusted

	Interaction p
Age †	
<60	-
60-69	-
≥70	-
Sex †	
Female	-
Male	-
Highest education †	
A-level (equivalent) or higher	-
O-level/GCSE (equivalent)	-
Primary or secondary primary school	-
Pre-morbid mRS †	
0	-
1	-
>1	-
Time stroke-baseline †	
<180 days	
180-364 days	
≥365 days	
Smoking	
Never	
Past	
Present	
Hypertension	
No	-
Yes	-
Diabetes mellitus	
No	-
Yes	-
History of stroke or TIA	
No	-
Yes	-
Systolic BP †	
<130	-
130-159	-
≥160	-
NIHSS †	
0/1	-
>1	-
Relevant acute infarct on imaging	
No	-
Yes	-
Lacune on imaging	
Not visible	-
Visible	-
WMH or hypoattenuation	
Not visible	-
Visible	-
SVD score	

0	-
1	-
>1	-

† Adjustment variable

Figure 3b. Forest plot of 7 level cognition scale for cilostazol versus none at 12 months by clinical and adjudicated imaging subgroups. Adjusted

	Interaction p
Age †	
<60	-
60-69	-
≥70	-
Sex †	
Female	-
Male	-
Highest education †	
A-level (equivalent) or higher	-
O-level/GCSE (equivalent)	-
Primary or secondary primary school	-
Pre-morbid mRS †	
0	-
1	-
>1	-
Time stroke-baseline †	
<180 days	
180-364 days	
≥365 days	
Smoking	
Never	
Past	
Present	
Hypertension	
No	-
Yes	-
Diabetes mellitus	
No	-
Yes	-
History of stroke or TIA	
No	-
Yes	-
Systolic BP †	
<130	-
130-159	-
≥160	-
NIHSS †	
0/1	-
>1	-
Relevant acute infarct on imaging	
No	-
Yes	-
Lacune on imaging	
Not visible	-
Visible	-
WMH or hypoattenuation	
Not visible	-
Visible	-
SVD score	

0	-
1	-
>1	-

† Adjustment variable

Figure 3c. Forest plot of 7 level cognition scale for combined isosorbide mononitrate and cilostazol versus neither at 12 months by baseline subgroups. Adjusted

	Interaction p
Age †	
<60	-
60-69	-
≥70	-
Sex †	
Female	-
Male	-
Highest education †	
A-level (equivalent) or higher	-
O-level/GCSE (equivalent)	-
Primary or secondary primary school	-
Pre-morbid mRS †	
0	-
1	-
>1	-
Time stroke-baseline †	
<180 days	
180-364 days	
≥365 days	
Smoking	
Never	
Past	
Present	
Hypertension	
No	-
Yes	-
Diabetes mellitus	
No	-
Yes	-
History of stroke or TIA	
No	-
Yes	-
Systolic BP †	
<130	-
130-159	-
≥160	-
NIHSS †	
0/1	-
>1	-
Relevant acute infarct on imaging	
No	-
Yes	-
Lacune on imaging	
Not visible	-
Visible	-
WMH or hypoattenuation	
Not visible	-
Visible	-
SVD score	

0	-
1	-
>1	-

† Adjustment variable

Figure 4a. Forest plot of Wei-Lachin global outcome for isosorbide mononitrate versus none by subgroups. Adjusted

	Interaction p
Age †	
<60	-
60-69	-
≥70	-
Sex †	
Female	-
Male	-
Highest education †	
A-level (equivalent) or higher	-
O-level/GCSE (equivalent)	-
Primary or secondary primary school	-
Pre-morbid mRS †	
0	-
1	-
>1	-
Time stroke-baseline †	
<180 days	
180-364 days	
≥365 days	
Smoking	
Never	
Past	
Present	
Hypertension	
No	-
Yes	-
Diabetes mellitus	
No	-
Yes	-
History of stroke or TIA	
No	-
Yes	-
Systolic BP †	
<130	-
130-159	-
≥160	-
NIHSS †	
0/1	-
>1	-
Relevant acute infarct on imaging	
No	-
Yes	-
Lacune on imaging	
Not visible	-
Visible	-
WMH or hypoattenuation	
Not visible	-
Visible	-
SVD score	

0	-
1	-
>1	-

† Adjustment variable

Figure 4b. Forest plot of Wei-Lachin global outcome for cilostazol versus none by subgroups. Adjusted

	Interaction p
Age †	
<60	-
60-69	-
≥70	-
Sex †	
Female	-
Male	-
Highest education †	
A-level (equivalent) or higher	-
O-level/GCSE (equivalent)	-
Primary or secondary primary school	-
Pre-morbid mRS †	
0	-
1	-
>1	-
Time stroke-baseline †	
<180 days	
180-364 days	
≥365 days	
Smoking	
Never	
Past	
Present	
Hypertension	
No	-
Yes	-
Diabetes mellitus	
No	-
Yes	-
History of stroke or TIA	
No	-
Yes	-
Systolic BP †	
<130	-
130-159	-
≥160	-
NIHSS †	
0/1	-
>1	-
Relevant acute infarct on imaging	
No	-
Yes	-
Lacune on imaging	
Not visible	-
Visible	-
WMH or hypoattenuation	
Not visible	-
Visible	-
SVD score	

0	-
1	-
>1	-

† Adjustment variable

Figure 4c. Forest plot of Wei-Lachin global outcome for combined isosorbide mononitrate and cilostazol versus neither by subgroups. Adjusted

	Interaction p
Age †	
<60	-
60-69	-
≥70	-
Sex †	
Female	-
Male	-
Highest education †	
A-level (equivalent) or higher	-
O-level/GCSE (equivalent)	-
Primary or secondary primary school	-
Pre-morbid mRS †	
0	-
1	-
>1	-
Time stroke-baseline †	
<180 days	
180-364 days	
≥365 days	
Smoking	
Never	
Past	
Present	
Hypertension	
No	-
Yes	-
Diabetes mellitus	
No	-
Yes	-
History of stroke or TIA	
No	-
Yes	-
Systolic BP †	
<130	-
130-159	-
≥160	-
NIHSS †	
0/1	-
>1	-
Relevant acute infarct on imaging	
No	-
Yes	-
Lacune on imaging	
Not visible	-
Visible	-
WMH or hypoattenuation	
Not visible	-
Visible	-
SVD score	

0	-
1	-
>1	-

† Adjustment variable

Figure 5. Forest plot of central imaging reads as per list for table 9 of absolute and change in imaging findings

a) Isosorbide mononitrate versus none
b) Cilostazol versus none
c) Combined isosorbide mononitrate and cilostazol versus neither

Figure 6. Stacked distributions of 7-level ordinal cognition at 12 months

d) Isosorbide mononitrate versus none
e) Cilostazol versus none
f) Combined isosorbide mononitrate and cilostazol versus neither

Figure 7. Stacked distributions of 4-level ordinal cognition at 12 months

a) Isosorbide mononitrate versus none
b) Cilostazol versus none
c) Combined isosorbide mononitrate and cilostazol versus neither

Figure 8. Stacked distributions of mRS at 12 months

a) Isosorbide mononitrate versus none
b) Cilostazol versus none
c) Combined isosorbide mononitrate and cilostazol versus neither

Figure 9. Graph of imaging outcomes at 12 months (based on Table 9) displaying new incident infarct or haemorrhage, change in WMH, microbleeds, atrophy by allocated group.

a) Isosorbide mononitrate versus none
b) Cilostazol versus none
c) Combined isosorbide mononitrate and cilostazol versus neither
