# Supplementary material for: Isosorbide Mononitrate and Cilostazol Treatment in Patients With Symptomatic Cerebral Small Vessel Disease: The Lacunar Intervention Trial-2 (LACI-2) Randomized Clinical Trial
Source: JAMA Neurol. 2023 May 24;80(7):682–92. doi: 10.1001/jamaneurol.2023.1526 (PMC10209826; doi:10.1001/jamaneurol.2023.1526)
Supplement: Supplement 2. — eMethods eTable 1. Adherence to Medication at Half Dose or More by Allocated Group: Isosorbide Mononitrate (ISMN), Cilostazol (Cil), and Both Drugs Together eTable 2. Serious Adverse Events (SAEs) by Timing and Relatedness to Allocated Treatment eTable 3. Serious Adverse Events (SAEs) by System eTable 4. Symptoms Related to Either Drug Occurring at Any Time eTable 5. Missing Data at 12 Months: Reasons and Distributions eTable 6. Comparison of Participants Without vs With Data on the Telephone Montreal Cognitive Assessment at 12 Months eFigure 1. Recruitment Graph eFigure 2. Modified Rankin Scale (mRS) at 12 Months, Ordinal Shift Distributions eFigure 3. Composite Outcome (Recurrent Stroke or TIA or MI, Any Cognitive Impairment, Dependency mRS >2, Death) by Subgroups eAppendix. Lacunar Intervention Trial-2 (LACI-2) Investigator Group eReferences [file jamaneurol-e231526-s002.pdf]

Supplementary Online Content

Wardlaw JM, Woodhouse LJ, Mhlanga II, et al; Lacunar Intervention Trial-2 (LACI-2) Investigator Group. Isosorbide mononitrate and cilostazol treatment in patients with symptomatic cerebral small vessel disease: the Lacunar Intervention Trial-2 (LACI-2) randomized clinical trial. *JAMA Neurol*. Published online May 24, 2023. doi:10.1001/jamaneurol.2023.1526

eMethods

eTable 1. Adherence to Medication at Half Dose or More by Allocated Group: Isosorbide Mononitrate (ISMN), Cilostazol (Cil), and Both Drugs Together

eTable 2. Serious Adverse Events (SAEs) by Timing and Relatedness to Allocated Treatment

eTable 3. Serious Adverse Events (SAEs) by System

eTable 4. Symptoms Related to Either Drug Occurring at Any Time

eTable 5. Missing Data at 12 Months: Reasons and Distributions

eTable 6. Comparison of Participants Without vs With Data on the Telephone Montreal Cognitive Assessment at 12 Months

eFigure 1. Recruitment Graph

eFigure 2. Modified Rankin Scale (mRS) at 12 Months, Ordinal Shift Distributions

eFigure 3. Composite Outcome (Recurrent Stroke or TIA or MI, Any Cognitive Impairment, Dependency mRS >2, Death) by Subgroups

eAppendix. Lacunar Intervention Trial-2 (LACI-2) Investigator Group

eReferences

This supplementary material has been provided by the authors to give readers additional information about their work.

© 2023 Wardlaw JM et al. *JAMA Neurology*.

eMethods

Oversight: A Trial Management Group oversaw LACI-2 at the Edinburgh Clinical Trials Unit (Trial Manager, follow-up coordinators, day-to-day running), Centre for Clinical Brain Sciences (imaging data collection and assessment) and Nottingham Stroke Trials Unit (CRF programming, statistics, follow-up coordinators).

The Sponsor (The Academic and Clinical Central Office for Research and Development (ACCORD)) approved all trial procedures, monitored the trial and assessed adverse events according to Good Clinical Practice (GCP).

The Trial Steering Committee (TSC) reviewed trial progress six-monthly.

An independent Data Monitoring Committee (DMC) reviewed unmasked data 12-monthly and indicated whether the trial should continue or not. All committees are listed in the LACI-2 Investigator Appendix.

We published the trial protocol[1] and statistical analysis plan (SAP)[2,3] prior to completion of the final patient follow-up and data lock.

The trial randomization was suspended for four months between late March and July 2020 due to the UK Government restrictions imposed on research due to the response to the SARS-COVID-19 pandemic (Supplementary Figure 1). During the suspension, patients who were already randomized in the trial and in follow-up continued their follow-up by post and phone and continued to receive trial medication from hospital pharmacies by post. When randomization was allowed to re-open, many sites were slow to restart since research staff were deployed to other NHS services due to the ongoing COVID-19 pandemic burden on hospitals and patients were less able to attend hospitals due to COVID restrictions. Therefore, the recruitment rate never recovered to that seen prior to the pandemic.

Trial follow-up at 12 months was by masked follow-up co-ordinators at the Clinical Trial Unit (University of Edinburgh) and the Stroke Trials Unit (University of Nottingham). All follow-up methods had been validated either by the original developers,[4,5] in the LACI-1 trial,[6,7] or by the LACI-2 Investigators in previous research.[8,9] Full details of follow-up procedures are described in the published protocol[1] and baseline paper which also includes the statistical analysis plan (SAP).[3]

In brief, at 12 months, participants were sent a validated postal questionnaire asking about recurrent stroke, MI, prescribed medications, smoking, the modified Rankin Scale, the Stroke Impact Scale, EQ 5D, and a masked question about trial tablet compliance. The named informant was sent the IQCODE. Separately, a masked co-ordinator phoned the participant and administered cognitive tests including the telephone Montreal Cognitive Assessment (tMoCA), Telephone Interview of Cognitive Status (TICS), and animal naming, and Zung questionnaire for mood. Up to two reminders were sent to participants or informants who did not respond to the initial questionnaire or phone call.

To assess self-reported tablet compliance, we used a carefully worded postal question, developed and tested in LACI-1, asking the participant a) 'how much of their trial medication they were taking currently' with options for responses of 'all', 'about three quarters', 'about half, 'about a quarter' or 'not taking study tablets'. Patients randomised to neither ISMN or cilostazol were asked to tick 'all'. Then b) for how long they had been taking that amount with options ranging from one week to 6 months or more. The responses were returned to the LACI-2 trial office and entered in the LACI-2 database by a masked data manager, separately to the phone questions.

Participants returned to the local site for a one year MRI, Trails B and blood pressure.

Site staff recorded adverse events including recurrent vascular events throughout the trial whenever they occurred.

© 2023 Wardlaw JM et al. *JAMA Neurology*.

eTable 1. Adherence to Medication at Half Dose or More by Allocated Group: Isosorbide Mononitrate (ISMN), Cilostazol (Cil), and Both Drugs Together

Week	N	All	Any ISMN	Any Cil	Both I+C	ISMN only	Cil only
Number of patients randomised		**363**	**181**	**182**	**91**	**90**	**91**
**Week 26 follow-up**	**363**	**318 (87.6)**	**157 (86.7)**	**160 (87.9)**	**80 (87.9)**	**77 (85.6)**	**80 (87.9)**
..At least 1/2 dose	313	213 (68.1)	117 (75.0)	115 (73.7)	54 (68.4)	63 (81.8)	61 (79.2)
**Week 52 follow-up**	**363**	**317 (87.3)**	**156 (86.2)**	**154 (84.6)**	**79 (86.8)**	**77 (85.6)**	**75 (82.4)**
..At least 1/2 dose	314	188 (59.9)	104 (67.5)	93 (60.8)	45 (57.7)	59 (77.6)	48 (64.0)

© 2023 Wardlaw JM et al. *JAMA Neurology*.

eTable 2. Serious Adverse Events (SAEs) by Timing and Relatedness to Allocated Treatment

	**All**	**All**			**Fatal**	**Fatal**		
**a) ISMN**	**All N=363**	**ISMN**	**No ISMN**	**p**	**All N=363**	**ISMN N=181**	**No ISMN N=182**	**p**
Number of SAEs	62	27	35		3	1	2	
Time related to treatment								
Before	1 (1.6)	1 (3.7)	0 (0.0)	-	0 (0.0)	0 (0.0)	0 (0.0)	-
During	43 (69.4)	20 (74.1)	23 (65.7)	0.48	1 (33.3)	0 (0.0)	1 (50.0)	-
After	14 (22.6)	4 (14.8)	10 (28.6)	0.21	2 (66.7)	1 (100.0)	1 (50.0)	1.00
Number of SAEs thought to be related to treatment								
Possibly	3 (4.8)	1 (3.7)	2 (5.7)	0.79	0 (0.0)	0 (0.0)	0 (0.0)	-
Probably	1 (1.6)	1 (3.7)	0 (0.0)	-	0 (0.0)	0 (0.0)	0 (0.0)	-
Definitely	0 (0.0)	0 (0.0)	0 (0.0)	-	0 (0.0)	0 (0.0)	0 (0.0)	-

**b) Cilostazol**	**All N=363**	**Cil N=182**	**No Cil N=181**	**p**	**All N=363**	**Cil N=182**	**No Cil N=181**	**p**
Number of SAEs	62	38	24		3	1	2	
Time related to treatment								
Before	1 (1.6)	1 (2.6)	0 (0.0)	-	0 (0.0)	0 (0.0)	0 (0.0)	-
During	43 (69.4)	26 (68.4)	17 (70.8)	0.84	1 (33.3)	0 (0.0)	1 (50.0)	-
After	14 (22.6)	9 (23.7)	5 (20.8)	0.79	2 (66.7)	1 (100.0)	1 (50.0)	1.00
Number of SAEs thought to be related to treatment								
Possibly	4 (6.5)	2 (5.3)	2 (8.3)	0.75	0 (0.0)	0 (0.0)	0 (0.0)	-
Probably	1 (1.6)	1 (2.6)	0 (0.0)	-	0 (0.0)	0 (0.0)	0 (0.0)	-
Definitely	0 (0.0)	0 (0.0)	0 (0.0)	-	0 (0.0)	0 (0.0)	0 (0.0)	-

**c) ISMN + Cilostazol**	**All N=182**	**ISMN+Cil N=91**	**None N=91**	**p**	**All N=182**	**ISMN+Cil N=91**	**None N=91**	**p**
Number of SAEs	33	18	15		1		1	
Time related to treatment								
Before	1 (3.0)	1 (5.6)	0 (0.0)	-	0 (0.0)		0 (0.0)	-
During	23 (69.7)	13 (72.2)	10 (66.7)	0.73	1 (100.0)		1 (100.0)	
After	7 (21.2)	3 (16.7)	4 (26.7)	0.49	0 (0.0)		0 (0.0)	-
Number of SAEs thought to be related to treatment								
Possibly	4 (12.1)	2 (11.1)	2 (13.3)	0.83	0 (0.0)		0 (0.0)	-
Probably	1 (3.0)	1 (5.6)	0 (0.0)	-	0 (0.0)		0 (0.0)	-
Definitely	0 (0.0)	0 (0.0)	0 (0.0)	-	0 (0.0)		0 (0.0)	-

© 2023 Wardlaw JM et al. *JAMA Neurology*.

eTable 3. Serious Adverse Events (SAEs) by System

The total number is the number of patients with an event, and a patient may have more than one type of event. There were three fatal SAEs, 1 cardiovascular, 1 respiratory and 1 genito-urinary.

A) ISMN

	All	All				Fatal	Fatal			
**ISMN**	**Total**	**ISMN**	**No ISMN**	**OR (95% CI)**	**p**	**Total**	**ISMN**	**No ISMN**	**OR (95% CI)**	**p**
**No. patients**	**363**	**181**	**182**			**363**	**181**	**182**		
Patients with an event	47 (12.9)	21 (11.6)	26 (14.3)	0.79 (0.43, 1.46)	0.45	3 (0.8)	1 (0.6)	2 (1.1)	0.50 (0.04, 5.56)	0.57
**By class**										
Cardiovascular	10 (2.8)	5 (2.8)	5 (2.7)	1.01 (0.29, 3.53)	0.99	1 (0.3)	1 (0.6)	0 (0.0)	-	-
Nervous system	6 (1.7)	2 (1.1)	4 (2.2)	0.50 (0.09, 2.75)	0.42	0 (0.0)	0 (0.0)	0 (0.0)	-	-
Respiratory	3 (0.8)	1 (0.6)	2 (1.1)	0.50 (0.04, 5.56)	0.57	1 (0.3)	0 (0.0)	1 (0.5)	-	-
Gastrointestinal	11 (3.0)	7 (3.9)	4 (2.2)	1.79 (0.51, 6.22)	0.36	0 (0.0)	0 (0.0)	0 (0.0)	-	-
Genitourinary	5 (1.4)	1 (0.6)	4 (2.2)	0.25 (0.03, 2.23)	0.21	1 (0.3)	0 (0.0)	1 (0.5)	-	-
Hematological	2 (0.6)	1 (0.6)	1 (0.5)	1.01 (0.06, 16.20)	1.00	0 (0.0)	0 (0.0)	0 (0.0)	-	-
Metabolic/Endocrine	0 (0.0)	0 (0.0)	0 (0.0)	-	-	0 (0.0)	0 (0.0)	0 (0.0)	-	-
Musculoskeletal	2 (0.6)	1 (0.6)	1 (0.5)	1.01 (0.06, 16.20)	1.00	0 (0.0)	0 (0.0)	0 (0.0)	-	-
Other	12 (3.3)	5 (2.8)	7 (3.8)	0.71 (0.22, 2.28)	0.57	0 (0.0)	0 (0.0)	0 (0.0)	-	-
**Events by grouping**										
Infection/sepsis[1]	5 (1.4)	2 (1.1)	3 (1.6)	0.67 (0.11, 4.04)	0.66	1 (0.3)	0 (0.0)	1 (0.5)	-	-
Tumor/ malignancy[2]	5 (1.4)	1 (0.6)	4 (2.2)	0.25 (0.03, 2.23)	0.21	1 (0.3)	0 (0.0)	1 (0.5)	-	-

[1]The infection was respiratory. [2] The tumor was genitourinary.

eTable 3. Serious Adverse Events (SAEs) by System

See explanatory notes above.

B): Cilostazol

	All	All				Fatal	Fatal			
**Cilostazol**	**Total**	**Cil**	**No Cil**	**OR (95% CI)**	**p**	**Total**	**Cil**	**No Cil**	**OR (95% CI)**	**p**
**No. patients**	**363**	**182**	**181**			**363**	**182**	**181**		
Patients with an event	47 (12.9)	29 (15.9)	18 (9.9)	1.72 (0.92, 3.22)	0.092	3 (0.8)	1 (0.5)	2 (1.1)	0.49 (0.04, 5.50)	0.57
**By class**										
Cardiovascular	10 (2.8)	7 (3.8)	3 (1.7)	2.37 (0.51, 6.15)	0.22	1 (0.3)	0 (0.0)	1 (0.6)	-	-
Nervous system	6 (1.7)	2 (1.1)	4 (2.2)	0.49 (0.09, 2.72)	0.42	0 (0.0)	0 (0.0)	0 (0.0)	-	-
Respiratory	3 (0.8)	2 (1.1)	1 (0.6)	2.00 (0.18, 22.25)	0.57	1 (0.3)	0 (0.0)	1 (0.6)	-	-
Gastrointestinal	11 (3.0)	8 (4.4)	3 (1.7)	2.73 (0.71, 10.45)	0.14	0 (0.0)	0 (0.0)	0 (0.0)	-	-
Genitourinary	5 (1.4)	4 (2.2)	1 (0.6)	4.04 (0.45, 36.54)	0.21	1 (0.3)	1 (0.5)	0 (0.0)	-	-
Hematological	2 (0.6)	0 (0.0)	2 (1.1)	-	-	0 (0.0)	0 (0.0)	0 (0.0)	-	-
Metabolic/Endocrine	0 (0.0)	0 (0.0)	0 (0.0)	-	-	0 (0.0)	0 (0.0)	0 (0.0)	-	-
Musculoskeletal	2 (0.6)	1 (0.5)	1 (0.6)	0.99 (0.06, 16.02)	1.00	0 (0.0)	0 (0.0)	0 (0.0)	-	-
Other	12 (3.3)	6 (3.3)	6 (3.3)	0.99 (0.31, 3.14)	0.99	0 (0.0)	0 (0.0)	0 (0.0)	-	-
**Events**										
Infection/sepsis	5 (1.4)	4 (2.2)	1 (0.6)	4.04 (0.45, 36.54)	0.21	1 (0.3)	0 (0.0)	1 (0.6)	-	-
Tumor/ malignancy	5 (1.4)	4 (2.2)	1 (0.6)	4.04 (0.45, 36.54)	0.21	1 (0.3)	1 (0.5)	0 (0.0)	-	-

© 2023 Wardlaw JM et al. *JAMA Neurology*.

eTable 3. Serious Adverse Events (SAEs) by System

See explanatory notes above.

C): Both ISMN and Cilostazol

ISMN + Cilostazol	All Total	All ISMN+ Cil	All None	All OR (95% CI)	All p	Fatal Total	Fatal ISMN+ Cil	Fatal None	Fatal OR (95% CI)	Fatal p
**No. patients**	**182**	**91**	**91**			**182**	**91**	**91**		
Patients with an event	27 (14.8)	15 (16.5)	12 (13.2)	1.30 (0.57, 2.96)	0.53	1 (0.5)	0 (0.0)	1 (1.1)	-	-
**By class**										
Cardiovascular	6 (3.3)	4 (4.4)	2 (2.2)	2.05 (0.37, 11.46)	0.42	0 (0.0)	0 (0.0)	0 (0.0)	-	-
Nervous system	4 (2.2)	1 (1.1)	3 (3.3)	0.33 (0.03, 3.19)	0.34	0 (0.0)	0 (0.0)	0 (0.0)	-	-
Respiratory	2 (1.1)	1 (1.1)	1 (1.1)	1.00 (0.06, 16.23)	1.00	1 (0.5)	0 (0.0)	1 (1.1)	-	-
Gastrointestinal	6 (3.3)	5 (5.5)	1 (1.1)	5.23 (0.60, 45.70)	0.13	0 (0.0)	0 (0.0)	0 (0.0)	-	-
Genitourinary	2 (1.1)	1 (1.1)	1 (1.1)	1.00 (0.06, 16.23)	1.00	0 (0.0)	0 (0.0)	0 (0.0)	-	-
Hematological	1 (0.5)	0 (0.0)	1 (1.1)	-	-	0 (0.0)	0 (0.0)	0 (0.0)	-	-
Metabolic/Endocrine	0 (0.0)	0 (0.0)	0 (0.0)	-	-	0 (0.0)	0 (0.0)	0 (0.0)	-	-
Musculoskeletal	0 (0.0)	0 (0.0)	0 (0.0)	-	-	0 (0.0)	0 (0.0)	0 (0.0)	-	-
Other	7 (3.8)	3 (3.3)	4 (4.4)	0.74 (0.16, 3.41)	0.70	0 (0.0)	0 (0.0)	0 (0.0)	-	-
**Events**										
Infection/sepsis	3 (1.6)	2 (2.2)	1 (1.1)	2.02 (0.18, 22.71)	0.57	1 (0.5)	0 (0.0)	1 (1.1)	-	-
Tumor/ malignancy	2 (1.1)	1 (1.1)	1 (1.1)	1.00 (0.06, 16.23)	1.00	0 (0.0)	0 (0.0)	0 (0.0)	-	-

© 2023 Wardlaw JM et al. *JAMA Neurology*.

eTable 4. Symptoms Related to Either Drug Occurring at Any Time

Data are number (%); comparison by binary logistic regression.

Symptom	N	All	ISMN				Cilostazol				Combined			
			ISMN	No ISMN	OR (95% CI)	p	Cil	No Cil	OR (95% CI)	p	ISMN +Cil	None	OR (95% CI)	p
Patients with data	363	363	181	182			182	181			91	91		
**Headache**	363	230 (63.4)	128 (70.7)	102 (56.0)	1.89 (1.23, 2.92)	0.0039	117 (64.3)	113 (62.4)	1.08 (0.71, 1.66)	0.71	64 (70.3)	49 (53.8)	2.03 (1.10, 3.74)	0.023
--Stopped normal activities	363	73 (20.1)	44 (24.3)	29 (15.9)	1.69 (1.00, 2.86)	0.048	36 (19.8)	37 (20.4)	0.96 (0.57, 1.60)	0.88	23 (25.3)	16 (17.6)	1.59 (0.77, 3.25)	0.21
**Palpitations**	363	95 (26.2)	43 (23.8)	52 (28.6)	0.78 (0.49, 1.25)	0.30	53 (29.1)	42 (23.2)	1.36 (0.85, 2.18)	0.20	25 (27.5)	24 (26.4)	1.06 (0.55, 2.04)	0.87
--Stopped normal activities	363	31 (8.5)	15 (8.3)	16 (8.8)	0.94 (0.45, 1.96)	0.86	17 (9.3)	14 (7.7)	1.23 (0.59, 2.57)	0.58	9 (9.9)	8 (8.8)	1.14 (0.42, 3.10)	0.80
**Dizziness**	363	191 (52.6)	95 (52.5)	96 (52.7)	0.99 (0.66, 1.49)	0.96	92 (50.5)	99 (54.7)	0.85 (0.56, 1.28)	0.43	51 (56.0)	55 (60.4)	0.83 (0.46, 1.51)	0.55
--Stopped normal activities	363	77 (21.2)	36 (19.9)	41 (22.5)	0.85 (0.52, 1.41)	0.54	27 (14.8)	50 (27.6)	0.46 (0.27, 0.77)	0.0033	19 (20.9)	33 (36.3)	0.46 (0.24, 0.90)	0.023
**Loose stools**	363	191 (52.6)	92 (50.8)	99 (54.4)	0.87 (0.57, 1.31)	0.50	116 (63.7)	75 (41.4)	2.48 (1.63, 3.79)	<0.001	56 (61.5)	39 (42.9)	2.13 (1.18, 3.86)	0.012
--Stopped normal activities	363	52 (14.3)	17 (9.4)	35 (19.2)	0.44 (0.23, 0.81)	0.0086	33 (18.1)	19 (10.5)	1.89 (1.03, 3.46)	0.040	12 (13.2)	14 (15.4)	0.84 (0.36, 1.92)	0.67
**Nausea**	363	93 (25.6)	44 (24.3)	49 (26.9)	0.87 (0.54, 1.40)	0.57	49 (26.9)	44 (24.3)	1.15 (0.72, 1.84)	0.57	25 (27.5)	25 (27.5)	1.00 (0.52, 1.92)	1.00
--Stopped normal activities	363	35 (9.6)	16 (8.8)	19 (10.4)	0.83 (0.41, 1.67)	0.61	18 (9.9)	17 (9.4)	1.06 (0.53, 2.13)	0.87	9 (9.9)	10 (11.0)	0.89 (0.34, 2.30)	0.81
**Bleeding**	363	64 (17.6)	27 (14.9)	37 (20.3)	0.69 (0.40, 1.19)	0.18	29 (15.9)	35 (19.3)	0.79 (0.46, 1.36)	0.40	16 (17.6)	24 (26.4)	0.60 (0.29, 1.22)	0.15
--Stopped normal activities	363	4 (1.1)	1 (0.6)	3 (1.6)	0.33 (0.03, 3.22)	0.34	2 (1.1)	2 (1.1)	0.99 (0.14, 7.14)	1.00	1 (1.1)	2 (2.2)	0.49 (0.04, 5.55)	0.57
**Dyspepsia**	363	123 (33.9)	57 (31.5)	66 (36.3)	0.81 (0.52, 1.25)	0.34	61 (33.5)	62 (34.3)	0.97 (0.63, 1.49)	0.88	30 (33.0)	35 (38.5)	0.79 (0.43, 1.44)	0.44

© 2023 Wardlaw JM et al. *JAMA Neurology*.

			ISMN				Cilostazol				Combined			
**Symptom**	**N**	**All**	**ISMN**	**No ISMN**	**OR (95% CI)**	**p**	**Cil**	**No Cil**	**OR (95% CI)**	**p**	**ISMN +Cil**	**None**	**OR (95% CI)**	**p**
--Stopped normal activities	363	16 (4.4)	6 (3.3)	10 (5.5)	0.59 (0.21, 1.66)	0.32	9 (4.9)	7 (3.9)	1.29 (0.47, 3.55)	0.62	4 (4.4)	5 (5.5)	0.79 (0.21, 3.04)	0.73
**Bruising**	363	89 (24.5)	38 (21.0)	51 (28.0)	0.68 (0.42, 1.11)	0.12	45 (24.7)	44 (24.3)	1.02 (0.63, 1.65)	0.93	23 (25.3)	29 (31.9)	0.72 (0.38, 1.38)	0.33
--Stopped normal activities	363	4 (1.1)	1 (0.6)	3 (1.6)	0.33 (0.03, 3.22)	0.34	0 (0.0)	4 (2.2)	0.00 (0.00, .)	1.00	0 (0.0)	3 (3.3)	0.00 (0.00, .)	1.00
**Falls**	363	89 (24.5)	39 (21.5)	50 (27.5)	0.73 (0.45, 1.17)	0.19	43 (23.6)	46 (25.4)	0.91 (0.56, 1.46)	0.69	23 (25.3)	30 (33.0)	0.69 (0.36, 1.31)	0.25

© 2023 Wardlaw JM et al. *JAMA Neurology*.

eTable 5. Missing Data at 12 Months: Reasons and Distributions

A) Missing mRS

Key
Frequency
Percent
Row Pct
Col Pct

Table of person status on 12m postal form by treatment							
**person status**	**treatment**						
	**ISMN**	**None**	**Cil**	**None**	**Both**	**None**	**All**
**Refused**	5<br>4.20<br>15.63<br>25.00	5<br>4.20<br>15.63<br>33.33	7<br>5.88<br>21.88<br>33.33	3<br>2.52<br>9.38<br>21.43	2<br>1.68<br>6.25<br>20.00	0<br>0.00<br>0.00<br>0.00	10<br>8.40<br>31.25<br>28.57
**Lost**	8<br>6.72<br>17.02<br>40.00	5<br>4.20<br>10.64<br>33.33	7<br>5.88<br>14.89<br>33.33	6<br>5.04<br>12.77<br>42.86	5<br>4.20<br>10.64<br>50.00	3<br>2.52<br>6.38<br>75.00	13<br>10.92<br>27.66<br>37.14
**Withdrawn**	7<br>5.88<br>17.50<br>35.00	5<br>4.20<br>12.50<br>33.33	7<br>5.88<br>17.50<br>33.33	5<br>4.20<br>12.50<br>35.71	3<br>2.52<br>7.50<br>30.00	1<br>0.84<br>2.50<br>25.00	12<br>10.08<br>30.00<br>34.29
**Total**	20<br>16.81	15<br>12.61	21<br>17.65	14<br>11.76	10<br>8.40	4<br>3.36	35<br>29.41

© 2023 Wardlaw JM et al. *JAMA Neurology*.

eTable 5. Missing Data at 12 Months: Reasons and Distributions

B) Missing Cognition-7 level derived score

Key
Frequency<br>Percent<br>Row Pct<br>Col Pct

Table of person status at 12 months by treatment								
person status	treatment							
	ISMN	None	Cil	None	Both	None	All	
Missing cognitive outcome data	4<br>2.30<br>10.26<br>13.79	7<br>4.02<br>17.95<br>33.33	3<br>1.72<br>7.69<br>12.00	8<br>4.60<br>20.51<br>32.00	1<br>0.57<br>2.56<br>7.14	5<br>2.87<br>12.82<br>50.00	11<br>6.32<br>28.21<br>22.00	
Refused	10<br>5.75<br>22.73<br>34.48	3<br>1.72<br>6.82<br>14.29	6<br>3.45<br>13.64<br>24.00	7<br>4.02<br>15.91<br>28.00	4<br>2.30<br>9.09<br>28.57	1<br>0.57<br>2.27<br>10.00	13<br>7.47<br>29.55<br>26.00	
Lost	7<br>4.02<br>17.50<br>24.14	4<br>2.30<br>10.00<br>19.05	7<br>4.02<br>17.50<br>28.00	4<br>2.30<br>10.00<br>16.00	5<br>2.87<br>12.50<br>35.71	2<br>1.15<br>5.00<br>20.00	11<br>6.32<br>27.50<br>22.00	
Does not fit in algorithm for score*	1<br>0.57<br>12.50<br>3.45	1<br>0.57<br>12.50<br>4.76	1<br>0.57<br>12.50<br>4.00	1<br>0.57<br>12.50<br>4.00	1<br>0.57<br>12.50<br>7.14	1<br>0.57<br>12.50<br>10.00	2<br>1.15<br>25.00<br>4.00	
Withdrawn	7<br>4.02<br>16.28<br>24.14	6<br>3.45<br>13.95<br>28.57	8<br>4.60<br>18.60<br>32.00	5<br>2.87<br>11.63<br>20.00	3<br>1.72<br>6.98<br>21.43	1<br>0.57<br>2.33<br>10.00	13<br>7.47<br>30.23<br>26.00	
Total	29<br>16.67	21<br>12.07	25<br>14.37	25<br>14.37	14<br>8.05	10<br>5.75	50<br>28.74	

*One participant had t-MOCA = 21, TICS-M = 24 and mRS = 1; Another participant had t-MOCA = 20, TICS-M = 23 and mRS = 3;

© 2023 Wardlaw JM et al. *JAMA Neurology*.

eTable 6. Comparison of Participants Without vs With Data on the Telephone Montreal Cognitive Assessment at 12 Months

A) ISMN versus no ISMN.

		No 12m MOCA			With 12m MOCA			p
**Variable**	**N**	**All**	**ISMN**	**None**	**All**	**ISMN**	**None**	**No 12m MOCA vs with 12m MOCA**
N	.	56	31	25	307	150	157	
Age (yrs)*	363	63.0 [54.0, 68.5]	63.0 [53.0, 70.0]	63.0 [56.0, 67.0]	64.0 [57.0, 72.0]	64.0 [56.0, 72.0]	65.0 [57.0, 74.0]	0.055
Sex, females	363	12 (21.4)	6 (19.4)	6 (24.0)	100 (32.6)	48 (32.0)	52 (33.1)	0.038
mRS > 1	363	21 (37.5)	13 (41.9)	8 (32.0)	64 (20.8)	27 (18.0)	37 (23.6)	0.0078
Onset to randomisation (days)*	363	52.0 [11.0, 249.5]	54.0 [21.0, 125.0]	50.0 [7.0, 326.0]	82.0 [31.0, 244.0]	90.5 [40.0, 252.0]	79.0 [29.0, 241.0]	0.18
Age completing education (yrs)	363	16.0 [15.0, 17.0]	16.0 [15.0, 16.0]	16.0 [15.0, 18.0]	16.0 [15.0, 18.0]	16.0 [15.0, 18.0]	16.0 [15.0, 17.0]	0.094
NIHSS (/42)	363	1.0 [0.0, 2.0]	1.0 [0.0, 2.0]	1.0 [0.0, 2.0]	0.0 [0.0, 2.0]	0.0 [0.0, 2.0]	0.0 [0.0, 1.0]	0.13
MOCA total (/30)	362	23.0 [20.0, 27.0]	23.0 [19.0, 26.0]	24.0 [23.0, 27.0]	27.0 [24.0, 28.0]	26.0 [24.0, 28.0]	27.0 [24.0, 28.0]	<0.0001
MOCA ≤24	362	31 (56.4)	17 (56.7)	14 (56.0)	87 (28.3)	38 (25.3)	49 (31.2)	<0.0001
t-MOCA total (/22)	362	17.0 [14.0, 20.0]	17.0 [13.0, 20.0]	18.0 [15.0, 20.0]	19.0 [17.0, 21.0]	20.0 [17.0, 21.0]	19.0 [17.0, 21.0]	<0.0001
Trails B, time (seconds)	359	154.5 [105.0, 206.0]	149.0 [82.0, 200.0]	168.0 [108.0, 236.0]	102.0 [74.0, 158.0]	101.0 [73.0, 149.0]	105.0 [77.0, 166.0]	0.0010
Trail B, points	359	24.0 [15.0, 25.0]	24.0 [15.0, 25.0]	24.0 [18.0, 25.0]	25.0 [23.0, 25.0]	25.0 [23.0, 25.0]	25.0 [23.0, 25.0]	0.0053

Please note: Chi-square test was used for sex, mRS>1 and MOCA ≤ 24; Mann-Whitney U-test was used for all the continuous variables.

© 2023 Wardlaw JM et al. *JAMA Neurology*.

eTable 6. Comparison of Participants Without vs With Data on the Telephone Montreal Cognitive Assessment at 12 Months

B) Cilostazol versus no cilostazol

		No 12m MOCA			With 12m MOCA			p
**Variable**	**N**	**All**	**Cil**	**None**	**All**	**Cil**	**None**	**No 12m MOCA vs with 12m MOCA**
N	.	56	31	25	307	151	156	
Age (yrs)*	363	63.0 [54.0, 68.5]	62.0 [53.0, 67.0]	65.0 [55.0, 70.0]	64.0 [57.0, 72.0]	64.0 [57.0, 73.0]	64.5 [57.0, 72.0]	0.055
Sex, females	363	12 (21.4)	7 (22.6)	5 (20.0)	100 (32.6)	48 (31.8)	52 (33.3)	0.038
mRS > 1	363	21 (37.5)	10 (32.3)	11 (44.0)	64 (20.8)	33 (21.9)	31 (19.9)	0.0078
Onset to randomisation (days)*	363	52.0 [11.0, 249.5]	42.0 [10.0, 274.0]	57.0 [12.0, 101.0]	82.0 [31.0, 244.0]	91.0 [45.0, 238.0]	78.0 [20.5, 251.5]	0.18
Age completing education (yrs)	363	16.0 [15.0, 17.0]	16.0 [15.0, 16.0]	16.0 [15.0, 18.0]	16.0 [15.0, 18.0]	16.0 [15.0, 18.0]	16.0 [15.0, 18.0]	0.094
NIHSS (/42)	363	1.0 [0.0, 2.0]	1.0 [0.0, 2.0]	1.0 [0.0, 2.0]	0.0 [0.0, 2.0]	0.0 [0.0, 1.0]	0.0 [0.0, 2.0]	0.13
MOCA total (/30)	362	23.0 [20.0, 27.0]	24.0 [21.0, 27.0]	23.0 [18.0, 26.0]	27.0 [24.0, 28.0]	26.0 [25.0, 28.0]	27.0 [24.0, 28.0]	<0.0001
MOCA <=24	362	31 (56.4)	16 (53.3)	15 (60.0)	87 (28.3)	36 (23.8)	51 (32.7)	<0.0001
t-MOCA total (/22)	362	17.0 [14.0, 20.0]	18.0 [15.0, 20.0]	16.0 [13.0, 19.0]	19.0 [17.0, 21.0]	20.0 [17.0, 21.0]	19.0 [17.0, 21.0]	<0.0001
Trails B, time (seconds)	359	154.5 [105.0, 206.0]	158.5 [106.0, 200.0]	151.5 [93.0, 237.0]	102.0 [74.0, 158.0]	96.0 [73.0, 149.0]	105.0 [75.0, 171.0]	0.0010
Trail B, points	359	24.0 [15.0, 25.0]	24.0 [16.0, 25.0]	24.0 [15.0, 25.0]	25.0 [23.0, 25.0]	25.0 [23.0, 25.0]	25.0 [23.0, 25.0]	0.0053

Please note: Chi-square test was used for sex, mRS>1 and MOCA ≤ 24; Mann-Whitney U-test was used for all the continuous variables.

© 2023 Wardlaw JM et al. *JAMA Neurology*.

eTable 6. Comparison of Participants Without vs With Data on the Telephone Montreal Cognitive Assessment at 12 Months

C) ISMN and Cilostazol versus ISMN versus Cilostazol versus neither

		No 12m MOCA					With 12m MOCA					p
**Variable**	**N**	**All**	**Both**	**ISMN**	**Cil**	**None**	**All**	**Both**	**ISMN**	**Cil**	**None**	**No 12m MOCA vs with 12m MOCA**
N	.	56	15	16	16	9	307	76	74	75	82	
Age (yrs)*	363	63.0 [54.0, 68.5]	59.0 [51.0, 67.0]	67.0 [55.0, 72.0]	63.5 [56.5, 67.0]	61.0 [55.0, 65.0]	64.0 [57.0, 72.0]	64.0 [56.5, 72.0]	63.5 [56.0, 72.0]	64.0 [57.0, 75.0]	65.0 [58.0, 74.0]	0.055
Sex, females	363	12 (21.4)	3 (20.0)	3 (18.8)	4 (25.0)	2 (22.2)	100 (32.6)	24 (31.6)	24 (32.4)	24 (32.0)	28 (34.1)	0.038
mRS > 1	363	21 (37.5)	5 (33.3)	8 (50.0)	5 (31.3)	3 (33.3)	64 (20.8)	16 (21.1)	11 (14.9)	17 (22.7)	20 (24.4)	0.0078
Onset to randomisation (days)*	363	52.0 [11.0, 249.5]	42.0 [17.0, 421.0]	61.5 [21.0, 90.0]	34.0 [7.5, 198.0]	57.0 [6.0, 445.0]	82.0 [31.0, 244.0]	101.5 [50.5, 231.0]	77.5 [19.0, 264.0]	79.0 [39.0, 241.0]	78.0 [21.0, 244.0]	0.18
Age completing education (yrs)	363	16.0 [15.0, 17.0]	16.0 [15.0, 16.0]	15.5 [15.0, 17.0]	16.0 [15.0, 18.0]	16.0 [15.0, 20.0]	16.0 [15.0, 18.0]	16.0 [15.0, 18.0]	16.0 [15.0, 18.0]	16.0 [15.0, 17.0]	16.0 [15.0, 18.0]	0.094
NIHSS (/42)	363	1.0 [0.0, 2.0]	0.0 [0.0, 1.0]	1.0 [0.0, 2.0]	1.0 [0.5, 2.0]	1.0 [0.0, 2.0]	0.0 [0.0, 2.0]	0.0 [0.0, 1.0]	1.0 [0.0, 2.0]	0.0 [0.0, 1.0]	0.0 [0.0, 2.0]	0.13
MOCA total (/30)	362	23.0 [20.0, 27.0]	23.5 [19.0, 28.0]	23.0 [18.0, 26.0]	24.5 [23.0, 27.0]	23.0 [20.0, 27.0]	27.0 [24.0, 28.0]	26.0 [25.0, 28.5]	26.5 [24.0, 28.0]	26.0 [24.0, 28.0]	27.0 [24.0, 28.0]	<0.0001
MOCA <=24	362	31 (56.4)	8 (57.1)	9 (56.3)	8 (50.0)	6 (66.7)	87 (28.3)	15 (19.7)	23 (31.1)	21 (28.0)	28 (34.1)	<0.0001
t-MOCA total (/22)	362	17.0 [14.0, 20.0]	17.5 [14.0, 20.0]	16.5 [13.0, 19.0]	18.5 [15.0, 20.0]	16.0 [14.0, 19.0]	19.0 [17.0, 21.0]	20.0 [17.0, 21.0]	19.0 [18.0, 21.0]	19.0 [17.0, 20.0]	19.0 [16.0, 21.0]	<0.0001
Trails B, time (seconds)	359	154.5 [105.0, 206.0]	167.5 [75.0, 200.0]	143.0 [105.0, 206.0]	156.5 [126.0, 188.0]	180.0 [81.0, 295.0]	102.0 [74.0, 158.0]	91.5 [70.5, 143.5]	106.0 [74.0, 161.0]	98.5 [74.0, 157.0]	105.0 [77.0, 171.0]	0.0010
Trail B, points	359	24.0 [15.0, 25.0]	23.5 [13.0, 25.0]	24.0 [15.0, 25.0]	25.0 [20.5, 25.0]	24.0 [13.0, 25.0]	25.0 [23.0, 25.0]	25.0 [23.0, 25.0]	25.0 [23.0, 25.0]	25.0 [22.0, 25.0]	25.0 [24.0, 25.0]	0.0053

Please note: Chi-square test was used for sex, mRS>1 and MOCA ≤ 24; Mann-Whitney U-test was used for all the continuous variables.

© 2023 Wardlaw JM et al. *JAMA Neurology*.

eFigure 1. Recruitment Graph

Recruitment commenced with the 1st participant randomized on 5th Feb 2018 and ended with the last randomization on 31st May 2021 with 363 participants. Recruitment and randomization stopped completely for four months in 2020 due to COVID 19 and resumed, albeit more slowly, in July 2020.

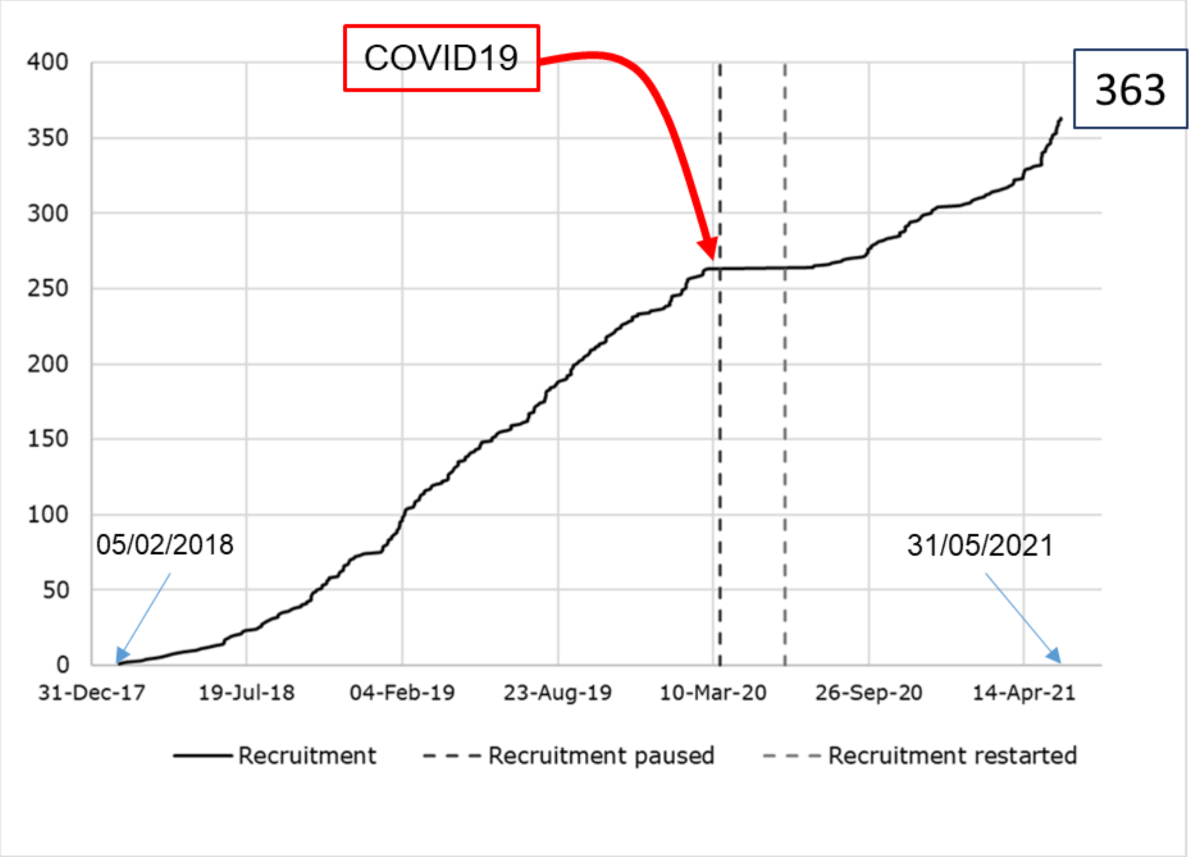

© 2023 Wardlaw JM et al. *JAMA Neurology*.

eFigure 2. Modified Rankin Scale (mRS) at 12 Months, Ordinal Shift Distributions

a) ISMN versus no ISMN, adjusted odds ratio 0.67 (0.45, 1.02), p=0.060.

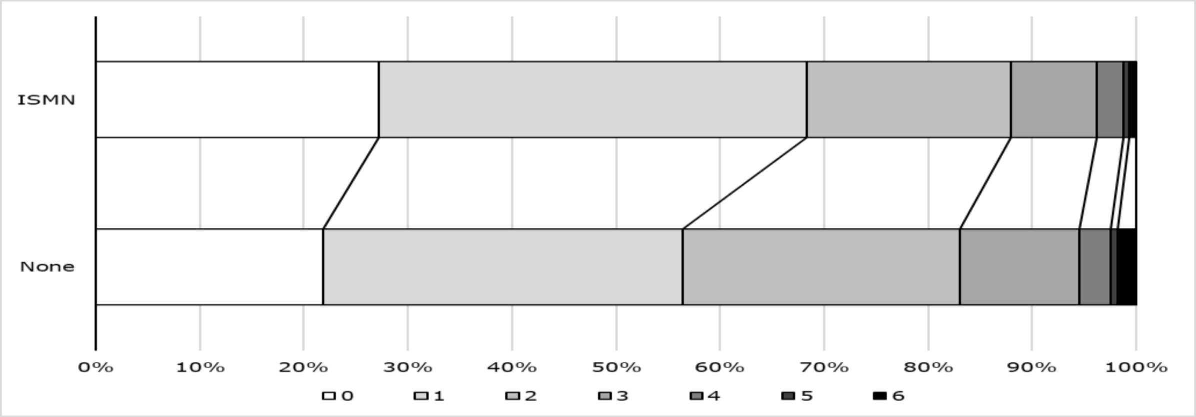

b) Cilostazol versus no cilostazol, adjusted odds ratio 0.85 (0.56, 1.28), p=0.44.

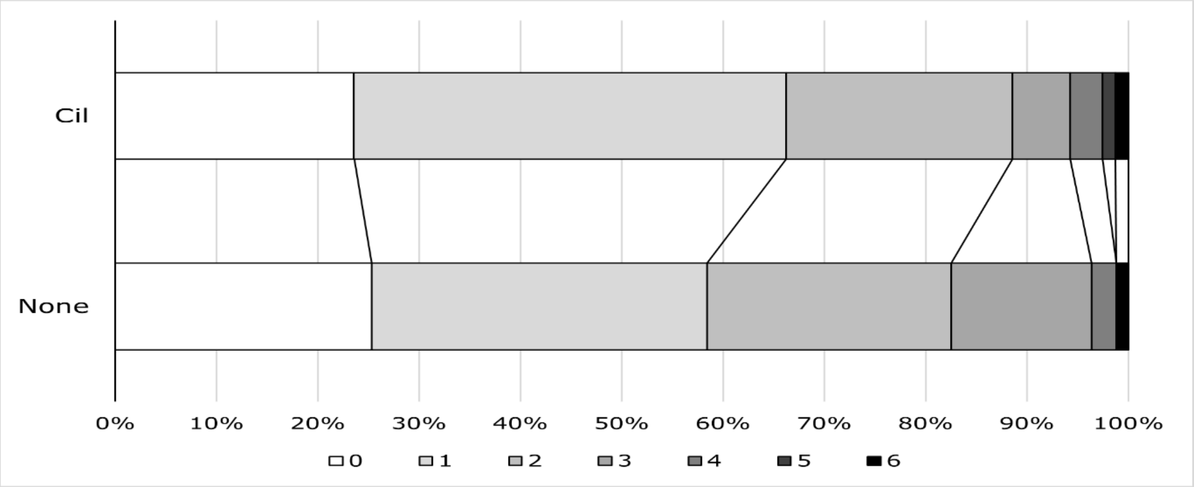

c) Combined ISMN and Cilostazol versus neither drug, adjusted odds ratio 0.51 (0.28, 0.93), p=0.028.

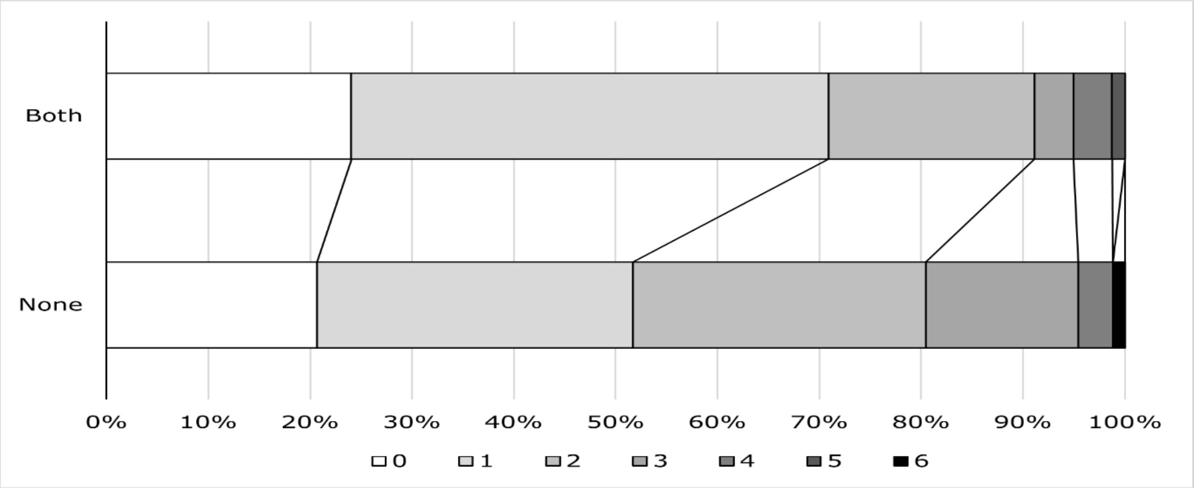

© 2023 Wardlaw JM et al. *JAMA Neurology*.

eFigure 3. Composite Outcome (Recurrent Stroke or TIA or MI, Any Cognitive Impairment, Dependency mRS >2, Death) by Subgroups

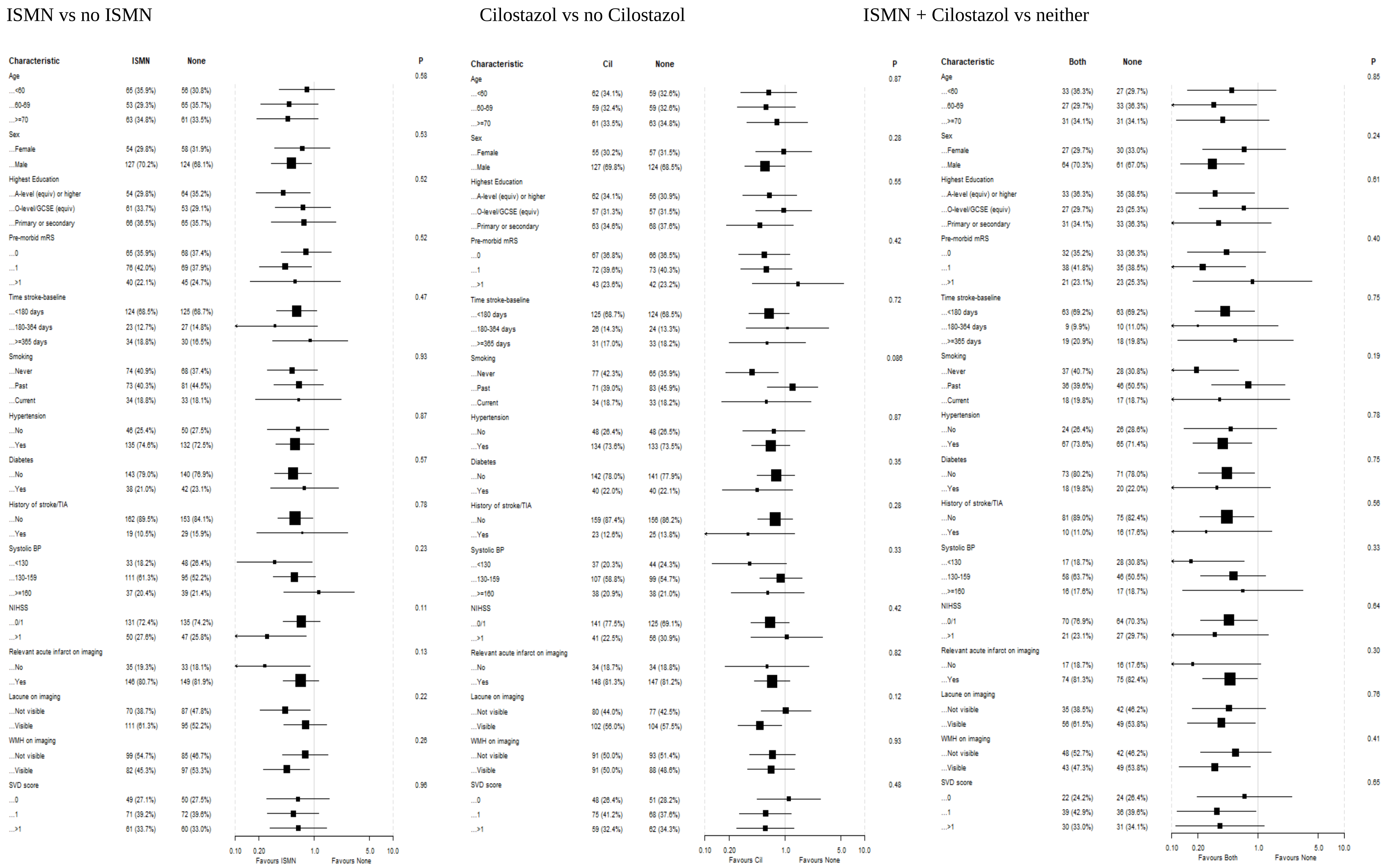

© 2023 Wardlaw JM et al. *JAMA Neurology*.

eAppendix. LACunar Intervention Trial-2 (LACI-2) Investigator Group

Chief Investigator: Joanna M Wardlaw

Co-Chief Investigator: Philip M Bath

Grant Co-Applicants/Project management group: Vera Cvoro, Fergus Doubal, Timothy England, Ahamad Hassan, Alan Montgomery, Nikola Sprigg, Joanna Wardlaw, David Werring.

Trial steering committee:

Independent Scientific Members: John Bamford (Chair), John O'Brien, Christine Roffe

Independent Participant Representative: Euan Haig

Non-Independent Members: Shannon Amoils, Philip M Bath, Vera Cvoro, Fergus Doubal, Timothy England, Ahamad Hassan, Anna Heye, Iris Mhlanga, Alan Montgomery, Katherine Oatey, Fiach O'Mahony, Nikola Sprigg, Joanna M Wardlaw, David Werring, Lisa Woodhouse.

Observers: Jason Appleton, Gordon Blair, Kaye Ferguson, Anna Foster, Sian Irvine, Zhe Kang Law, Eleni Sakka, Carol Williams.

Data monitoring committee: Colin Baigent (Chair), Gary Ford, Jonathan Emberson, Alison Murray (inception – 2021), A Ross Naylor (2021 - end)

Independent Blinded Event Adjudication: Fergus Doubal, Nikola Sprigg, Kailash Krishnan

International Advisors: Oscar Benavente (University of British Columbia, Vancouver, Canada), Hughes Chabriat (Université de Paris, Paris, France), Kazunori Toyoda (National Cerebral and Cardiovascular Center, Osaka, Japan)

Edinburgh Trial Staff

Edinburgh Clinical Trials Unit

Trial Managers, Co-ordinators: Julia Boyd 01/11/2017– 31/05/2018, Anna Heye 14/05/2018 – 30/11/2020, Katherine Oatey 01/12/2020-end (Trial Managers), Christine Campbell, Kaye Ferguson, Anna Foster, Sian Irvine, Katherine Lewis, Lynne McGillivray, Nicole Porter, Pamela Sinclair, Joyce Thomson.

University of Edinburgh and Centre for Clinical Brain Sciences related staff: Debbie Alexander, Susan Begg, Kate Covil, Judy Coyle, Debbie Hamilton, Guen Innes, Helen Watters, Carol Williams.

Image Data Management: Eleni Sakka, Jeb Palmer, David Buchanan.

Nottingham Stroke Trials Unit

Managers, Co-ordinators: Sharon Ellender, Robert Gray, Diane Havard, Olivier Matias, Patricia Robinson, Cameron Skinner.

Programmers: Lee Hayward, Richard Dooley, Athfi Rijal Mufied.

Statisticians: Lisa Woodhouse, Iris Mhlanga

Nottingham Clinical Trials Unit

© 2023 Wardlaw JM et al. *JAMA Neurology*.

Unblinded Statisticians (supporting DMC): Cydney Bruce, Wei Tan

Participating Sites:

Edinburgh, Royal Infirmary, C001: Fergus Doubal (PI), Carmen Arteaga Reyes, Gayle Barclay, Gordon Blair, Brittany Bovenzi, Ruaridh Buchan, Seona Burgess, Una Clancy, Michelle Coakley, Angie Crawford, Sean Denham, Nicholas Fethers, Iona Hamilton, Charlotte Jardine, Lucy Kessler, Allan MacRaild, Donna McIntyre, Emma Moatt, Rachel Penman, Rustam Al-Shahi Salman, Elaine Sandeman, Jennifer Tait, Pat Taylor, Jessica Teasdale, William Whiteley Recruited: 52 Participants

Nottingham, University Hospitals, C002: Kailash Krishnan (PI), Jason Appleton, Philip M Bath, Amanda Buck, Lucy Fleming, Sheila Hodgson, Ashah Kumar, Zhe Kang Law, Carla Richardson, Rosie Roberts, Nikola Sprigg, Gemma Squires, Gwen Wilkes. Recruited: 23 Participants

Kirkcaldy, Victoria Hospital, C004: Vera Cvoro (PI), Mandy Couser Recruited: 43 Participants

Glasgow, Queen Elizabeth Hospital, C005: Jesse Dawson (PI), Sandee Beattie, Alan Cameron, Ozlem Dincarslan, Laura Dymock, Helen Hart, Tracey Hopkins, Pamela MacKenzie, Belinda Manak, Lesley McDonald, Evonne McLennan, Karen Montgomery, Azmil Abdul-Rahim, Colin Rodden, Jennifer Tait, Linda Taylor, Nicola Tynan, Rosie Woodward. Recruited: 18 Participants

Bradford, Royal Infirmary, C006: Chris Patterson (PI), Eman Abdussami, Ruth Bellfield, Numan Khan, Stuart Maguire, Outi Quinn, Kelvin Stewart. Recruited: 13 Participants

Aberdeen Royal Infirmary, C007: German Guzman Gutierrez (PI), Jacqueline Furnace, Janice Irvine, Mary J MacLeod, Vicky Taylor, Sandra Williams. Recruited: 12 Participants

Leeds General Infirmary, C008: Ahamed Hassan (PI), Nathan Douglas, Ana Garcia, Luis Idrovo, Linetty Makawa, Vasileios Papavasileiou, Marc Randall, Emelda Veraque, Dean Waugh. Recruited: 20 Participants

Derby, Royal Derby Hospital, C009: Timothy England (PI), Wendy Abbott, Sathon Boonyapragon, Jessica Beavan, Liz Bedford, Richard Donnelly, Michelle Fanuncio, Marie Goldsworthy, Margaret Harper, Amanda Hedstrom, Katie Large, Peter Mason, Lisa Mayles, Harry McNaughton, Beverley Rushton, James Scott, Hege Strand, Kashmira Subramanian Shenila Zaya. Recruited: 13 Participants

Inverness Raigmore Hospital, C010: Stephen Makin (PI), Fiona Barrett, Clare Bradley, Jim Finlayson, Ashish Macaden, Joanna Matheson, Debbie McDonald, Mary Mckenzie, Donna Patience, Lesley Sanders, Adam Scotson, Zoe Urquhart. Recruited: 5 Participants

London, St George's Hospital, C011: Usman Khan (PI), Rebecca Williams Recruited: 21 Participants

London, Kings College Hospital, C012: Laszlo Sztriha (PI), Thomas Booth (PI), John Aeron-Thomas, Myriam Aissa, Sandeep Ankolekar, Sara Bik, Ajay Bhalla, , Frederick Boyle, Stuart Chandler, Staci Conway, Mena Farag, Maria Garcia-Pardo, Chong Ho, Fiona Humphries, Madawi Ismail, Agnieszka Kieliszkowska, Yee Mah, Esther Makanju, Dulka Manawadu, Georgina Meredith, Erika Manolo, Gaynor Notcheva, Elliott Smith, James Teo, Evangeline Theochari, Maria Consuelo Tibajia. Recruited: 16 Participants

Essex, Broomfield Hospital, C013: Amanathan Kirthivasan (PI), Abdulraza Alsowmely, Emma Cannon, Caroline Fox, Victoria Mead, Iman Mohamed. Recruited: 12 Participants

Stockton-on-Tees, University Hospital of North Tees, C014: Anwar Ijaz (PI), Arunkumar Annamalai, Vicky Collins, Helen Dunn, Sarah Pitcairn, Alex Ramshaw, Lorna Shepherd, Elaine Siddle, Bill Wetherill Recruited: 7 Participants

Sheffield, Royal Hallamshire Hospital, C015: Kirsty Harkness (PI), Jon Gardner, Jo Howe, Christine Kamara, Emma Richards, Madalina Roman, Mary Sikaonga Recruited: 6 Participants

Sandwell, General Hospital, West Bromwich, C016: Sevasti Ispoglou (PI), Steven Billingham, Anne Hayes, Lindsay Hough, Steven Shanu Recruited: 8 Participants

Winchester, Royal Hampshire County Hospital, C017: Nigel Smyth (PI), John Duffy, Charlotte Eglinton, Lucy Engin, Carolyn Fitton, Isi Ikhurionan, Lauriane Kerwood, Lucy Sykes, Ela Tone Recruited: 3 Participants

© 2023 Wardlaw JM et al. *JAMA Neurology*.

London, University College, C018: David Werring (PI), Amy Ashton, Azra Banaras, Nina Bason, Jonathan Best, Cyrille Mae Cahoy, Kevin Cregg, Kirsty Dimond, Scheherazade Feerick, Nina Francia, Simon Heller, Fiona Humphries, Howell Jones, Arnab Mandal, Marilena Marinescu, Sandra Mascarenhas, Talal Mayhani, Sabaa Obarey, Temi Olusi, Nikita Parmar, Richard Perry, Graziella Quattrocchi, Anna Robinson, Sadia Saber, Yezen Sammaraiee, Nayema Tahmin, Kristian Warnes, Caroline Watchurst, Annick Williams. Recruited: 9 Participants

Harrow, Northwick Park Hospital, C019: Aravinth Sivagnanaratnam (PI), David Cohen (PI), Gbadebo Adewetan, Rachel Baldwin Cleland, Robert Ballantine, Lara Barcella, Rajaram Bathula, Daniel Brooks, Swati Chhabra, Bindiya Kerai, Matilda Lang, Mushiya Mpelembue, Jasmin Patel, Phillip Regelous, Sreena Sreedevi Raj, Nicholas Winterkorn, Priya Yesupatham. Recruited: 17 Participants

Luton, Luton and Dunstable NHSFT University Hospital, C020: Lakshmanan Sekaran (PI), Lankanatha Alwis, Jane Angus, Asaipillai Asokanathan, Caroline Fornolles, Frances Justin, Duke Phiri, Sakthiviel Sethuraman, Margaret Louise Tate. Recruited: 21 Participants

Doncaster Royal Infirmary, C021: Dinesh Chadha (PI), Fiona Dunning, Mark Fairweather, Rebecca Pugh, Jeannette Stretton, Deborah Walstow, Sayed Zafar Recruited: 11 Participants

Wolverhampton, New Cross Hospital, C022: Nasar Ahmad (PI), Jumoke Adeyemi, Ann Bentley, Emily Carter, Rachel Evans, Kenneth Fotherby, Rachael Jones, Kulbinder Kauldhar, Samuel Nyabam, Victoria Shakespeare, Angela Stevens, Chris Wharton, Angela Wilberry, Jiao Yunzheng. Recruited: 9 Participants

Halifax, Calderdale Hospital, C023: Pratap Rana (PI), Megan Collins, Jill Greig, Mohammad Irfan Azam, Manohar Kini, Adam Mawer, Tonicha Nortcliffe, Ridha Ramiz, Matthew Robinson, Ryan Shaw, Kathryn Smith, Hayley Webster. Recruited: 7 Participants

Taunton, Musgrove Park Hospital, C025: Malik Hussain (PI), Richard Burgess, Kimberley Gillman, Dumin Karunatilake, Tamlyn Russell, Tania Wainwright, Alison Whitcher, Esther Zebracki Recruited: 4 Participants

Southampton General Hospital, C026: Nic Weir (PI), Michelle Beveridge, Alex Blades, Sue Evans, Efe Evbuomwan, Tomas Hannam-Penfold, Jake Harvey, Sneha Jethwa, Richard Marigold, Mariya Shaji, Simon Smith. Recruited: 4 Participants

London, Homerton University Hospital, C027: Thomas Harrison (PI), Ayesha Begum, Hannah Bouattia, Rebecca Brady, Luisa Cabrero, Kiran Dahele, Catherine Holbrook, Mudassar Hussain, Derya Ovayolu, Nideya Sharif, Penelope Talelli, Helen White. Recruited: 2 Participants

Exeter - Royal Devon & Exeter Hospital, C028: Salim Elyas (PI), Angie Bowring, Alina Govier, Martin James, Samantha Keenan, Jessica Kubie, Paul Mudd, Jane Sword. Recruited: 7 Participants

© 2023 Wardlaw JM et al. *JAMA Neurology*.

eReferences

1. Wardlaw J, Bath PMW, Doubal F, et al. Protocol: The Lacunar Intervention Trial 2 (LACI-2). A trial of two repurposed licenced drugs to prevent progression of cerebral small vessel disease. *European Stroke Journal*. 2020/09/01 2020;5(3):297-308. doi:10.1177/2396987320920110

2. Bath PM, Mhlanga I, Woodhouse LJ, et al. Cilostazol and isosorbide mononitrate for the prevention of progression of cerebral small vessel disease: Baseline data and statistical analysis plan for the Lacunar Intervention Trial-2 (LACI-2) (ISRCTN14911850). *medRxiv*. 2022:2022.05.31.22275743. doi:10.1101/2022.05.31.22275743

3. Bath PM MI, Woodhouse LJ, Doubal F, Oatey K, Montgomery AA, Wardlaw JM. Cilostazol and isosorbide mononitrate for the prevention of progression of : baseline data and statistical analysis plan for the Lacunar Intervention Trial-2 (LACI-2) (ISRCTN14911850). *Stroke and Vascular Neurology*. 2022:svn-2022-001816. doi:10.1136/svn-2022-001816

4. Jenkinson C, Fitzpatrick R, Crocker H, Peters M. The Stroke Impact Scale: validation in a UK setting and development of a SIS short form and SIS index. *Stroke*. 2013 2013;44(9):2532-2535. In File. doi:STROKEAHA.113.001847 [pii];10.1161/STROKEAHA.113.001847 [doi]

5. Zung WW. A self-rating depression scale. *Arch Gen Psychiatry*. 1965 1965;12:63-70. Not in File.

6. Blair GW, Appleton JP, Law ZK, et al. Preventing cognitive decline and dementia from cerebral small vessel disease: The LACI-1 Trial. Protocol and statistical analysis plan of a phase IIa dose escalation trial testing tolerability, safety and effect on intermediary endpoints of isosorbide mononitrate and cilostazol, separately and in combination. *Int J Stroke*. Jan 01 2018;13(5):530-538. doi:10.1177/1747493017731947

7. Blair GW, Appleton JP, Flaherty K, et al. Tolerability, safety and intermediary pharmacological effects of cilostazol and isosorbide mononitrate, alone and combined, in patients with lacunar ischaemic stroke: The LACunar Intervention-1 (LACI-1) trial, a randomised clinical trial. *EClinicalMedicine*. May-Jun 2019;11:34-43. doi:10.1016/j.eclinm.2019.04.001

8. McHutchison CA, Cvoro V, Makin S, Chappell FM, Shuler K, Wardlaw JM. Functional, cognitive and physical outcomes 3 years after minor lacunar or cortical ischaemic stroke. *J Neurol Neurosurg Psychiatry*. Apr 2019;90(4):436-443. doi:10.1136/jnnp-2018-319134

9. Makin SD, Doubal FN, Shuler K, et al. The impact of early-life intelligence quotient on post stroke cognitive impairment. *Eur Stroke J*. Jun 2018;3(2):145-156. doi:10.1177/2396987317750517

© 2023 Wardlaw JM et al. *JAMA Neurology*.
