# Supplementary material for: Isosorbide Mononitrate and Cilostazol Treatment in Patients With Symptomatic Cerebral Small Vessel Disease: The Lacunar Intervention Trial-2 (LACI-2) Randomized Clinical Trial
Source: JAMA Neurol. 2023 May 24;80(7):682–92. doi: 10.1001/jamaneurol.2023.1526 (PMC10209826; doi:10.1001/jamaneurol.2023.1526)
Supplement: Supplement 3. — Nonauthor Contributors [file jamaneurol-e231526-s003.pdf]

Supplement 4. Nonauthor Collaborators
*First name, last name, and suffix (if applicable) are required and will appear in PubMed.

*Group Name(s): Lacunar Intervention Trial-2 (LACI-2) Investigator Group							
***First Name and Middle Initial(s)**	***Last Name**	***Suffix (eg, Jr, III)**	Academic Degrees	Institution	Location (city, state/province, country)	Role or Contribution, eg, chair, principal investigator	Group (if more than 1 Group listed in the byline) and/or Subgroup (eg, Steering Committee)
Colin	Baigent		PhD	University of Oxford	Oxford, UK	Chair, Data Monitoring Committee	Data Monitoring Committee
Gary	Ford		PhD	Oxford University Hospitals	Oxford, UK	Member, Data Monitoring Committee	Data Monitoring Committee
Jonathan	Emberson		PhD	University of Oxford	Oxford, UK	Member, Data Monitoring Committee	Data Monitoring Committee
Alison	Murray		PhD	University of Aberdeen	Aberdeen, UK	Member, Data Monitoring Committee	Data Monitoring Committee
A Ross	Naylor		MD	University of Leciester	Leicester, UK	Member, Data Monitoring Committee	Data Monitoring Committee
Kailash	Krishnan		PhD	Nottingham University Hospitals	Nottingham, UK	Principal Investigator	Local Recruiting Site
Jesse	Dawson		PhD	Queen Elizabeth Hospital, University of Glasgow	Glasgow, UK	Principal Investigator	Local Recruiting Site
Chris	Patterson		MRCP	Bradford Royal Infirmary	Bradford, UK	Principal Investigator	Local Recruiting Site
German	Guzman Gutierrez		MRCP	Aberdeen Royal Infirmary	Aberdeen, UK	Principal Investigator	Local Recruiting Site
Stephen	Makin		PhD	Raigmore Hospital	Inverness, UK	Principal Investigator	Local Recruiting Site
Usman	Khan		MRCP	St George's Hospital	London, UK	Principal Investigator	Local Recruiting Site
Laszlo	Sztriha		MRCP	Kings College Hospital	London, UK	Principal Investigator	Local Recruiting Site
Thomas	Booth		FRCR	Kings College Hospital	London, UK	Principal Investigator	Local Recruiting Site
Amanathan	Kirthivasan		MRCP	Broomfield Hospital	Chelmsford, Essex, UK	Principal Investigator	Local Recruiting Site
Anwar	Ijaz		MRCP	University Hospital of North Tees	Stockton-on-Tees, UK	Principal Investigator	Local Recruiting Site
Kirsty	Harkness		PhD	Sheffield, Royal Hallamshire Hospital	Sheffield, UK	Principal Investigator	Local Recruiting Site
Sevasti	Ispoglou		MRCP	Sandwell General Hospital,	West Bromwich	Principal Investigator	Local Recruiting Site
Nigel	Smyth		MRCP	Royal Hampshire County Hospital	Winchester, UK	Principal Investigator	Local Recruiting Site
Aravinth	Sivagnanaratnam		MRCP	Northwick Park Hospital	Harrow, UK	Principal Investigator	Local Recruiting Site
David	Cohen		MRCP	Northwick Park Hospital	Harrow, UK	Principal Investigator	Local Recruiting Site
Lakshmanan	Sekaran		MRCP	Luton and Dunstable NHSFT University Hospital	Luton, UK	Principal Investigator	Local Recruiting Site
Dinesh	Chadha		MRCP	Doncaster Royal Infirmary	Doncaster, UK	Principal Investigator	Local Recruiting Site

Supplement 4. Nonauthor Collaborators
*First name, last name, and suffix (if applicable) are required and will appear in PubMed.

***First Name and Middle Initial(s)**	***Last Name**	***Suffix (eg, Jr, III)**	Academic Degrees	Institution	Location (city, state/province, country)	Role or Contribution, eg, chair, principal investigator	Group (if more than 1 Group listed in the byline) and/or Subgroup (eg, Steering Committee)
Nasar	Ahmad		MRCP	New Cross Hospital	Wolverhampton, UK	Principal Investigator	Local Recruiting Site
Pratap	Rana		MRCP	Calderdale Hospital	Halifax, UK	Principal Investigator	Local Recruiting Site
Malik	Hussain		MRCP	Musgrove Park Hospital	Taunton, UK	Principal Investigator	Local Recruiting Site
Nic	Weir		PhD	Southampton General Hospital	Southampton, UK	Principal Investigator	Local Recruiting Site
Thomas	Harrison		MRCP	Homerton University Hospital	London, UK	Principal Investigator	Local Recruiting Site
Salim	Elyas		MRCP	Royal Devon & Exeter Hospital	Exeter, UK	Principal Investigator	Local Recruiting Site
