# Supplementary material for: Isosorbide Mononitrate and Cilostazol Treatment in Patients With Symptomatic Cerebral Small Vessel Disease: The Lacunar Intervention Trial-2 (LACI-2) Randomized Clinical Trial
Source: JAMA Neurol. 2023 May 24;80(7):682–92. doi: 10.1001/jamaneurol.2023.1526 (PMC10209826; doi:10.1001/jamaneurol.2023.1526)
Supplement: Supplement 4. — Data Sharing Statement [file jamaneurol-e231526-s004.pdf]

Data Sharing Statement

Wardlaw. Isosorbide Mononitrate and Cilostazol Treatment in Patients With Symptomatic Cerebral Small Vessel Disease. *JAMA Neurol*. Published May 24, 2023. doi:10.1001/jamaneurol.2023.1526

Data
Data available: Yes
Data types: Deidentified participant data, Data dictionary
How to access data: The CI, with approval from the TSC as necessary, will consider all reasonable requests to share individual participant data on provision of a protocol detailing aims, hypotheses, analyses, tables, figures and publication plan. Where possible, we will perform the analyses; alternatively, de-identified data and a data dictionary will be provided for remote analyses, subject to signed data access agreement.
When available: beginning date: 01-01-2025

Supporting Documents
Document types: None

Additional Information
Who can access the data: The CI, with approval from the TSC as necessary, will consider all reasonable requests to share individual participant data on provision of a protocol detailing aims, hypotheses, analyses, tables, figures and publication plan. Where possible, we will perform the analyses; alternatively, de-identified data and a data dictionary will be provided for remote analyses, subject to signed data access agreement.
Types of analyses: Specified purpose stated in a protocol detailing aims, hypotheses, analyses, tables, figures and publication plan. Where possible, we will perform the analyses; alternatively, de-identified data and a data dictionary will be provided for remote analyses, subject to signed data access agreement.
Mechanisms of data availability: Where possible, we will perform the analyses; alternatively, de-identified data and a data dictionary will be provided for remote analyses, subject to signed data access agreement.
